# Selective targeting of skin pathobionts and inflammation with topically applied lactobacilli

**DOI:** 10.1016/j.xcrm.2022.100521

**Published:** 2022-02-15

**Authors:** Sarah Lebeer, Eline F.M. Oerlemans, Ingmar Claes, Tim Henkens, Lize Delanghe, Sander Wuyts, Irina Spacova, Marianne F.L. van den Broek, Ines Tuyaerts, Stijn Wittouck, Ilke De Boeck, Camille N. Allonsius, Filip Kiekens, Julien Lambert

**Affiliations:** 1 University of Antwerp, Department of Bioscience Engineering, Groenenborgerlaan 171, B-2020 Antwerp, Belgium; 2 University of Antwerp, Department of Pharmaceutical, Biomedical and Veterinary Sciences, Laboratory of Pharmaceutical Technology and Biopharmacy, Universiteitsplein 1, B-2610 Wilrijk, Belgium; 3 University Hospital Antwerp/University of Antwerp, Department of Dermatology and Venereology, Wilrijkstraat 10, 2650 Edegem, Belgium

## Abstract

Tailored skin microbiome modulation approaches with probiotics are highly challenging. Here, we show that lactobacilli are underestimated members of the skin microbiota. We select specific strains of nomadic lactobacilli for their functional applicability on the skin and capacity to inhibit growth and inflammation by skin pathobionts. The strains are formulated as microcapsules for topical formulations and tested in patients with mild-to-moderate acne. The selected lactobacilli are able to reduce inflammatory lesions in a pilot and placebo-controlled study. Daily application for 8 weeks is associated with an *in vivo* temporary modulation of the microbiome, including a reduction in relative abundance of staphylococci and *Cutibacterium acnes*, and an increase in lactobacilli. The reduction in inflammatory lesions is still apparent 4 weeks after the topical application of the lactobacilli ended, indicating a possible additional immunomodulatory effect. This study shows that carefully selected and formulated lactobacilli are a viable therapeutic option for common acne lesions.

## Highlights

• Lactobacilli are underestimated members of the skin microbiota
• Using lactobacilli in topical formulations is challenging but promising
• Lactobacilli can reduce acne lesions after daily application on the skin

## Graphical abstract

**Figure:**
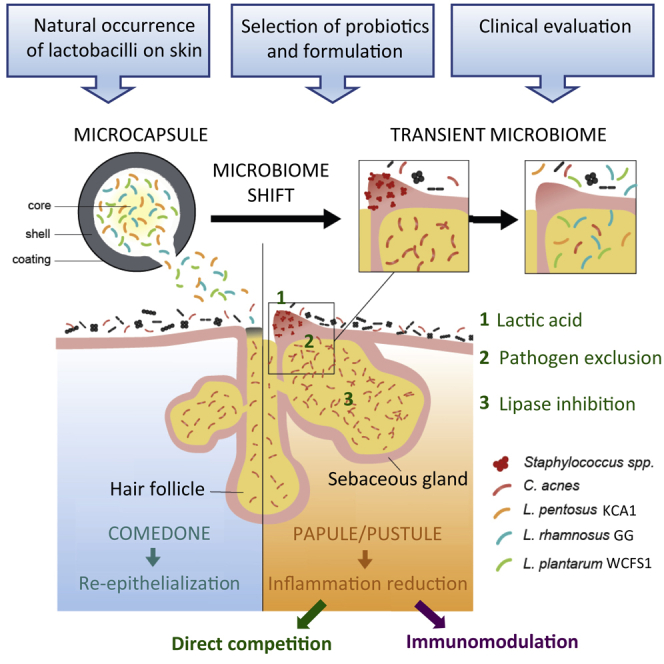

## Teaser

In their paper, Lebeer et al. describe that lactobacilli naturally occur on the skin and can be useful as probiotics for the skin. They carefully selected lactobacilli for microencapsulation and use in a topical cream and demonstrated its ability to modulate the skin microbiome and reduce acne symptoms.

## Introduction

Being the most extensive interface of the human body with the environment, the skin acts as a home to an important part of our commensal microbiota. Similar to the gut, the skin microbiome has essential roles in the education of our immune system and the protection against invading pathogens and other foreign substances. With recent advances in DNA sequencing approaches, our knowledge has improved on the biogeography of the skin microbiota at different body sites.^1^ We are now transitioning from these descriptive, observational studies toward a better understanding of the functional roles of the commensal microbiota, allowing the design of tailored modulation approaches. Compared with the richer environment of our intestines, the skin lacks many nutrients beyond basic proteins and lipids, with sweat, sebum, and the *stratum corneum* being main resources.^2^ In addition, the skin is a cool, acidic, and desiccated environment, and skin cells are frequently renewed and shed, so that strategies targeting the skin microbiome are highly challenging. Probiotics are live micro-organisms that, when administered in adequate amounts, confer a health effect on the host.^3^ They are generally applied in the gut, but the definition also holds true for skin applications, although other terms such as live biotherapeutic products or bacteriotherapy appear to be more preferred.^4^ Such strategies have not yet widely been considered for direct application on the skin, in a large part because of the technical challenges.^5^

One of the most common skin diseases is acne vulgaris, a chronic inflammatory skin condition of the sebaceous follicles and glands. The pathogenesis of acne vulgaris is multifactorial, with increased sebum production, alteration in the quality of sebum lipids, dysregulation of the hormone environment, and follicular hyperkeratinization as contributing factors. In addition, specific strains of the facultative anaerobe *Cutibacterium acnes* (formerly known as *Propionibacterium acnes*^6^) are involved in the inflammation of the skin, especially by secreting lipase enzymes that are able to metabolize sebum into free fatty acids, which may lead to skin irritation.^7^ Yet, the observation that almost all adults are colonized with *C. acnes* but only a minority have acne, highlights that other bacteria such as *Staphylococcus* species can be linked to acne pathogenesis as pathobionts or disease modulators.^8^ Therefore, both oral and topical antibiotics such as doxycycline, minocycline, and clindamycin are frequently used by acne patients,^9^ often also for their anti-inflammatory effects. However, because of the rising problems of antibiotic resistance, alternative therapies need to be developed.^10^

Here, we explored the potential of topically applied live lactobacilli to beneficially modulate cutaneous microbial interactions and host inflammatory responses in subjects with mild-to-moderate acne. Although lactobacilli have a long history of safe use in fermented foods,^11^ the gastrointestinal tract,^12^ urogenital tract,^13^ and nasal cavity,^14^ it was not certain whether lactobacilli could also thrive and have health-promoting activities on the skin and whether they could be developed in a skin cream in viable state.

## Results

### Detection of lactobacilli in skin microbiome samples

**Figure 1:**
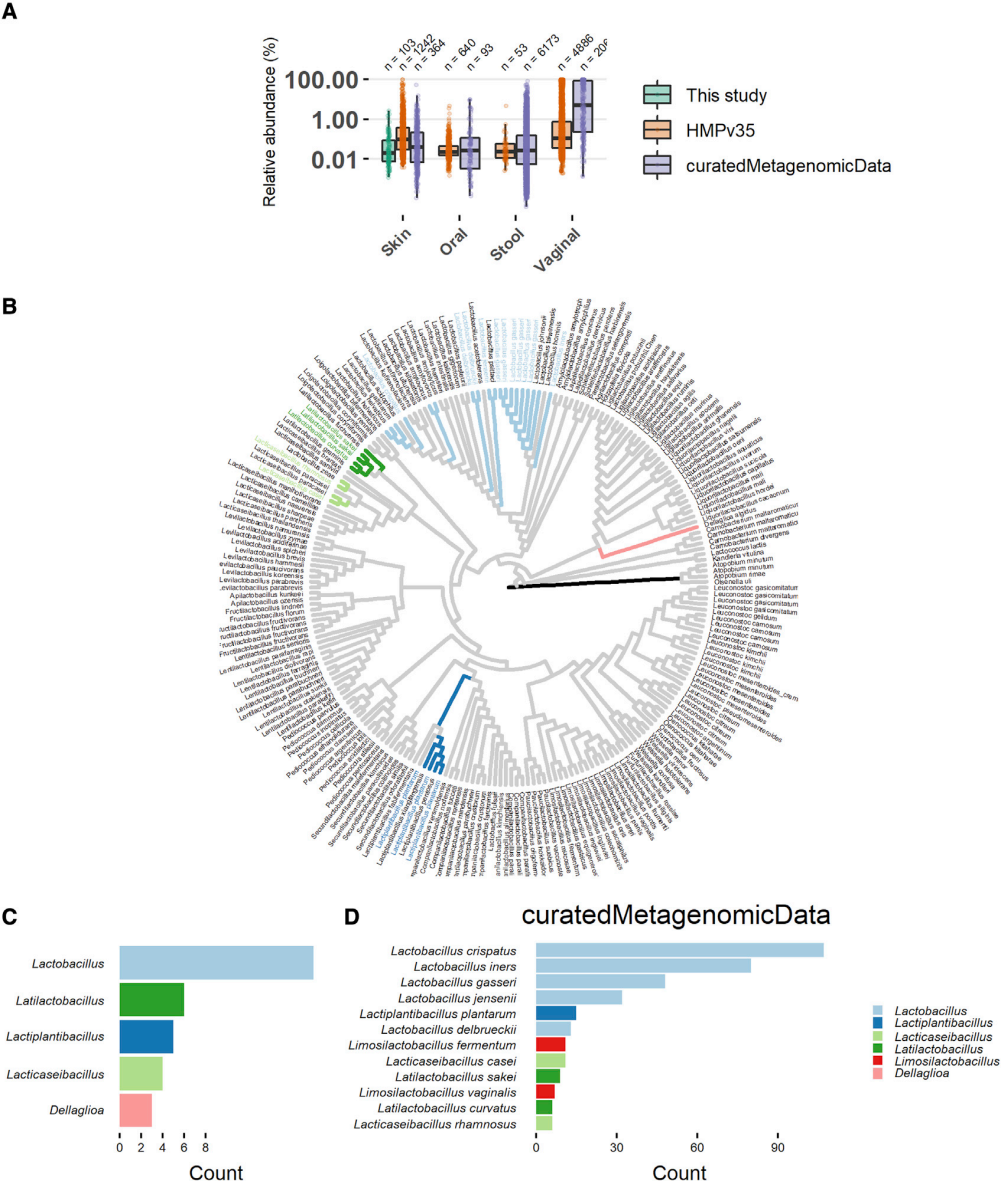
Taxa of lactobacilli found in skin samples of 16S rRNA amplicon and shotgun metagenomic data (A) Comparison of relative abundance of lactobacilli in different niches in the proof-of-concept study (merged two technical replicates), the Human Microbiome Project (HMPv35)^15^ and shotgun metagenomic datasets from three studies,^16^, ^17^, ^18^ accessed through the curatedMetaganomicData R Package. The y axis is represented in log scale.) (B) 16S rRNA cladogram of the Lactobacillaceae. Branches are colored based on phylogenetic placement of the ASVs of lactobacilli from this study and the phylogenetic group (as described by Duar et al.^19^) that they belong to. Tip labels are colored based on the 12 most abundant species of lactobacilli found in the skin shotgun metagenomic datasets. (C and D) The most abundant lactobacilli in this study (C) and the skin shotgun metagenomic datasets (D) colored according to the phylogenetic group they belong to

Because lactobacilli are not considered to be commensals of the skin, we first monitored the prevalence and relative abundance of lactobacilli on the skin of healthy volunteers. Their relative abundance was explored through 16S amplicon sequencing via Illumina MiSeq (separate runs for V1V2 and V4 variable regions) of facial skin samples (cheek). Thirty volunteers (15 male and 15 female, age ranged from 25 to 64 years old, median: 26), who did not display acne-related lesions, were included. In the samples of all female volunteers and 12 male volunteers, sequences classified as lactobacilli were found. They generally did not occur in the top five of most abundant taxa present on the skin. However, some volunteers showed a relative high abundance of lactobacilli taxa (amplicon sequence variants or ASVs), up to 6.4% (based on V1V2 16S sequencing) or 14.3% (by V4 16S sequencing) (Figures 1A, S1A, and S1B). The relative abundance of these taxa based on both runs (V1V2 and V4) was also 10-fold higher in women compared with men, with an average relative abundance of 0.8% (1.4% in women and 0.2% in men, Kruskal-Wallis p = 0.0005; Figure S1B). Lactobacilli-related taxa could thus be potential endogenous members of the skin microbiota, although their relative abundance is lower than that of *Staphylococcus*, *Corynebacterium, Cutibacterium* (often still classified as *Propionibacterium*), and *Streptococcus*, which were the most dominant taxa in our dataset for both variable regions sequenced (Figure S1A).

Nevertheless, skin microbiome samples are low in bacterial biomass and thus prone to contamination, either *in vivo* (e.g., when a healthy volunteer touches his/her face after having touched body sites or fermented foods rich in lactobacilli) or during the wet-lab procedure preparing the amplicon sequencing (e.g., from previous runs for vaginal microbiome samples). Therefore, to confirm our in-house-generated data and investigate whether our results were facial site-specific, the presence of lactobacilli was also substantiated in publicly available skin metagenome shotgun datasets by using the curatedMetagenomicData R-package.^20^ In total, 512 samples from six different skin studies^15^, ^16^, ^17^, ^18^, ^21^, ^22^^,^^21^^,^^22^ were analyzed. Of these samples, 38% (197/512) showed the presence of at least one species of lactobacilli, but only 36 skin samples showed a relative abundance higher than 1%. Yet high relative abundances up to 52% on the skin were also observed (average relative abundance based on curatedMetagenomicData was 0.05%; Figure 1A). We further included 16S amplicon data from the Human Microbiome Project (V3-V5),^15^ where a similar prevalence of lactobacilli (36%, 677/1881 samples) and relative abundance of 0.1% were observed (Figure 1A). The relative abundance of sequences of lactobacilli in the skin samples was also compared with the publicly available data of other human body sites (both 16S and shotgun metagenomes from curatedMetagenomicData; Figure 1A). As expected, the vagina showed the highest relative abundance of lactobacilli-related taxa, but the skin turned out to be the second most important niche for these taxa.

Moreover, to have a better idea of the phylogenetic diversity of all lactobacilli present on the skin, we plotted all data on a phylogenetic tree of the Lactobacillaceae^23^ (as recently taxonomically redefined) (Figure 1B). These data indicate that taxa typically associated with the human vagina, *Lactobacillus crispatus*, *L. iners*, *L. gasseri*, and *L. jensenii* were also found as the most prevalent lactobacilli on the skin (Figures 1C and 1D). Also, nomadic or free-living lactobacilli,^19^ namely species from *Latilactobacillus* (previously the *Latilactobacillus sakei* group), *Lactiplantibacillus* (previously *Lactobacillus plantarum* group), and *Lacticaseibacillus* (previously *Lactobacillus casei* group) were frequently detected (Figures 1C and 1D). The occurrence of lactobacilli on the skin is in agreement with the fact that after normal delivery through the birth canal, these bacteria originating from the vagina of the mother are among the first to colonize neonate skin.^24^ The data presented here (Figures 1A–1D) indicate that lactobacilli are still present in adults but do not stay dominant in the different human body skin sites studied. The detection of some nomadic lactobacilli on the skin suggests that some lactobacilli on the skin could also result from fermented food sources and thus be transient passengers. Yet their consistent presence and relative abundance between 0.05 and 1% in our own data and the publicly available data, did lead us to postulate that they could play a role as keystone microbes, recently redefined as taxa exerting a considerable influence on microbiome structure and functioning irrespective of their abundance across space and time.^25^ Therefore, we subsequently aimed to manipulate biotic interactions of lactobacilli on the skin by supplementing the endogenous lactobacilli with well-selected potential probiotic lactobacilli.

### Rationale for *in vitro* strain selection

**Figure 2:**
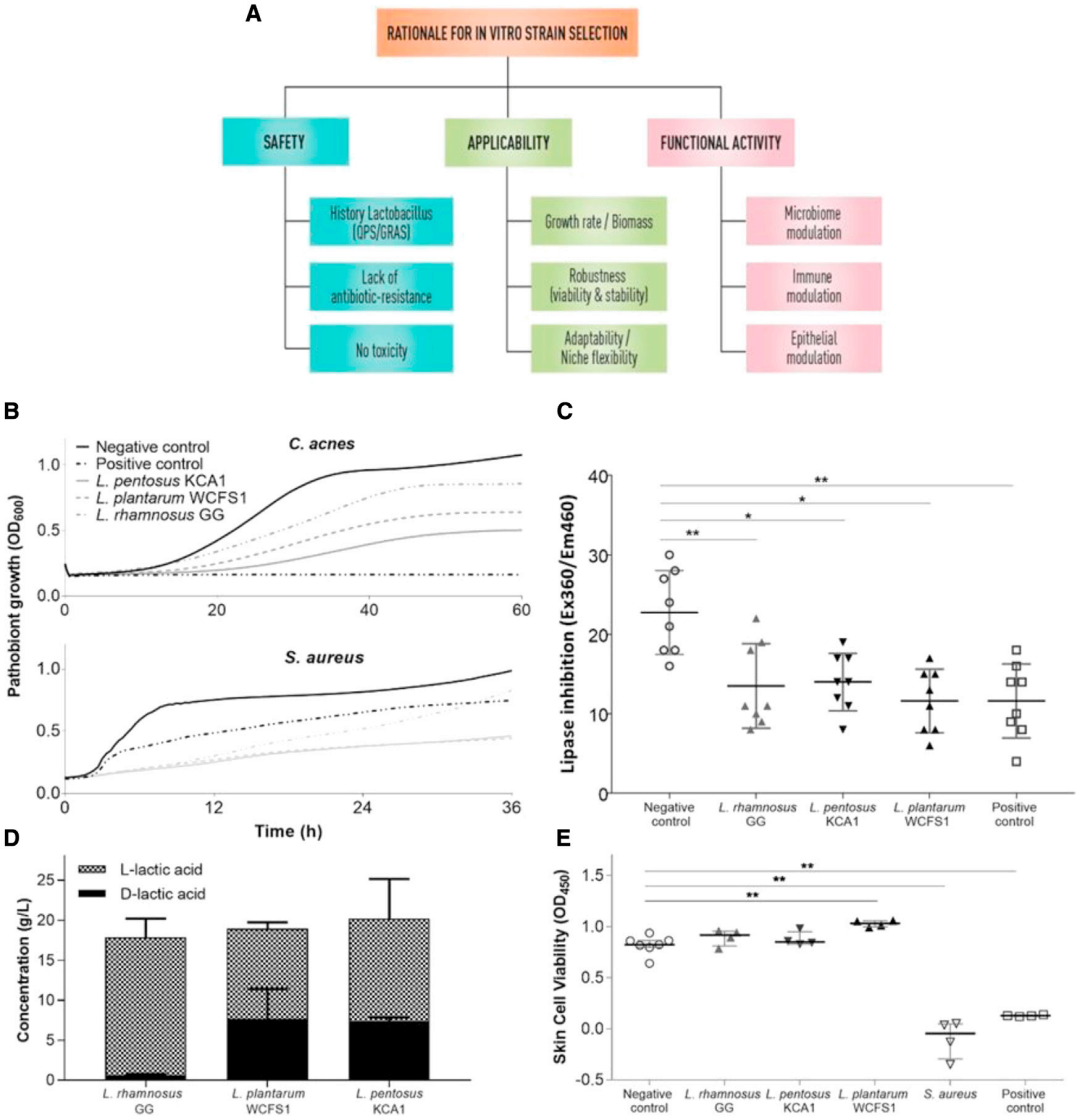
*In vitro* selection of lactobacilli for targeted application against acne vulgaris (A) Schematic overview of the rationale for the selection. Each criterion needs to be taken into account upon selection. Laboratory or genomic prediction tests exist for each criterion. More information can be found in the main text. (B) Antimicrobial activity of the spent-culture supernatant of the selected strains against the two pathobionts tested, *C. acnes* and *S. aureus*, and compared with the positive control (10 mg/mL Clindamycin, a common antibiotic used in acne). MRS at pH4, which is comparable to the pH of the spent supernatant of lactobacilli, was used as a negative control. Three technical replicates were combined to create the graphs. (C) Inhibition of lipase activity of *C. acnes* by the spent-culture supernatant of the selected *Lactobacillus* strains and compared with the positive control (10 mg/mL Clindamycin) and the negative control (MRS). Each shape represents one technical replicate (means +/- SD). (D) Concentration of L-lactic acid and D-lactic acid as key antimicrobial and skin-modulating molecules produced by the selected lactobacilli after overnight incubation in MRS broth. Each bar graph results from at least three technical replicates (means +/- SD). (E) Skin cell viability results of normal human epidermal keratinocytes after addition of the selected lactobacilli compared with the negative control keratinocyte growth medium 2, and positive controls, *S. aureus* and Triton X-, measured at 450 nm using an XTT assay. Each shape represents one biological replicate. Medians with interquartile ranges are also shown. Statistical analysis was performed using a Mann-Whitney test, where ∗p < 0.05 and ∗∗p < 0.01

Various strains of the Lactobacillaceae were selected from our in-house available laboratory collection (Table S1) for tailored application on the skin. A thorough screening approach was applied based on the rationale that the strains had to be safe, be applicable (being robust and showing lifestyle flexibility, as described for lactobacilli by Duar et al.^19^), and have the capacity to exert beneficial functions on the human skin, including microbiome modulation, immune modulation, and epithelial barrier enhancement (Figure 2A). Key properties were substantiated with laboratory tests, genome screening, and information available in the literature. Three strains were selected, i.e., *Lacticaseibacillus rhamnosus* GG, *L. plantarum* WCFS1, and *Lactiplantibacillus pentosus* KCA1. The rationale for these strains was based on their genome availability,^26^, ^27^, ^28^ knowledge of their epithelial interaction capacity,^29^^,^^30^ and their robustness and growth capacity (Figure S2A), in addition to information on their safety in humans after oral,^31^, ^32^, ^33^ nasal,^34^ and vaginal^35^ high-dose application. *L. rhamnosus* GG was also selected because of previous reports on its capacity to inhibit the toxic effects of *Staphylococcus aureus* on epidermal keratinocytes,^36^ its strain-dependent capacity to promote re-epithelialization via secreted proteins such as Msp1/2 (p40/p75),^37^ and to augment tight-junction barrier function in human primary epidermal keratinocytes^38^ and because of our previous experience with this probiotic strain.^29^ For microbiome modulation, *C. acnes* was targeted as a model pathobiont associated with the inflammatory character of acne vulgaris. *S. aureus* was also targeted as an important pathogen causing skin inflammation.^39^

When the activity of spent culture supernatant of our collection of lactobacilli was screened for antimicrobial effects on the growth of *C. acnes* in suspension, all strains tested inhibited the growth of *C. acnes* ATCC6919 and *S. aureus* ATCC29213, but *L. pentosus* KCA1 (vaginal origin) and *L. plantarum* WCFS1 (saliva origin) were among the strains tested able to exert the highest inhibition (Figures 2B and S2B). Other related strains tested, including *Staphylococcus epidermidis* 12,228, did not inhibit *C. acnes* growth (Figure S2). In addition, the selected strains of lactobacilli were able to significantly reduce the lipase activity of *C. acnes* (Figure 2C). These lipase enzymes are involved in inflammation of the skin induced by *C. acnes*, because they metabolize sebum into free fatty acids, which may lead to skin irritation.^7^ Furthermore, because lactic acid has a strong antimicrobial activity,^40^ it may be involved in maintaining the anti-inflammatory status of the skin and has a documented dose-dependent capacity to ameliorate the appearance of acne in dermatology.^41^ We therefore substantiated lactic acid production by the selected lactobacilli (Figure 2D). We also validated that the three selected lactobacilli did not exhibit toxic or overt inflammatory responses on primary skin cells (Figure 2E), in agreement with genome predictions,^26^, ^27^, ^28^ and also lacked known antibiotic-resistance genes (Resfinder^42^) on mobile elements and known virulence genes.^43^ Laboratory validation of antibiotic-resistance profiles according to the guidelines of the European Food Safety Authority (EFSA)^44^ confirmed a phenotypic lack of antibiotic resistance of concern. Furthermore, these assays confirmed that standard beta-lactam antibiotics could be used as fallback scenario in the (unlikely) event of a bacteremia when applied on the skin of patients. The fact that these properties can be checked on the basis of comparative genome analyses is a clear advantage that individual well-known strains have over undefined microbial mixtures such as fecal microbiota transplants, for which fatal cases were reported recently by the transfer of drug-resistant bacteria.^45^

### Viable probiotic formulation in O/W cream

**Figure 3:**
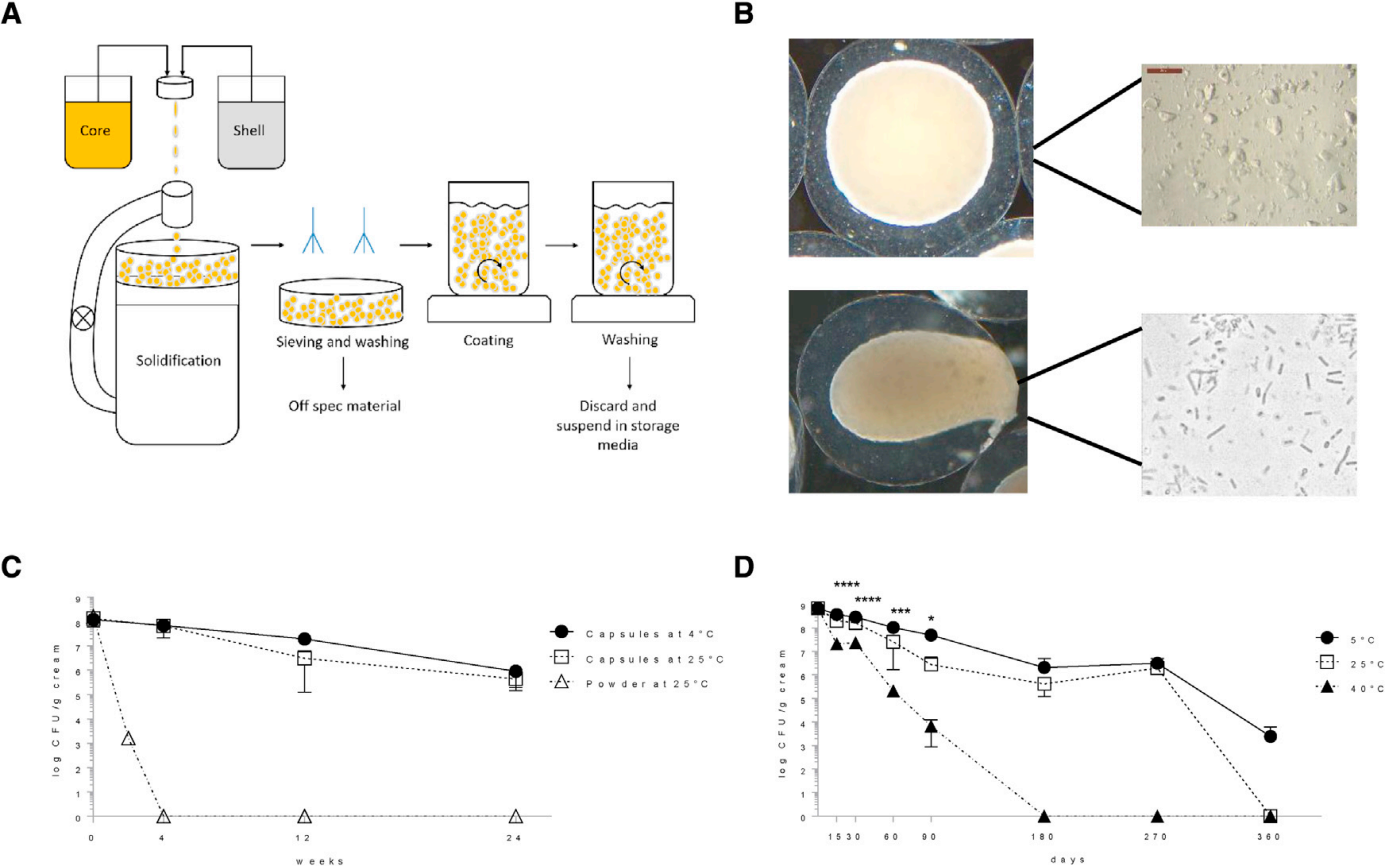
Formulating live lactobacilli in a topical cream (A) Schematic representation of the micro-encapsulating process with the bacteria in the core suspension and an outer shell made by the shell solution. (B) Resulting microcapsules with a core of freeze-dried bacteria suspended in oil compared with microcapsules in which a force is applied just before application on the skin, thereby releasing the bacteria and activating them through water uptake. (C) Survival of encapsulated bacteria, suspended in an O/W cream, compared with the non-encapsulated freeze-dried bacterial powder in the cream. Results are shown as mean with SD. (D) Survival of the encapsulated bacteria in O/W cream tested according to the International Council for Harmonisation of Technical Requirements for Pharmaceuticals for Human Use Q1A(R2). Results are shown as mean with SD. Statistical analysis was performed using a two-way ANOVA, where ∗p < 0.05, ∗∗∗p < 0.001, and ∗∗∗∗p < 0.0001.

We then aimed to design a topical formulation suitable for the application of live bacteria in a sufficient dose on the skin. The selected bacteria were freeze-dried for stability reasons^46^ and embedded in the core of two-compartment microcapsules (Figure 3A). Various processing conditions were optimized as described in the STAR Methods section and schematized in Figure 3A, resulting in capsules of 1,500–2,000-μm diameter with a core of suspended freeze-dried bacteria that could be released upon application of mechanical pressure, such as rubbing on the skin (Figure 3B). Ingredients were selected so that they did not significantly impact on the growth capacity of the encapsulated bacteria after release, skin commensals, and pathobionts (tested for *Staphylococcus epidermis*, *L. crispatus*, and *S. aureus*) (Figures S2C and S2D). This formulation and encapsulation approach significantly improved the viability for storage at 4°C and even at 25°C compared with non-encapsulated freeze-dried bacteria when suspended in a carrier oil-in-water (O/W) cream (Figure 3C) and this for up to 9 months (Figure 3D).

Subsequently, the skin irritation potential was checked on 20 healthy volunteers with skin patch tests according to Basketter et al.^47^ No erythema, dryness, or edema was observed in any of the volunteers studied (skin irritation index: 0.00) (Table S2). For comparison, adapalene products, which are naphthoic acid derivatives with retinoid activity and documented efficacy in the treatment of mild-to-moderate acne vulgaris, have a mean cumulative irritation index in healthy subjects with normal skin between 0.25 and 1.^48^ Also, the widely used combined clindamycin-benzoylperoxide treatment for moderate acne has been reported to frequently induce dry skin, flaky/peeling skin, irritated skin, itchy skin, and redness in acne patients.^49^

### Skin microbiome modulation with live lactobacilli

**Figure 4:**
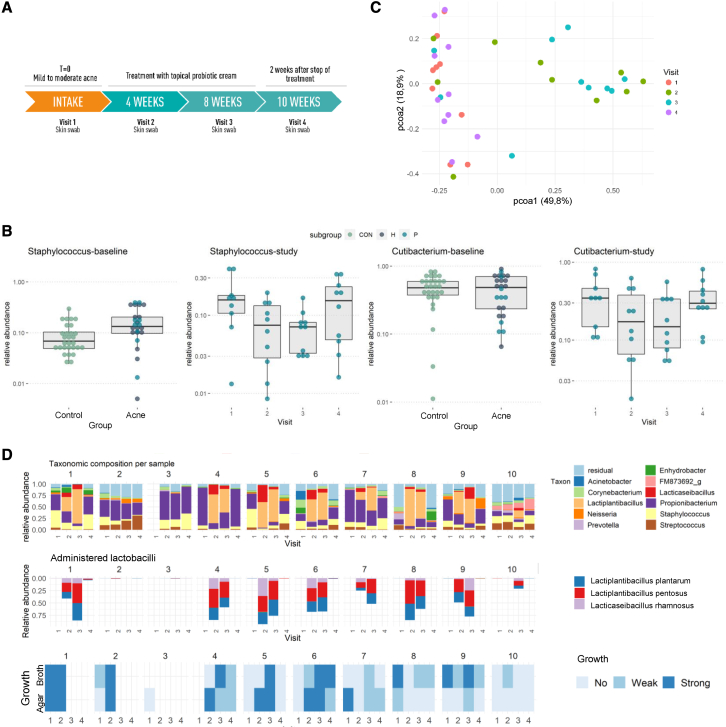
Impact of the lactobacilli cream on the skin microbiome: proof-of-concept study (A) Schematic overview of the proof-of-concept intervention study with the O/W cream containing the selected and formulated lactobacilli, the visits at which a skin swab was taken, and dermatological symptom analysis performed by the dermatologist. The cream was applied twice daily for 8 weeks with a minimal dose of 106 CFU per application. (B) Relative abundance of *Staphylococcus* and Lactobacillales respectively at baseline (left) and over the four visits of the study (right), resulting from 16S sequencing of the V1-V2 hypervariable regions. For the baseline, skin samples of the 30 healthy volunteers without acne symptoms (see Figure 1) and 27 patients with mild-to-moderate acne symptoms were compared. Of these 27 acne patients, 10 patients (indicated with light blue dots) were included in the intervention study with lactobacilli (Study) shown at the right side of each panel. Significant differences, as indicated by a pairwise Wilcoxon test with Holm correction for multiple testing, are indicated as ∗∗p ≤ 0.01 and ∗∗∗p ≤ 0.001. (C) PCOA plot distributing samples according to beta-diversity (Bray-Curtis distance). While the first dimension (x axis) was able to capture 49.8% of variation in the samples, a second dimension (y axis) shows 18.9% of variation. Similar samples are located closely to each other and colored by visit. (D) Microbial communities during the study period with the genus-level taxonomy indicated (top), relative abundance of the three ASVs resulting from the lactobacilli in the cream (middle), and observed growth on MRS medium (top row on agar [A], bottom row growth in MRS broth [B]) after addition of the skin samples (bottom). Other lactobacilli ASVs were not observed at a higher relative abundance than 1%. Samples were ordered by participant and by visit. For B and C, each sample (or dot) represents two merged technical replicates (if both passed QC).

Subsequently, we applied the topical cream in an open-label “proof-of-concept” longitudinal trial. Ten volunteers applied the cream with 10^8^ CFU of live lactobacilli per application (±1 g/application) for 8 weeks twice daily. Samples were taken before, during, and 2 weeks after the intervention (Figure 4A). Patients with mild-to-moderate acne symptoms that were not using antibiotics or another acne treatment were included by the responsible dermatologist (Table S3). The impact of the cream with lactobacilli on their facial skin microbiome was monitored by 16S amplicon sequencing of the V1 and V2 hypervariable regions at four different time points over a period of 10 weeks (Figure 4A). In this way, the skin baseline microbiome before, during, and after the treatment was compared. The skin acne microbiome of these patients at the time of inclusion was especially characterized by an increased relative abundance of *Staphylococcus* taxa (p = 0.0058, Wilcoxon rank sum test) compared with the healthy controls (Figure 4B) (Figure S3 for three specific *Staphylococcus* ASVs). After application of the cream with the lactic acid-producing lactobacilli, the facial skin samples of our acne patients at visit 2 (4 weeks) and visit 3 (8 weeks) clearly clustered separately on a PCOA plot (Figure 4C). Interestingly, in seven of 10 patients at visit two and eight of 10 patients at visit 3, the ASVs for lactobacilli were found in high relative abundances (between 20.9% and 92.8%), whereas in three patients at visit two and two patients at visit 3, their relative abundance was below 5% (between 0.015% and 1.1%; Figure 4D), with also a significant correlation between relative abundance of lactobacilli and comedones at visit 2 (Figure S4C). ASV analysis via the EzBioCloud database^50^ and comparison with the whole-genome sequences^26^, ^27^, ^28^ for rRNA copy variants confirmed that the detected ASVs matched the applied lactobacilli. Interestingly, the three probiotic strains appeared to persist on the skin in similar numbers (Figure 4D). To substantiate that the lactobacilli detected on the skin were still viable, samples were also plated on selective MRS agar for lactobacilli. Most samples at visit 2 (7/9) and visit 3 (6/7) were culture positive, indicating that (at least some of) the applied lactobacilli were metabolically active on the skin (Figure 4D). At visit 4 (2 weeks after the cessation of the treatment), most ASVs for lactobacilli had disappeared, and growth in MRS medium was also markedly reduced, further substantiating that the lactobacilli detected originated from the applied topical cream, although the (endogenously present) *L. delbrueckii* group (*Lactobacillus* genus *strictu sensu*) could still be detected. We then explored whether the presence of lactobacilli during treatment had impacted on the pathobionts of acne (*C. acnes* and *Staphylococcus* taxa). The relative abundance of both pathobiont taxa had indeed already dropped at visits 2 and 3 and increased again at visit 4 (p < 0.05 for visit three versus visit 1; Wilcoxon test for *Staphylococcus*; Figure 4B).

**Figure 5:**
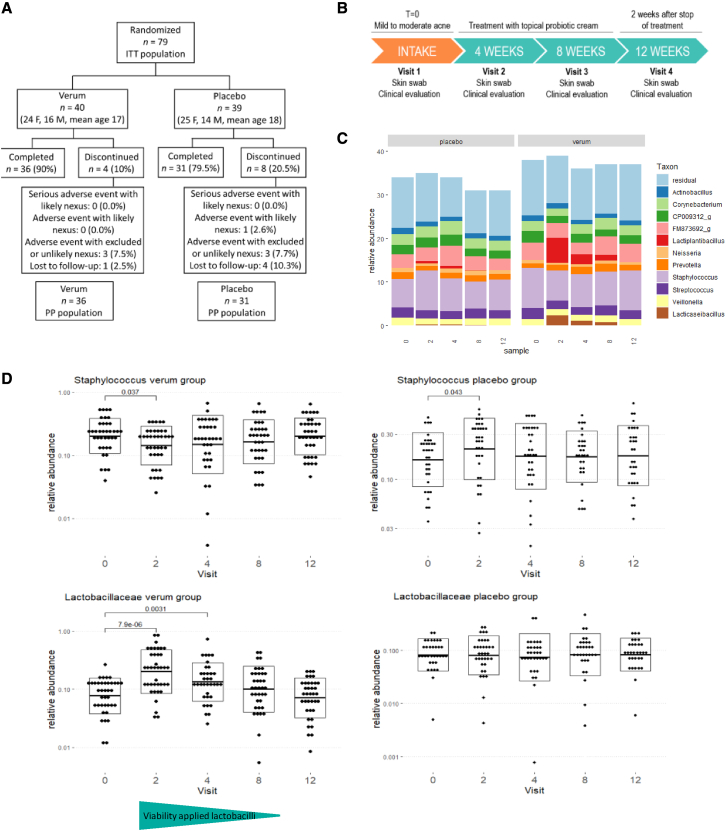
Impact of the lactobacilli cream on the skin microbiome: DBPC study (A) Flowchart of double-blind placebo-controlled (DBPC) study detailing enrollment and adverse events. (B) Schematic overview of the DBPC intervention study. The cream (verum or active) was applied twice daily for 8 weeks with a minimal dose of 10^6^ CFU per application for the verum cream. (C) Summary of microbial communities during the study period with the genus-level taxonomy indicated. Other *Lactobacill*aceae ASVs were not observed at a higher relative abundance than 1%. Samples were ordered by participant and by visit. (D) Relative abundances of *Staphylococcus* genus and *Lactobacillaceae* family (former *Lactobacillus* genus complex) during the study period, as found through 16S sequencing of the V4 hypervariable region.

**Figure 6:**
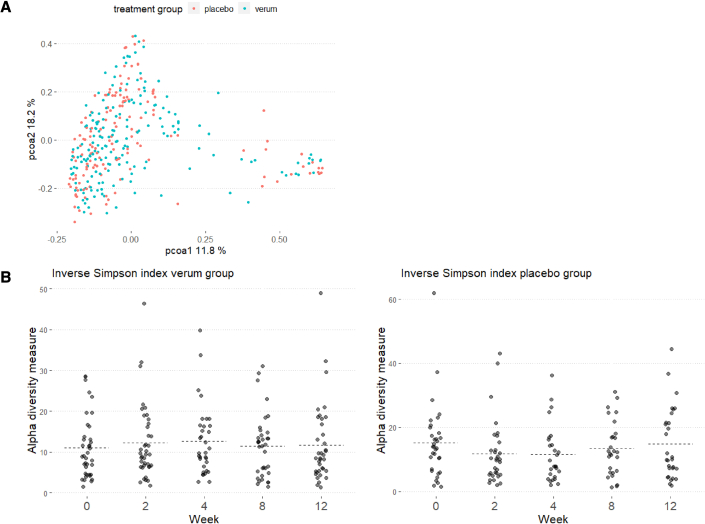
Impact of the lactobacilli cream on the skin microbiome diversity during the DBPC study (A) PCOA plot distributing samples according to beta-diversity (Bray-Curtis distance). Samples are colored by week of visit. Similar samples are located close to each other. (B) Alpha -diversity as indicated by Inverse Simpson Index (richness and evenness) and observed diversity (richness) throughout the study period, grouped by week of visit.

To confirm the observed skin microbiome modulation with live lactobacilli in the pilot open-label study, a double-blind placebo-controlled study was set-up including 79 acne patients with at least nine inflammatory lesions at the time of inclusion (12–33 years, all genders) (Figure 5A). Patients were randomized, and they applied either the active cream with encapsulated lactobacilli or the vehicle placebo, namely the same cream and microcapsules but without lactobacilli as active ingredient. The impact of the cream with lactobacilli on their facial skin microbiome was monitored by 16S amplicon sequencing at five different time points: before, during (after 2, 4, and 8 weeks), and 4 weeks after the intervention (Figures 5A and 5B). Notably, the swabs for these microbiome analyses were taken at least 1 h after the last application. During the intervention study, ASVs of Lactobacillaceae were found in high relative abundances in the verum group (Figure 5C). ASV analysis and comparison with the whole-genome sequences for rRNA copy variants again confirmed that the strains in the cream matched the ASVs in the sequencing data for 100%: *L. rhamnosus* GG matched the ASV *Lactobacillaceae* 1, while *L. plantarum* WCFS1 and *L. pentosus* KCA1 matched the ASV *Lactobacillaceae* 2. A significant increase in relative abundance of taxa of the Lactobacillaceae was observed at 2, 4, and 8 weeks. Of note, their relative abundance also decreased with time, which was in line with the decrease in viability of the lactobacilli in the cream during storage (Figure 5D). This was carefully monitored as part of the quality control and assurance. An increase of *Lactobacillaceae* ASVs was also observed in the placebo/vehicle group, suggesting that the cream formulation might also stimulate the colonization of endogenous lactobacilli on the skin, but this increase showed not to be significant. We then checked the relative abundance of acne pathobionts *C. acnes* and *Staphylococcus* taxa. A significant decrease in relative abundance of staphylococci was observed after 2 weeks of intervention (p < 0.05), in line with the highest relative abundance of lactobacilli at these time points (Figure 5D). No major impact on the relative abundance of *Cutibacterium spp.* was seen (Figure S5), although this could also have been due to the 16S rRNA V4 region. This region is not optimal for *Cutibacterium* but was chosen because of the focus on lactobacilli and staphylococci. We also explored whether the application of live lactobacilli has pathobiont-specific or more broad-acting microbiome effects (Figure S6). No significant impact of the lactobacilli intervention was found for the alpha diversity (measured by observed diversity and inverse Simpson index; Figure 6B). On the PCOA plots (Figure 6A) also, no clustering of the skin samples by treatment group or by time point could be observed. These observations are in line with specific effects of the lactobacilli cream on specific skin taxa without having a disrupting the microbiome balance.

### Lactobacilli-mediated improvement of acne symptoms

**Figure 7:**
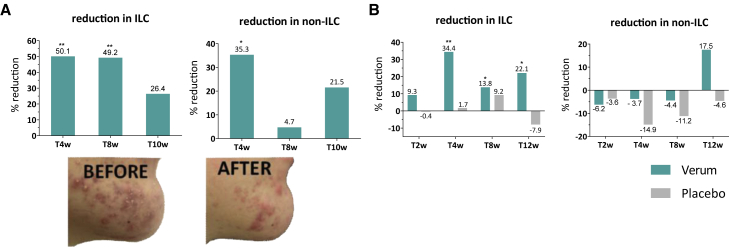
Effect of the lactobacilli cream on acne symptoms (A) Proof-of-concept study. The percent reduction in inflammatory lesion counts (ILC;, left) and non-inflammatory lesion counts (non-ILC; right) over the course of the study, grouped by visit (averages are shown). All 10 patients included in the pilot study showed a clinical improvement after the application of the cream, as exemplified by a picture of the acne spot area of one patient at T0 versus T4w. Written informed consent was obtained from the patient to use these photographs. (B) DBPC clinical trial. The percent reduction in ILC and non-ILC over the course of the study, grouped by visit. A significant reduction in ILC was seen at T4w, T8w, and T12w for the verum group and not for the placebo group, which confirms the anti-inflammatory capacity of the live lactobacilli. Averages are shown. Statistical analyses were performed using a Wilcoxon matched-pairs signed rank test, where ∗p < 0.05 and ∗∗p < 0.001.

Subsequently, since the acne pathogenesis is more than pathobiont overgrowth^51^ and probiotic effects often include anti-inflammatory effects at other body sites,^52^ other effects of the lactobacilli cream were evaluated. Acne lesions were clinically scored as the presence of inflammatory lesions and comedones. This analysis showed an overall improvement of the acne lesions in the patient group of the open-label pilot study (12–25 years, male) treated with the lactobacilli-supplemented cream, as reflected by a significant reduction in inflammatory lesions already at visits 2 (T [4 weeks]) and 3 (T8w [8 weeks]) compared with visit 1 (T0) and a significant reduction in comedone counts at visit 2 (Figure 7A). More importantly, the double-blind placebo group could confirm that the lactobacilli played a key role in the observed clinical effects of the pilot study. The patients who topically applied the live lactobacilli (n = 36 completed the trial) showed a significantly higher percentage of reduction in inflammatory lesions than the placebo group (n = 31 completed the trial) after 4 weeks (34.4% versus 1.7%), 8 weeks (13.7% versus 9.2%), and 12 weeks (22.1% versus −7.9%). These data indicate a rather fast-acting activity, since topical acne products often show significant differences from vehicle only after eight or 12 weeks of treatment.^53^ Most remarkably, the lactobacilli were able to maintain the reduction in inflammatory lesions after the treatment had been stopped, pointing toward a possible immunomodulatory effect involving the adaptive immune system. In addition, based on clinical and subjective signs, this study confirmed the exceptionally low irritation potential of both verum (2.8% with mild erythema at 4 weeks, 2.8% very mild scaling at 4 and 8 weeks) and placebo cream (3.2% with very mild scaling at 8 weeks, 3.2% with very mild itching at 12 weeks) (Tables S4 and S5). These data, with no adverse events reported, highlight that the topical cream also has a high safety profile in addition to its efficacy.

## Discussion

Acne vulgaris is a common reason for long-term antibiotic use, with dermatologists prescribing antibiotics more commonly than any other physician group.^9^ Here, we applied a multiphasic and multidisciplinary approach to substantiate that lactobacilli have potential as skin probiotics to target acne. First, we provided detailed information that lactobacilli are members of the human skin microbiota, with relative abundances between those of human vaginal^13^ and stool^12^ samples. Of interest, phylogenetic placement of the lactobacilli-related sequences detected in our data (ASVs) and public datasets showed that the dominant *Lactobacillus* taxa (*L. crispatus, L. iners, L. gasseri, L. jensenii*) of the vaginal community were also among the most prevalent taxa of lactobacilli of the skin. Previous studies have briefly acknowledged the presence of lactobacilli in the skin microbiota;^54^^,^^55^ however, such detailed analysis of specific taxa of lactobacilli in the skin niche had not yet been performed. We also noted that the prevalence (presence/absence) of *Lactobacillaceae* taxa in our dataset was higher than in the Human Microbiome Project dataset. This is probably due to the different approaches used, such as shotgun sequencing, which can have a bias against lower abundant taxa. This is further supported by the fact that the relative abundance of *Lactobacillaceae* in all datasets were similar, in line with the fact that our dataset was not notably biased toward *Lactobacillaceae*. Moreover, our detailed phylogenetic analyses of the lactobacilli detected is also of interest in view of the recent taxonomic changes in the *Lactobacillus* genus complex.^23^ These changes now allow for more functional studies on the habitat adaptation of specific taxa. Here, we show that the species *L. crispatus*, *L. iners*, *L. gasseri*, and *L. jensenii*, all still belonging to the genus *Lactobacillus strictu sensu*, have a broader human adaptation to stratified epithelium than merely the human vaginal epithelial cells, based on their association found here with (healthy) skin. However, it is possible that lactobacilli on the skin are transient passengers originating from food, the oral cavity, or the vagina, where they frequently occur. Nevertheless, it means the skin is (continuously) exposed to these taxa, and this exposure may have an impact on health, as we also aimed to study in this paper. Indeed, to exert a potential health benefit, the bacteria should not necessarily be endogenous stable members of the microbiota. Therefore, to apply selected lactic acid bacteria on the skin, not only functional properties were considered here, but also technical properties, because host-adapted microbes are often less able to survive and thrive outside the host. Here, we screened for robustness to (processing) stress conditions and good growth capacity, in addition to safety and lack of (transferable) antibiotic resistance properties, lactic acid production, inhibition of lipase activity by *C. acnes*, and growth of *S. aureus* as key probiotic properties (as rationalized in Figure 2A). The inhibition of lipase activity of *C. acnes* by lactic acid-producing lactobacilli had not yet been documented, but is in line with earlier research on the related cheese-associated *Propionibacterium* species that can utilize lactic acid as a nutrient and as an alternative metabolic pathway for lipolysis.^56^ Following this mechanistically driven screening, we did manage to translate our results directly from *in vitro* laboratory tests with skin cells and pathogens to human volunteers without the need for animal testing. The selected *L. rhamnosus* GG, *L. plantarum* WCFS1, and *L. pentosus* KCA1 could inhibit the growth of *C. acnes*, and *S. aureus in vitro* could survive the formulation in capsules in an O/W cream and were found in similar amounts on the facial skin of patients with mild-to-moderate acne. Twice daily topical application of this cream with live lactobacilli was able to reduce inflammatory acne lesions and comedone formation in the 10 patients included in an open-label pilot study and was associated with a specific reduction in *Staphylococcus* relative abundance but no overall impact on skin microbiome diversity (as summarized in Figure 7). A follow-up placebo-controlled trial confirmed that the lactobacilli formed the key active ingredient for the reduction of inflammatory lesions. Remarkably, this reduction in inflammatory lesions persisted 4 weeks after treatment cessation. 16S rRNA ASV-based comparison of the facial microbiome of 30 healthy volunteers and 27 patients with acne symptoms suggested indeed that *Staphylococcus* taxa are increased in acne patients and that *Staphylococcus* could thus form an interesting acne target to investigate further. This role of *Staphylococcus* needs more attention in future research on acne, because it is currently somewhat understudied in contrast to other skin disorders such as atopic dermatitis.^57^

ASV-level analysis of the sequenced V4 region of the 16S rRNA gene did not allow identification of the *Staphylococcus* taxa up to species/strain level, so that no distinction between *S. epidermidis* and *S. aureus* and more specific virulent strains should still be made. Yet, our results show that the nomadic or habitat-flexible lactobacilli chosen here from the *L. rhamnosus* and *L. plantarum* group could have long-term immunomodulatory effects against the *Staphylococcus* pathobionts and inflammation associated with acne, in line with the fact that beneficial effects observed in the clinical trial were still being observed 4 weeks after treatment stop. This points toward the stimulation of adaptive immune responses, such as increasing antibody production against the pathobionts, or immunoregulatory mechanisms helping to keep *S. aureus* in check. It will be very interesting in future experiments to explore whether the specifically selected lactobacilli could promote differentiation of a subpopulation of tissue-resident memory T cells (T_RM_) or other immunomodulatory interactions as has been described previously.^58^

### Conclusion

In this study, we show that live lactobacilli have probiotic potential for the skin and especially to reduce inflammatory lesions in acne. As compliance is key in acne management, the relatively fast reduction of the inflammatory lesions is an added value. The randomized placebo-controlled study demonstrated that the live lactobacilli are the key active pharmaceutical (or better pharmabiotic) ingredient responsible for the reduction in acne symptoms. Furthermore, the topical probiotic cream proved to be well tolerated and even to improve moisturization (skin hydration increased 37.3% after 14 days and 45.6% after 28 days of use), which is a unique property in the context of acne. We have performed in this study a detailed microbiological and molecular screening of the lactobacilli before their *in vivo* application on human skin of volunteers and patients, yet we acknowledge that more molecular studies are needed to unravel underlying immunomodulatory mechanisms in relation to the selected live lactobacilli and to translate the *in vivo* findings back to exact mechanisms. Further scrutinization of our results is necessary to contribute to a new era of skin therapeutics based on microbiome modulation as well as more fundamental and mechanistic insights into the possible keystone functions of lactic acid bacteria for skin health.

### Limitations of the study

The study represented here uses 16S amplicon sequencing of two regions as main approach for the microbiome analyses. Despite its many advantages, it also has some important limitations. First, although it provides quantitative information in terms of relative abundances, translating these compositional data to absolute abundances remains very challenging. Although qPCR can provide estimations of absolute concentrations, this approach is sensitive to contamination (also the case for amplicon sequencing in low-biomass samples as used here), aspecific binding of primers, suboptimal primer efficiency, and is only applicable to dedicated taxa without providing an overview picture, which is why we have not included that analysis here. Another limitation of 16S amplicon sequencing is that it does not allow taxonomic classification of the detected bacterial variant to species level with the necessary certainty (and therefore uses ASVs), so care should be taken when assigning species. Future work should for example include shotgun metagenomic sequencing to account for this. Furthermore, while we have mainly focused here on the activity of the lactobacilli to directly impact on skin pathogens/pathobionts and the skin microbiota, it is also possible that the lactobacilli indirectly influence the microbiota through immune modulation, as mentioned above. We have not monitored this in this study and therefore cannot provide information on the possible immunomodulatory mechanisms involved. In addition, we did not specifically investigate the (longitudinal) viability and activity of the applied lactobacilli on the skin, e.g., through RNA sequencing or metabolomics. Such approaches are very challenging for the skin. In the proof-of-concept study, we did include culture to show some viability and activity, but this analysis should be further substantiated in the future. Finally, we report two intervention studies with 10 (proof of concept) and 68 participants (RCT of which 31 were allocated to the placebo group), but to detect more subtle changes to the microbiota, larger study groups are needed.

## STAR★Methods

### Key resources table

**Table:** 

REAGENT or RESOURCE	SOURCE	IDENTIFIER
**Bacterial strains**		

*Lacticaseibacillus rhamnosus* GG	Willem de Vos (Kankainen et al., 2009)	ATCC53103
*Lactiplantibacillus plantarum* WCFS1	Kleerebezem et al. (2003)	WCFS1
*Lactiplantibacillus pentosus* KCA1	Anukam et al. (2013)	KCA1
*Lacticaseibacillus casei*	American Type Culture Collection (ATCC)	ATCC334
*Lacticaseibacillus casei shirota*	Commercial probiotic product	N/A
*Lacticaseibacillus casei* DN-114001	Commercial probiotic product	DN-114001
*Lactobacillus crispatus* LMG12005	BCCM/LMG	LMG12005
*Lactobacillus helveticus* 1807	Commercial probiotic product	1807
*Lacticaseibacillus paracasei* LMG12586	BCCM/LMG	LMG12586
*Lactiplantibacillus pentosus* ATCC8041	BCCM/LMG	ATCC8041
*Lactiplantibacillusplantarum* ATCC8014	BCCM/LMG	ATCC8014
*Lactiplantibacillusplantarum* 5057	Yoon et. Al (2017)	5057
*Limosilactobacillus reuteri* RC14	ATCC	RC14
*Lacticaseibacillus rhamnosus* GR-1	Segers et al. (2014)	GR-1
*Latilactobacillus sakei* ATCC15521	ATCC	ATCC15521
*Staphylococcus epidermidis* ATCC12228	ATCC	ATCC12228
*Cutibacterium acnes* (previously known as *Propionibacerium acnes*)	ATCC	ATCC6919
*Staphylococcus aureus*	ATCC	ATCC29213

**Biological samples**		

adult human skin swabs	University of Antwerp Universital Hospital Antwerp Allergisa, Brazil	N/A

**Chemicals, peptides, and recombinant proteins**		

de Man, Rogosa and Sharpe (MRS)	Difco	BD288210
Mueller Hinton	LabM limited	LAB039-A
Reinforced clostridial broth	LabM Limited	LAB023
Tween 80 (polysorbate)	Carl Roth Belgium	9139.1
Keratinocyt Growth medium 2	Promocell	C-20111
2,3-Bis(2-methoxy-4-nitro-5-sulfophenyl)-2H-tetrazolium-5-carboxanilide (XTT)	Sigma Aldrich	X12223
FloqSwab	Copan	502CS01
BacSwab	DME	1028
Trizma base	Sigma Aldrich	77-86-1
Ethylenediaminetetraaceticacid (EDTA)	Sigma Aldrich	60-00-4
Tween-20	Biorad	170-6531
PBS	Gibco	14040-091
SeaKem^R^ LE Agarose	Lonza	50004
1.0N NaOH	Illumina	NA
PhiX sequencing control v3	Illumina	FC-110-3001
Triton X-100	Sigma Aldrich	9036-19-5
Clindamycin	Sigma Aldrich	21462-39-5
PBS	Gibco	14040-091
4-methyl umbelliferyl oleate	Sigma Aldrich	75164-25MG
Tris-HCl	Roche	10812846001
NaCl	Carl Roth Belgium	3957.1
CaCl2	Carl Roth Belgium	A119.1
Sodium citrate	Carl Roth Belgium	2611.3

**Critical commercial assays**		

QIAamp Powerfecal DNA kit	Qiagen	12830-50
Agencourt AMPure XP	Beckman Coulter	A63881
NucleoSpin 96 Tissue kit	Machery-Nagel	MN 740609.50
D-Lactic acid/L-lactic acid kit	R-biopharm	11112821035

**Deposited data**		

DNA-Seq data	This paper	PRJEB27311 PRJEB44792
R code	This paper	https://github.com/LebeerLab/skin_acne_study
DNA-Seq data	The Human Microbiome Project Consortium (2012)	https://www.hmpdacc.org/

**Experimental models: Cell lines**		

Normal Human Epidermal Keratinocytes (NHEK)	Promocell	C-12001

**Oligonucleotides**		

Barcoded 515F primer (V4 primer) AAT GAT ACG GCG ACC ACC GAG ATC TAC **ACA TCG TAC G**TA TGG TAA TTG TGT GCC AGC MGC CGC GGT AA	Kozich et al. (2013)	N/A
Bacterial *16S rRNA* gene V4 region (806R) GGA CTA CHV GGG TWT CTA AT	Kozich et al. (2013)	N/A
Barcoded 27F primer (V1V2 primer) AAT GAT ACG GCG ACC ACC GAG ATC TAC AC**A AGC AGC A**TA TGG TAA TTC GAG AGT TTG ATC MTG GCT CAG	Suzuki et al. (1996)	N/A
Barcoded 388R primer (V1V2 primer) CAA GCA GAA GAC GGC ATA CGA GAT **ACC TAG TA**A GTC AGT CAG CCG CTG CCT CCC GTA GGA GT	Suzuki et al. (1996)	N/A

**Software and algorithms**		

GraphPad Prism	GraphPad Software	https://www.graphpad.com/
DADA2, version 1.6.0	Callahan et al. (2016)	https://benjjneb.github.io/dada2/index.html
R version 3.6.3 (R Core Team, 2020)	R Core Team (2020)	https://www.r-project.org/
Tidyamplicons	N/A	github.com/Swittouck/tidyamplicons
MicrobeDS R package	N/A	https://github.com/twbattaglia/MicrobeDS
curatedMetagenomicData R package	Pasolli, et al. (2017)	https://github.com/waldronlab/curatedMetagenomicData
Phyloseq	McMurdie & Holmes (2013)	https://github.com/joey711/phyloseq
EZBioCloud 16S database	Yoon et al. (2017)	https://www.ezbiocloud.net

**Other**		

MiSeq Desktop sequencer	Illumina	(M00984, Illumina)
Synergy HTX multi-mode reader	Biotek	N/A
Take3	Biotek	N/A
StepOne Plus Real-Time PCR System (v.2.0)	Applied Biosystems	N/A
EVE™ Automatic cell counter	NanoEntek	EVE-MC
Qubit 3.0 Fluorometer	Life Technologies	Q33216
Corneometer MPA 5 CPU	Courage and Khazala	S/N 10359198; probe S/N 11284693

### Resource availability

#### Lead contact

Further information and requests for resources and reagents should be directed to and will be fulfilled by the Lead Contact, Sarah Lebeer (sarah.lebeer@uantwerpen.com).

#### Materials availability

All unique/stable reagents generated in this study are available from the Lead Contact with a completed Materials Transfer Agreement.

### Experimental model and subject details

#### Human subjects

A proof-of-concept clinical trial was performed on patients with mild-to-moderate acne vulgaris included after careful assessment of the responsible dermatologist by counting of comedones and inflammatory lesions (Table S3). Patients were men between 12-25 years. Exclusion criteria were use of oral antibiotics within 4 weeks prior to start of the study and use of systemic retinoids within 6 months prior to start of study. A double-blind placebo-controlled clinical trial was performed on 79 patients wild mild -to-moderate acne vulgaris, aged from 12 to 33 years old (mean age: 18 years old), presenting at least 9 inflammatory lesions and oily skin on the face. Important exclusion criteria were the use of antibiotics within the last 4 months and acne treatments less than 90 months before the study (list of inclusion and exclusion criteria in Methods S1 and S2 and on ClinicalTrials.gov). All subjects provided written informed consent before the study began.

The protocol of this study was in accordance with the Declaration of Helsinki. The POC trial with the first patients was approved by the ethics committee of the University Hospital of Antwerp (Belgium) before initiation of the study. The study was given the approval number B300201628507 (Belgian registration) and registered online at clinicaltrials.gov with unique identifier NCT03469076. The second trial was approved by the Local Ethics Committee (LEC) of Investigation – Instituto de Pesquisas, registered by the National Research Ethics Commission (CONEP) of Brazil. The study was registered online at clinicaltrials.gov with unique identifier NCT04216160 (Methods S2).

#### Sample collection

Skin samples were collected by brushing the cheek (control group) or the affected area on the face (patients) with a FloqSwab (Copan) (first proof-of-concept study) or a sterile BacSwab (DME) soaked in saline solution (Tris, 50 mM pH 7.6; EDTA 1 mM pH 8.0; Tween 20 0.5%) over an area of ± 10 cm^2^ or around the lesions for 15 seconds. Swabs were then transferred to a falcon containing 800 μl Bead solution of QIAamp PowerFecal DNA kit (Qiagen). Samples were stored at 4°C until further processing (maximally 14 days). Before DNA extraction, samples were vortexed for 1 minute, after which the Bead solution was transferred to the bead tube. Subsequent steps of the DNA extraction were executed according to manufacturer’s instructions.

##### Proof-of-concept clinical trial

Patients were asked to apply the topical probiotic cream (containing 10^8^ CFU of each *Lactobacillus* strain per application of 1 g of the topical cream) twice daily for 56 days (8 weeks). The patients were seen by a dermatologist at start (before the therapy) (visit 1), week 4 (visit 2), week 8 (visit 3) and week 10 (visit 4). A skin swab was taken at each visit, total DNA was extracted and amplified for 16S amplicon sequencing as described above. Moreover, a clinical scoring was performed, and a photograph taken at each visit.

##### Double-blind placebo-controlled clinical trial

The study subjects remained in a room with controlled temperature and air relative humidity for at least 30 minutes before the initial measurements and in the interval between them. The initial clinical assessment was performed by a dermatologist to confirm the inclusion and exclusion criteria and the assess the initial state of the skin (Dermatological Clinical Assessment – IGA and DAT -T0). An acne lesions counting was performed by a trained technician. The subjects used the product for 8 weeks ± 2 days and then they suspended its use for 4 weeks ± 2 days. They were assessed before product use (T0), after 2, 4 and 8 weeks ± 2 days of product use (T2w, T4w and T8w, respectively) and after 04 weeks ± 2 days without product use (T12w). On all visits, an acne lesions counting was performed by a trained technician. The study subjects should record all the applications performed and add possible comments about the product (in a daily-log). They were also be instructed to perform the last product application on the previous day of the assessments at the institute.

### Method details

#### Bacterial growth

Strains of lactobacilli were grown at 37°C in de Man, Rogosa and Sharpe (MRS) medium (BD Difco, Erembodegem, Belgium). *Cutibacterium acnes* ATCC6919 was inoculated in reinforced clostridial broth (LabM Limited, Heywood, UK), supplemented with 0.2% Tween20 and cultured microaerobically (5% CO2) at 37°C. *Staphylococcus aureus* ATCC29213 was grown in Mueller-Hinton broth at 37°C. Solid media contained 1.5% (w/v) agar. Time-course experiments were also performed analysing the antimicrobial activity of spent culture supernatant (SCS) of the selected *Lactobacillus* strains against C. *acnes* and *S. aureus* ATCC29213 (cfr.^59^). Additionally, the impact of this SCS (10%) on the lipase activity of C. *acnes* was determined as previously described.^60^ Concentrations of D- and L-lactic acid were measured through a commercially available kit (R-Biopharm, Darmstadt, Germany) as previously described .^61^

#### Human skin cell culture

Normal human epidermal keratinocytes (NHEK) cells from juvenile foreskin from pooled donors were purchased from Promocell (Heidelberg, Germany) and cultured according to manufacturer’s recommendations in Keratinocyt Growth medium 2 (Promocell, Heidelberg, Germany). Cytotoxicity of probiotic strains was assessed using the 2,3-Bis(2-methoxy-4-nitro-5-sulfophenyl)-2H-tetrazolium-5-carboxanilide (XTT, Sigma-Aldrich) cell viability assay. NHEK cells were seeded at a density of 5000 cells/well in 96-well plates and cultured until confluent. Overnight cultures of probiotic strains or *S. aureus* were added to the wells with or without NHEK cells at 10^6^ CFU/well and incubated for 2 h at 5% CO2 and 37°C. Triton X-100 (0.5%) was used as a positive control.

#### Illumina MiSeq *16S rDNA* gene amplicon sequencing

The primers used for Illumina MiSeq sequencing were based on the previously described 27F-338R or 515F-806R primers^62^^,^^63^ and altered for dual-index paired-end sequencing, as described earlier^62^ (Key resources table). Separate runs were carried out for V1V2 and V4 *rRNA* gene variable regions. Quality control and processing of reads was performed using the R package DADA2, version 1.6.0.^64^ Denoised reads (amplicon sequence variants or ASVs) were merged and read pairs with one or more conflicting bases between the forward and reverse read were removed. Chimeric sequences were removed using the function “removeBimeraDenovo”. Finally, ASVs were classified from the kingdom to the genus level using the EzBioCloud 16S database.^50^ A species annotation was added to each ASV by listing the species of all 16S sequences in the database that showed an exact match to the ASV sequence. Contaminants were identified using the approach of Jervis-Bardy et al.^65^ ASVs with a strong negative correlation between relative abundances and total sample read counts were considered contamination. For each ASV, this correlation was calculated and tested for significance. ASVs with a p-value less than 0.0001 were removed. Samples were filtered by removing those with less than 1000 reads left after all read and ASV filtering steps. Results for negative controls (for both DNA extraction kits and PCR) can be found in Figure S7.

#### Biostatistical and bioinformatics analysis

Processing of the ASV table, ASV annotations (e.g. classification) and sample annotations (metadata) were performed using the in-house R package “tidyamplicons”, publicly available at github.com/SWittouck/tidyamplicons. For the analyses at the genus level, ASV read counts were aggregated at the genus level or, if unavailable, at the most specific level at which taxonomic annotation was available.

#### Analysis of public datasets

Processed OTU-table and sample metadata from the Human Microbiome Project (HMPv35)65 and the shotgun metagenomic datasets were retrieved using the MicrobeDS R package and curatedMetagenomicData R package^20^ respectively. All data was loaded, processed and visualized in the R-environment using Phyloseq.^66^ All scripts are available at https://github.com/LebeerLab/skin_acne_study.

#### Formulation of lactobacilli in microcapsules and O/W cream

A single colony of the three selected probiotic strains was grown until stationary phase and lyophilized (± 10^11^ CFU/gram) and subsequently encapsulated via a core-shell encapsulation approach. Briefly, the strains were mixed in equal amounts and homogeneously suspended to obtain a stable oil-based feed core suspension. The shell feed solution contained a hydrocolloid alginate polymer as gelling agent. Both liquid feeds were pumped to a concentric nozzle, to obtain a concentric fluid flow. The laminar liquid flow was broken up by a vibrational unit to obtain spherical -droplets that were solidified upon falling in a calcium-based solidification solution, forming the capsules. The collected capsules (10^9^ - 10^10^ CFU/gram) were washed and suspended in an oil-in-water cream. The ingredients of this cream, mainly the emulsifiers and preservatives, were selected to be compatible with the micro-capsules and bacteria, both during storage and upon release of the probiotics. Hereto, the impact of the topical cream without the capsules on the growth of four skin reference bacteria (*S. aureus, S. epidermidis, L. crispatus* and *C. acnes*) was evaluated at a concentration of 1, 10 and 100 mg/ml, by a time-course analysis of OD600 measurements as described above. Mechanical force (rubbing on the skin) was confirmed to break the capsules, releasing the inner core material containing the suspended probiotics. Skin irritation tests with the cream containing the encapsulated lactobacilli were performed as described previously.^47^

#### Determination of skin hydration (Corneometry)

The capacity to improve skin hydration of the cream with lactobacilli was tested after 14 and 28 days of use, twice daily, by 20 healthy volunteers (all female, average age 48,6, inner sides of forearms). Skin hydration was measured with Corneometer MPA 5 CPU (Courage and Khazaka, Cologne, Germany; S/N 10359198; probe S/N 11284693) by registering the electrical capacitance of the skin surface. Five measurements were performed on each test area and the mean was used to define the hydration state of the stratum corneum.

### Quantification and statistical analysis

Statistical details can be found in the figure legends. Statistical analyses were performed in R Studio, using a Mann-Whitney test, Two-way ANOVA or pairwise Wilcoxon test with Holm correction for multiple testing.

### Additional resources

The POC trial with the first patients was approved by the ethics committee of the University Hospital of Antwerp (Belgium) before initiation of the study. The study was given the approval number B300201628507 (Belgian registration) and registered online at clinicaltrials.gov with unique identifier: https://clinicaltrials.gov/ct2/show/NCT03469076?term=NCT03469076&draw=2&rank=1

The second trial was approved by the Local Ethics Committee (LEC) of Investiga – Instituto de Pesquisas, registered by the National Research Ethics Commission (CONEP) of Brazil. The study was registered online at clinicaltrials.gov with unique identifier: https://clinicaltrials.gov/ct2/show/NCT04216160?term=NCT04216160.&draw=2&rank=1

## Data Availability

• Sequencing data of the proof-of-concept clinical trial is available at the European Nucleotide Archive with the accession number PRJEB27311 (https://www.ebi.ac.uk/ena/browser/view/PRJEB27311?show=reads). • Sequencing data of the placebo-controlled clinical trial is are available with the accession number PRJEB44792 (https://www.ebi.ac.uk/ena/browser/view/PRJEB44792?show=reads). • The R code generated during this study can be found on GitHub at https://github.com/LebeerLab/skin_acne_study. • Any additional information required to reanalyze the data reported in this paper is available from the Lead Contact upon request.

## Supplemental information

Document S1. Figures S1–S7, Tables S1–S5, and Methods S1

Document S2. Article plus supplemental information
