# Supplementary material for: Selective targeting of skin pathobionts and inflammation with topically applied lactobacilli
Source: Cell Rep Med. 2022 Feb 15;3(2):100521. doi: 10.1016/j.xcrm.2022.100521 (PMC8861818; doi:10.1016/j.xcrm.2022.100521)
Supplement: Document S1. Figures S1–S7, Tables S1–S5, and Methods S1 [file mmc1.pdf]

Cell Reports Medicine, Volume *3*

## Supplemental information

# Selective targeting of skin pathobionts and inflammation with topically applied lactobacilli

**Sarah Lebeer, Eline F.M. Oerlemans, Ingmar Claes, Tim Henkens, Lize Delanghe, Sander Wuyts, Irina Spacova, Marianne F.L. van den Broek, Ines Tuyaerts, Stijn Wittouck, Ilke De Boeck, Camille N. Allonsius, Filip Kiekens, and Julien Lambert**

# Supplementary information

## Supplementary Figures

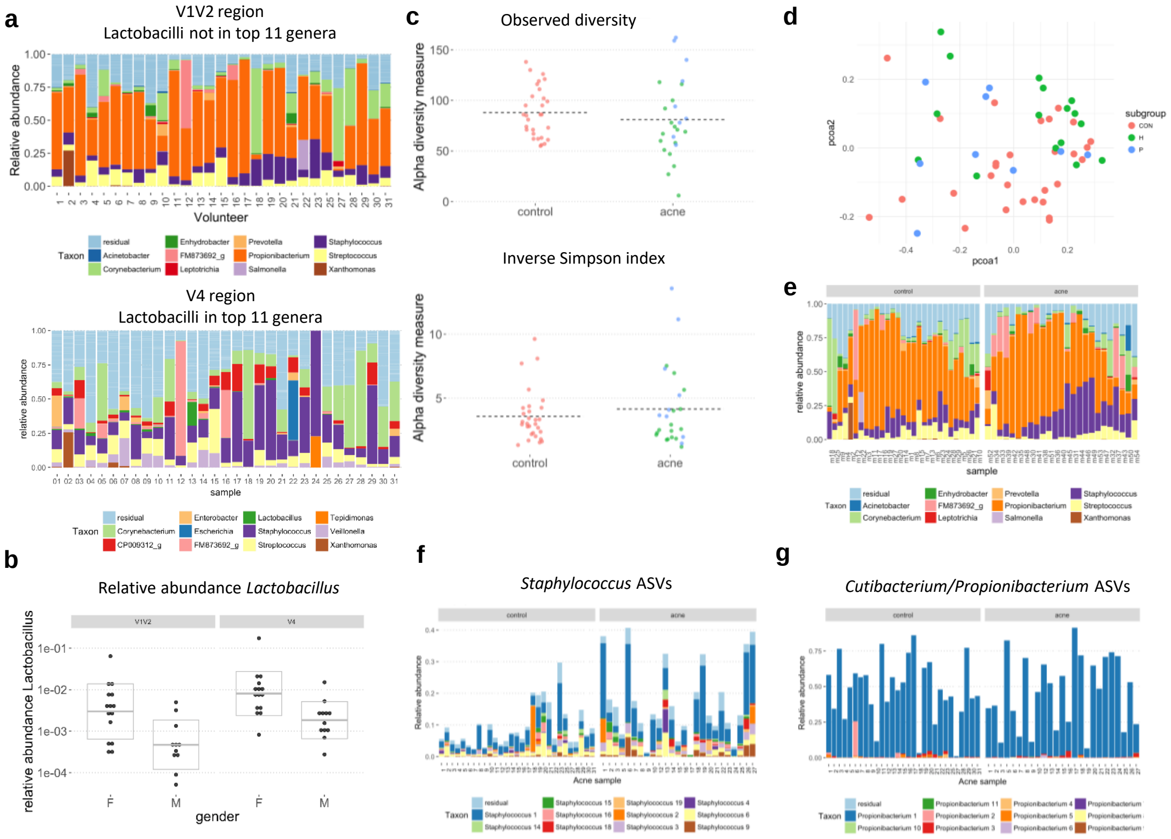


**Figure S1: Microbiome analysis of facial skin samples of control subjects (n= 30) (without acne symptoms) and comparison with subjects with mild-to moderate acne symptoms (n = 27) at baseline, without treatment.**

(a) Barplots showing taxonomy of the control group of volunteers (n= 30) without acne symptoms, as derived from V1V2 region (top) and V4 region (bottom) of the rRNA gene sequencing (b) Gender-specific relative abundance data for *taxa* of lactobacilli in the control group based on the different variable regions sequenced (V1V2 or V4)  (c) Alpha-diversity of samples as indicated by observed diversity (richness, left) and Inverse Simpson index (richness and evenness, right). (d) PCOA plot of baseline samples, based on Bray-Curtis distances between samples. Samples of the control group are shown in red, while acne samples of subjects that were included for the study treatment (n= 10) are indicated in blue and acne samples of subjects that were not included in the study treatment are shown in green (n= 17). (e) Barplot showing taxonomy (derived from V1V2 rRNA gene sequencing data) at genus level of the control and acne group. Samples are divided by group and clustered hierarchically to minimize Bray-Curtis distance. (f) Barplot showing relative abundances of 11 most abundant *Staphylococcus* ASVs in the control and acne group. (g) Barplot showing relative abundances of 11 most abundant *Propionibacterium/Cutibacterium* ASVs in the control and acne group. Related to Figure 1.

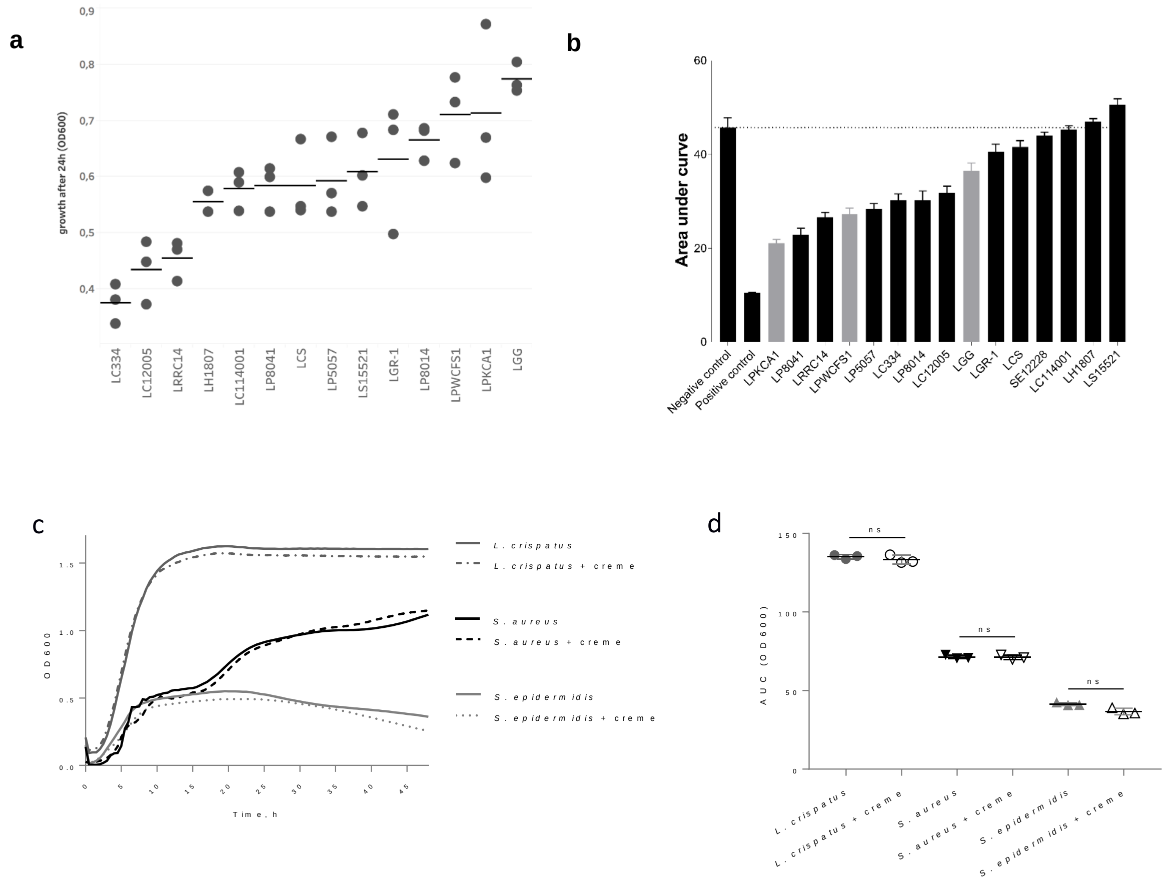


**Figure S2: *In vitro* selection of the lactobacilli strains**

(a) Optical densities of the cultures of lactobacilli tested after inoculation of MRS broth with 1% preculture and 24h incubation at 37°C. (b) Bacterial growth of *C. acnes* in the presence of Lactobacillus SCS. The antimicrobial activity of the SCS of the selected Lactobacillus strains against C. acnes is depicted as the area under the growth curve (OD600) and compared to the positive control (10 mg/mL Clindamycin, a common antibiotic used in acne). MRS at pH4, which is comparable to the pH of the SCS of lactobacilli, was used as a negative control. (B) Lactic acid bacteria used: *LPKCA1: L. pentosus KCA1, LP8041: L. plantarum ATCC 8041, LRRC14: L. reuteri RC14, LPWCFS1: L. plantarum WCFS1, LP5057: L. plantarum 5057, LC334: L. casei ATCC334, LP8014: L. pentosus ATCC 8014, LC12005: L. crispatus LMG 12005, LGG: L. rhamnosus GG, LGR-1: L. rhamnosus GR-1, LCS: L. casei Shirota, SE12228: S. epidermidis ATCC 12228, LC114001: L. casei DN-114001, LH1807: L. helveticus 1807, LS15521: L. sakei ATCC 15521.* (c) Impact of the ingredients of the topical cream without microcapsules on the growth capacity of the skin bacteria *Lactobacillus crispatus, C. acnes, Staphylococcus aureus* and *Staphylococcus epidermidis*. Growth curves of *L. crispatus*, *S. aureus* and *S. epidermidis* without (full lines) or with addition of topical cream ingredients at 1 mg/ml (dotted lines). Similar data were obtained for 100 mg/ml, but then we also observed more interference of the oily cream ingredients and the OD measurements. Data depicted as mean OD600 values plotted over the course of 48 hours; (d) Area under the curve (AUC) of *L. crispatus, S. aureus* and *S. epidermidis* growth curves with or without addition of topical cream. Individual AUC values depicted per tested well repetition with mean and SD per group, n = 3; ns: non-significant p-value (> 0.05) as calculated by the Mann-Whitney U-test**.** Related to Figure 2.

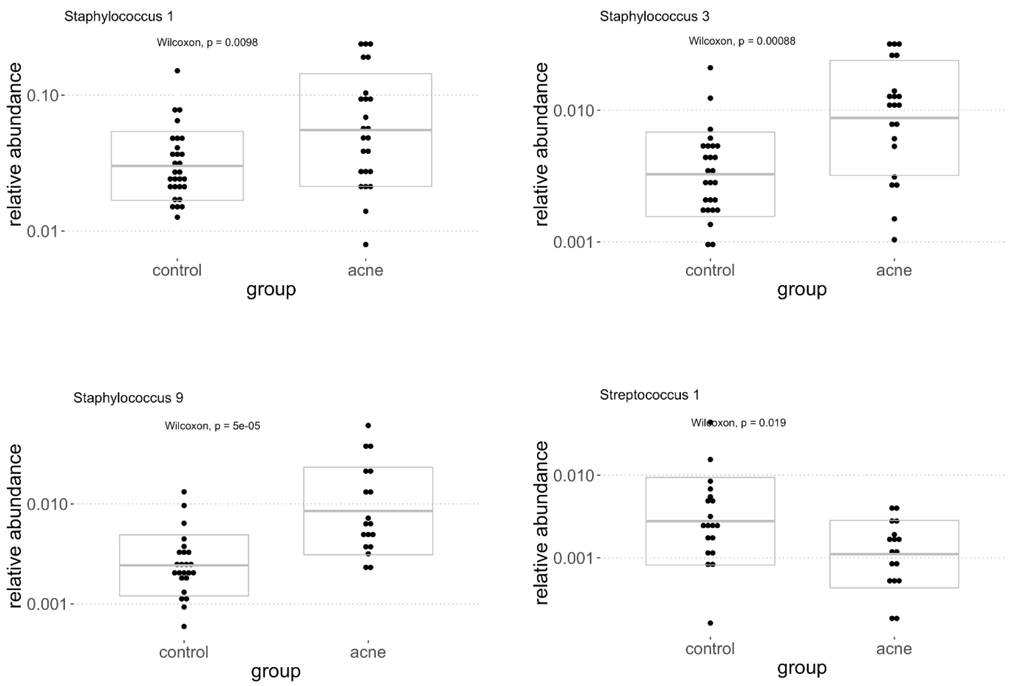


**Figure S3**: **Relative abundances of specific ASVs in the control group (n = 30) versus acne patients (n = 27)**

*Staphylococcus 1, Staphylococus 3,, Staphylococcus 9* (all annotated as *Staphylococcus epidermidis)* and *Streptococcus 1 (*annotated as *Streptococcus salivarius* according to EZ taxon) that showed significantly higher (A-C) or lower (D) relative abundances in the acne group as compared to the control group at baseline. To test for significant differences, a Kruskal-Wallis test was performed and p-values are indicated in the graphs. It is important to not here that although we suggest possible species-level classification here, this is actually not possible based on ASV analysis alone. These annotations should therefore be considered while keeping in mind the uncertainty for such classification. Related to Figure 4.

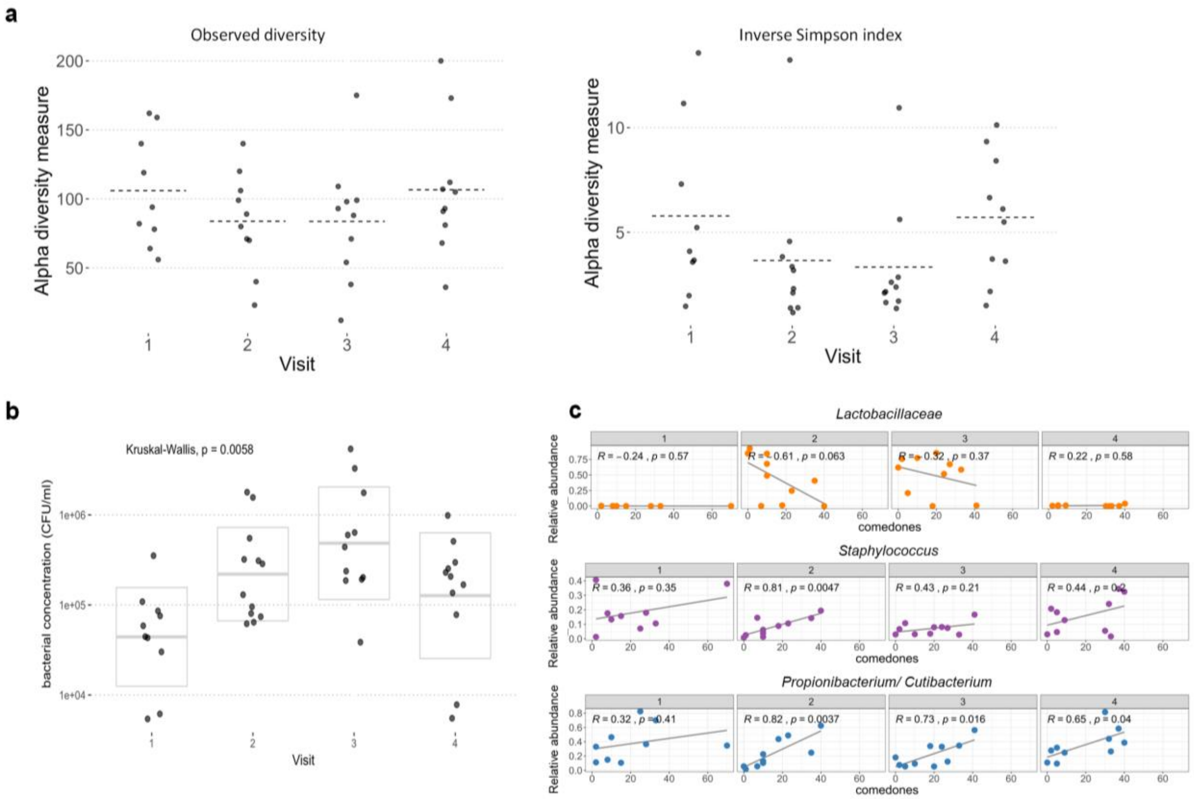


**Figure S4: Impact of the use of the probiotic cream on the skin microbiome.**

(a) Alpha-diversity as indicated by observed diversity (richness) and Inverse Simpson index (richness and evenness) of the samples throughout the study period, grouped by visit. (b) Estimated total bacterial concentration in the samples. Bacterial concentrations were estimated by quantitative PCR and a standard curve. (c) Correlation between bacterial taxa *Lactobacillaceae* (top), *Staphylococcus* (middle) and *Cutibacterium/ Propionibacterium* (bottom) and comedone count for each visit (x-axis)., Pearson's correlation coefficient is indicated. Related to Figure 4.

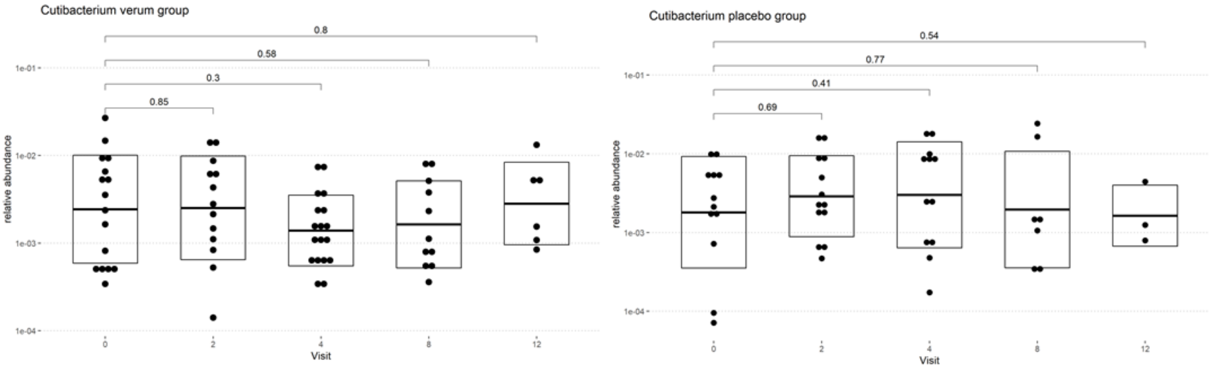


**Figure S5: Impact of the lactobacilli cream on the relative abundance of *Cutibacterium sp.* – DBPC study**

Relative abundances of *Cutibacterium sp.* in verum and placebo group during the study period, as found through 16S sequencing of the V4 hypervariable region. Related to Figure 5.

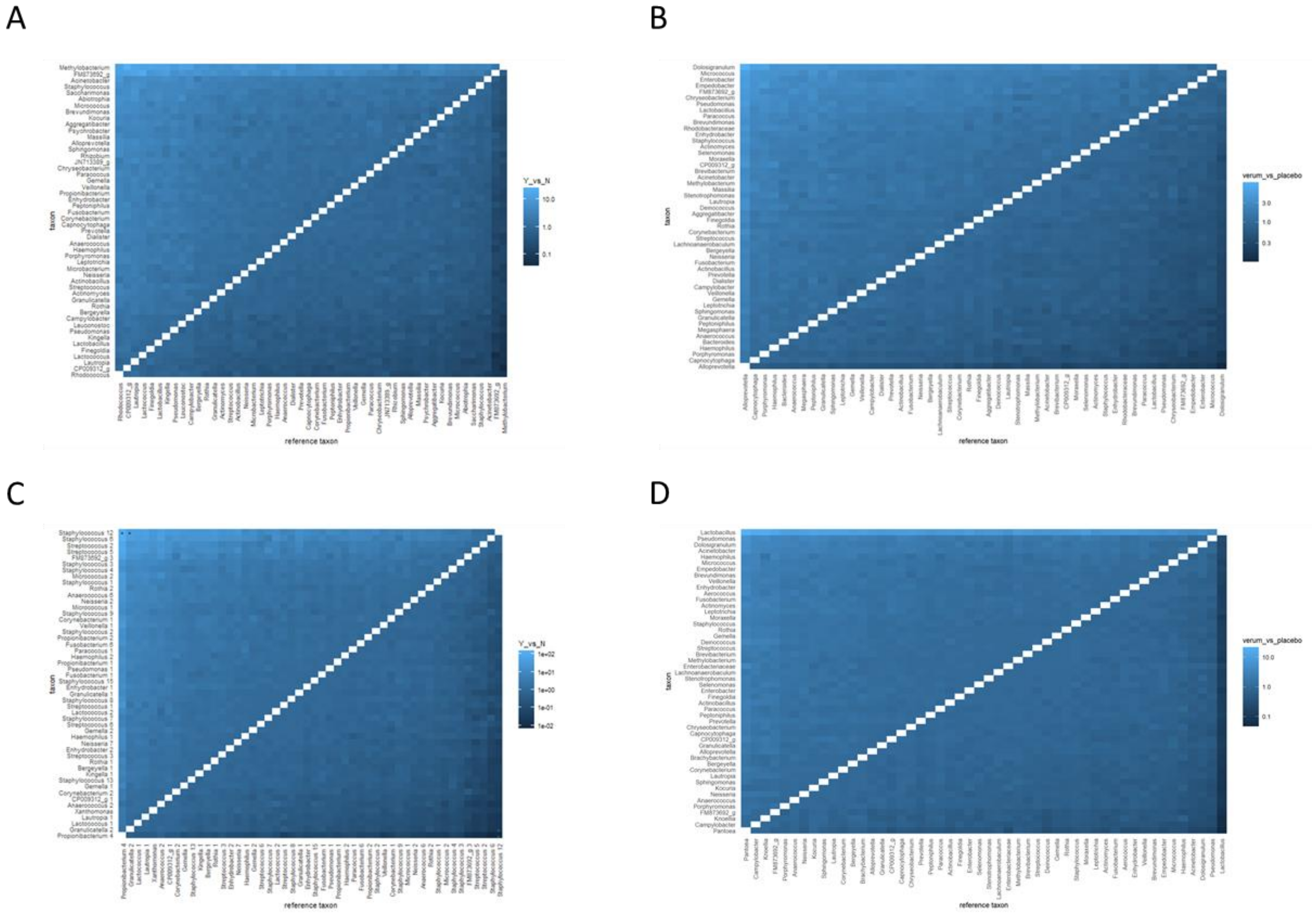


**Figure S6: Codifab plots showing differential relative abundance at genus level between two groups of participants for both POC and DBPC study.**

(a) Baseline of POC study: differential relative abundance for the most abundant genera between the healthy control group and acne patients at the time of inclusion. (b) Baseline of DBPC study: differential relative abundance for the most abundant genera between the verum group and placebo group at the time of inclusion. (c) POC study at visit 1: differential relative abundance for the most abundant genera between the healthy control group and acne patients after 4 weeks of treatment with a probiotic cream containing live lactobacilli. (d) DBPC study at visit 1: differential relative abundance for the most abundant genera between the verum and placebo group after 2 weeks of treatment with respectively the probiotic cream and placebo cream. + or – indicates significance in differential relative abundance for that specific genus. Related to Figure 6.

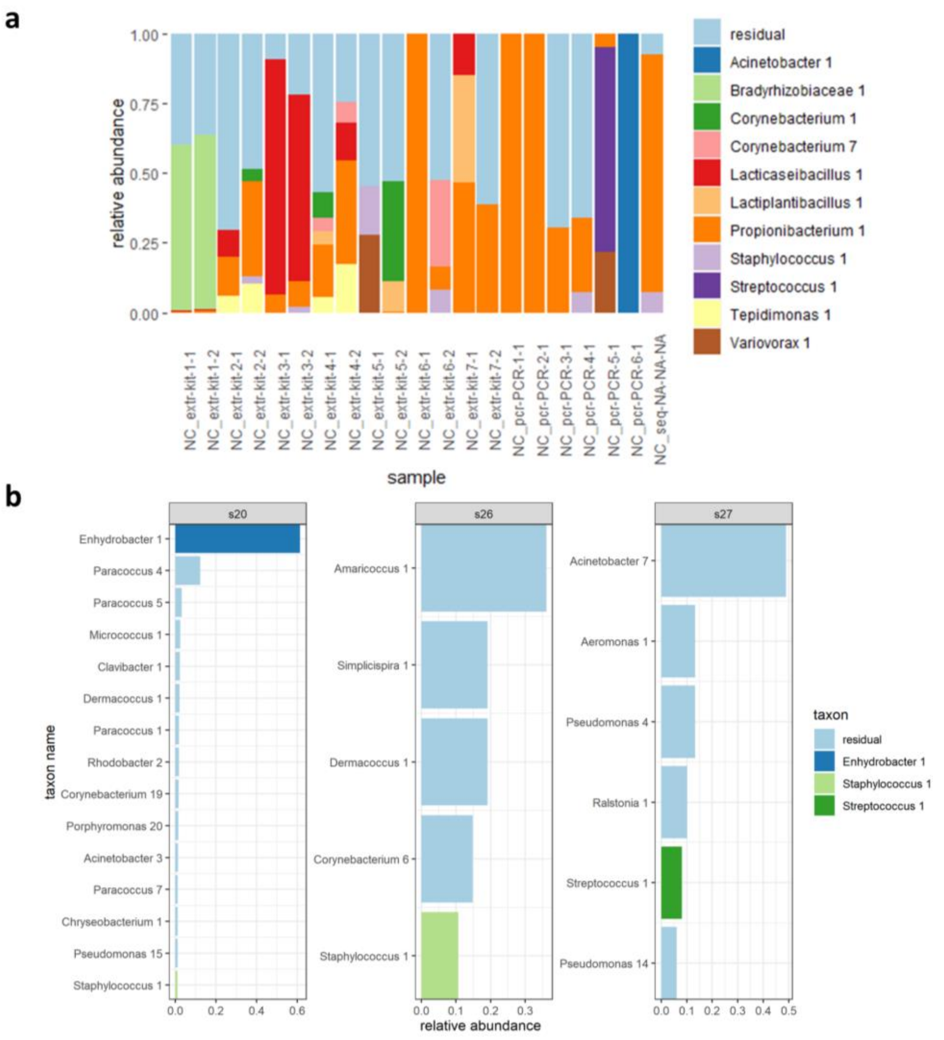


**Figure S7: Taxonomic classification of the negative controls for the DNA extraction kits and PCR for the pilot study (a) and the placebo-controlled study (b)**

(a) Negative controls for the pilot study included both kit controls and PCR negative controls. Read counts were generally relatively low in comparison to the samples (median controls: 431 reads; median samples: 44773.5 reads). (b) Negative controls for the placebo-study included only controls for PCR. 5 of 8 controls did not yield any reads after quality control. Related to STAR methods.

## Supplementary Tables

**Table S1: Bacterial strains used in this study.** Related to Figure 2.

| Species | Strain | Relevant genotype or description | Reference and/or source | Final selection for formulation in cream & POC study * |
|---|---|---|---|---|
| ***Lactocaseibacillus rhamnosus*** | GG | Single colony isolate of wild-type strain, isolated from human faeces | [19] | yes |
| ***Lactoplantibacillus plantarum*** | WCFS1 | Single colony isolate of *L. plantarum* WCFS1 | [20] | yes |
| ***Lactiplantibacillus pentosus*** | KCA1 | Single colony isolate from KCA1 (vaginal origin) | [21] | yes |
| ***Lacticaseibacillus casei*** | ATCC334 | Single colony isolate obtained from a stock culture of ATCC334 | ATCC | No, less active than WCFS1 & KCA1 against C. acnes & *S. aureus* |

| | | | | |
|---|---|---|---|---|
| ***Lacticaseibacillus casei**** <br><br> ***(LCS)*** | Shirota | Single colony isolate obtained from a commercially available fermented drink containing *L. casei* Shirota (Yakult®), confirmed by sequencing | Commercial probiotic product | No, less active than WCFS1 & KCA1 against *C. acnes* and & *S. aureus* |
| ***Lacticaseibacillus casei**** | DN-114001 | Single colony isolate obtained in our lab from a commercially available fermented drink (Actimel®) containing *L. casei* DN-114001, confirmed by sequencing | Commercial probiotic product | No, less active than WCFS1 & KCA1 against *C. acnes* and & *S. aureus* |
| ***Lactobacillus crispatus*** | LMG12005 | | BCCM/LMG | No, less robust & less growth capacity than other lactobacilli tested |
| ***Lactobacillus helveticus*** | 1807 | single colony isolate | Commercial probiotic product | No, less active than WCFS1 & KCA1 against *C. acnes* and & *S. aureus* |
| ***Lacticaseibacillus paracasei*** | LMG12586 | Single colony isolate obtained from a stock culture of LMG12586 | BCCM/LMG | No, less active than WCFS1 & KCA1 against *C. acnes* and & *S. aureus* |
| ***Lactiplantibacillus pentosus*** | ATCC8041 | | BCCM/LMG | No, less active than WCFS1 & KCA1 against *C. acnes* and & *S. aureus* |
| ***Lactiplantibacillusplantarum*** | ATCC8014 | Single colony isolate from *L. plantarum* ATCC8014 or LMG1284 | BCCM/LMG | No, less active than WCFS1 & KCA1 against *C. acnes* and & *S. aureus* |
| ***Lactiplantibacillusplantarum*** | 5057 | Single colony isolate of *L. plantarum* 5057 | [49] | No, presence of antibiotic resistance genes (Tetracycline) |
| ***Limosilactobacillus reuteri*** | RC14 | Single colony isolate from a commercially available probiotic supplement containing *L. reuteri* RC14, confirmed by sequencing | ATCC | No, because slight induction of inflammation in primary skin cells |
| ***Lacticaseibacillus rhamnosus*** | GR-1 | Single colony isolate obtained from a commercially available probiotic supplement containing *L. rhamnosus* GR-1 (urethra origin) | [28] | No, less active than WCFS1 & KCA1 against *C. acnes* and & *S. aureus* |
| ***Latilactobacillus sakei*** | ATCC15521 | | BCCM/LMG | Not active against *C. acnes* and & *S. aureus* |
| ***Staphylococcus epidermidis*** | ATCC12228 | | BCCM/LMG | No GRAS/QPS status |

* Main reason for non-selection is indicated, but also other aspects such as availability and freedom-to-operate, growth capacity, and level of scientific documentation in the literature were considered.

**Table S2: Clinical data of the skin patch test results for the O/W *Lactobacillus* cream.** Related to Figure 3.

| Vol ID | Volunteer code | Gender | Age | Erythema | Dryness | Edema | Tot readings 48hrs |
|---|---|---|---|---|---|---|---|
| 1 | 1176 | F | 55 | 0 | 0 | 0 | 0 |
| 2 | 1182 | F | 25 | 0 | 0 | 0 | 0 |
| 3 | 1191 | F | 24 | 0 | 0 | 0 | 0 |
| 4 | 1202 | F | 54 | 0 | 0 | 0 | 0 |
| 5 | 1211 | M | 59 | 0 | 0 | 0 | 0 |
| 6 | 1258 | M | 29 | 0 | 0 | 0 | 0 |
| 7 | 1263 | F | 64 | 0 | 0 | 0 | 0 |

| | | | | | | | |
|---|---|---|---|---|---|---|---|
| 8 | 1335 | M | 47 | 0 | 0 | 0 | 0 |
| 9 | 1364 | F | 50 | 0 | 0 | 0 | 0 |
| 10 | 1458 | F | 22 | 0 | 0 | 0 | 0 |
| 11 | 1509 | F | 30 | 0 | 0 | 0 | 0 |
| 12 | 1549 | F | 23 | 0 | 0 | 0 | 0 |
| 13 | 1623 | F | 24 | 0 | 0 | 0 | 0 |
| 14 | 1700 | F | 44 | 0 | 0 | 0 | 0 |
| 15 | 1701 | M | 41 | 0 | 0 | 0 | 0 |
| 16 | 1702 | M | 24 | 0 | 0 | 0 | 0 |
| 17 | 1703 | M | 25 | 0 | 0 | 0 | 0 |
| 18 | 1704 | M | 24 | 0 | 0 | 0 | 0 |
| 19 | 1705 | F | 31 | 0 | 0 | 0 | 0 |
| 20 | 1706 | F | 20 | 0 | 0 | 0 | 0 |

**Table S3: Pilot trial's patients characteristics and clinical evaluation after 4 and 8 weeks of treatment compared to baseline.** Related to Figure 5.

| Patient ID | Adverse effects | Inflammatory lesion count (% decrease) | | Comedonal count (% decrease) | | Remarks |
|---|---|---|---|---|---|---|
| | | 4 weeks | 8 weeks | 4 weeks | 8 weeks | |
| 1 | no | 12.0 | 40.0 | 50.0 | 71.4 | Patient saw first improvements after 1st week. Global improvement at V2. Further improvement at V3 but with fluctuation. Symptoms reappearing 3 days after stop of treatment |
| 2 | no | 26.9 | 65.4 | 0.0 | -2.5 | Slight reduction in comedones and papules at V2 Improvement continued at V3 but with fluctuation. At visit 4 still improvement compared to baseline. |
| 3 | no | 80.0 | 100.0 | 35.7 | 35.7 | Mostly comedonal acne. Patient was very positive with improvement observed after 3 days. Reduction in comedones at V2, V3 and V4. |
| 4 | no | 53.8 | 15.4 | 30.3 | 27.3 | At V2 still multiple comedones on forehead but improvement on cheeks (no more pustules) and chin (reduction in pustules). At V3 continued improvement. Only some inflammatory lesions on forehead but only comedones on chin, nose and cheeks. At V4, after stop of treatment, quick relapse of symptoms. |
| 5 | no | 66.7 | 48.1 | 60.0 | -32.0 | Patient noticed an increase in comedones in first 2 weeks but global improvement at V2. Patient follow-up less consistent between V2 and V3, which resulted in reappearing of symptoms. At V4, after stop of treatment, relapse of symptoms. |
| 6 | no | 80.0 | 95.0 | 50.0 | 0.0 | Only slight improvement at V2. At V3 patient responded that the improvement was very significant after 6 weeks of treatment. At V3: almost perfect. At V4: Slight relapse after stop of treatment. |

| | | | | | | |
|---|---|---|---|---|---|---|
| 7 | no | 70.8 | 45.8 | -25.0 | -237.5 | Slight improvement depending on skin location at V2 and V3. At V4, after stop of treatment quick relapse of symptoms. |
| 8 | no | 14.3 | 23.8 | 100.0 | 100.0 | Improvement at V2 (inflammatory lesions from 22 -> 18). At V3 even more improvement, with reduction in inflammatory lesion (18 -> 11). First days after stop of the treatment, relapse but quickly stabilized. |
| 9 | no | 77.8 | 38.9 | 0.0 | 0.0 | At V2, global improvement of symptoms. Inflammatory lesions drop from 18 -> 4. At V3, some relapse compared to V2. Inflammatory lesions – 4 -> 11. At V4, only 7 inflammatory lesions |
| 10 | no | 20.0 | 20.0 | 53.3 | 66.7 | At V2, global improvement on acne symptoms, both inflammatory and comedones. (infl: 5 -> 4). At V3, improvement continued but still 4 papels but no more comedones on forehead. At V4, clear improvement of acne symptoms compared to start. No inflammatory lesions. |

**Table S4: Dermatological Assessment of Tolerance based on clinical signs, performed by a dermatologist at all visits.** Related to Figure 7.

| Verum (lactobacilli-supplemented cream) | | | | | | |
|---|---|---|---|---|---|---|
| **Attribute** | **Time-point** | **0-None** | **1-Very mild** | **2-Mild** | **3-Moderate** | **4-Severe** |
| Erythema | Tbaseline | 100% (36) | 0% (0) | 0% (0) | 0% (0) | 0% (0) |
| | T2w | 100% (36) | 0% (0) | 0% (0) | 0% (0) | 0% (0) |
| | T4w | 97.2% (35) | 0% (0) | 2.8% (1) | 0% (0) | 0% (0) |
| | T8w | 100% (36) | 0% (0) | 0% (0) | 0% (0) | 0% (0) |
| | T12w | 100% (36) | 0% (0) | 0% (0) | 0% (0) | 0% (0) |
| Edema | Tbaseline | 100% (36) | 0% (0) | 0% (0) | 0% (0) | 0% (0) |
| | T2w | 100% (36) | 0% (0) | 0% (0) | 0% (0) | 0% (0) |
| | T4w | 100% (36) | 0% (0) | 0% (0) | 0% (0) | 0% (0) |
| | T8w | 100% (36) | 0% (0) | 0% (0) | 0% (0) | 0% (0) |
| | T12w | 100% (36) | 0% (0) | 0% (0) | 0% (0) | 0% (0) |
| Scaling | Tbaseline | 100% (36) | 0% (0) | 0% (0) | 0% (0) | 0% (0) |
| | T2w | 100% (36) | 0% (0) | 0% (0) | 0% (0) | 0% (0) |
| | T4w | 97.2% (35) | 2.8% (1) | 0% (0) | 0% (0) | 0% (0) |
| | T8w | 97.2% (35) | 2.8% (1) | 0% (0) | 0% (0) | 0% (0) |
| | T12w | 100% (36) | 0% (0) | 0% (0) | 0% (0) | 0% (0) |
| Dryness | Tbaseline | 100% (36) | 0% (0) | 0% (0) | 0% (0) | 0% (0) |
| | T2w | 100% (36) | 0% (0) | 0% (0) | 0% (0) | 0% (0) |
| | T4w | 100% (36) | 0% (0) | 0% (0) | 0% (0) | 0% (0) |
| | T8w | 100% (36) | 0% (0) | 0% (0) | 0% (0) | 0% (0) |

| | | | | | | |
|---|---|---|---|---|---|---|
| | T12w | 100% (36) | 0% (0) | 0% (0) | 0% (0) | 0% (0) |
| Others | Tbaseline | 100% (36) | 0% (0) | 0% (0) | 0% (0) | 0% (0) |
| | T2w | 100% (36) | 0% (0) | 0% (0) | 0% (0) | 0% (0) |
| | T4w | 100% (36) | 0% (0) | 0% (0) | 0% (0) | 0% (0) |
| | T8w | 100% (36) | 0% (0) | 0% (0) | 0% (0) | 0% (0) |
| | T12w | 100% (36) | 0% (0) | 0% (0) | 0% (0) | 0% (0) |
| **Placebo cream** | | | | | | |
| **Attribute** | **Time-point** | **0-None** | **1-Very mild** | **2-Mild** | **3-Moderate** | **4-Severe** |
| Erythema | Tbaseline | 100% (31) | 0% (0) | 0% (0) | 0% (0) | 0% (0) |
| | T2w | 100% (31) | 0% (0) | 0% (0) | 0% (0) | 0% (0) |
| | T4w | 100% (31) | 0% (0) | 0% (0) | 0% (0) | 0% (0) |
| | T8w | 100% (31) | 0% (0) | 0% (0) | 0% (0) | 0% (0) |
| | T12w | 100% (31) | 0% (0) | 0% (0) | 0% (0) | 0% (0) |
| Edema | Tbaseline | 100% (31) | 0% (0) | 0% (0) | 0% (0) | 0% (0) |
| | T2w | 100% (31) | 0% (0) | 0% (0) | 0% (0) | 0% (0) |
| | T4w | 100% (31) | 0% (0) | 0% (0) | 0% (0) | 0% (0) |
| | T8w | 100% (31) | 0% (0) | 0% (0) | 0% (0) | 0% (0) |
| | T12w | 100% (31) | 0% (0) | 0% (0) | 0% (0) | 0% (0) |
| Scaling | Tbaseline | 100% (31) | 0% (0) | 0% (0) | 0% (0) | 0% (0) |
| | T2w | 100% (31) | 0% (0) | 0% (0) | 0% (0) | 0% (0) |
| | T4w | 100% (31) | 0% (0) | 0% (0) | 0% (0) | 0% (0) |
| | T8w | 96.8% (30) | 3.2% (1) | 0% (0) | 0% (0) | 0% (0) |
| | T12w | 100% (31) | 0% (0) | 0% (0) | 0% (0) | 0% (0) |
| Dryness | Tbaseline | 100% (31) | 0% (0) | 0% (0) | 0% (0) | 0% (0) |
| | T2w | 100% (31) | 0% (0) | 0% (0) | 0% (0) | 0% (0) |
| | T4w | 100% (31) | 0% (0) | 0% (0) | 0% (0) | 0% (0) |
| | T8w | 100% (31) | 0% (0) | 0% (0) | 0% (0) | 0% (0) |
| | T12w | 100% (31) | 0% (0) | 0% (0) | 0% (0) | 0% (0) |
| Others | Tbaseline | 100% (31) | 0% (0) | 0% (0) | 0% (0) | 0% (0) |
| | T2w | 100% (31) | 0% (0) | 0% (0) | 0% (0) | 0% (0) |
| | T4w | 100% (31) | 0% (0) | 0% (0) | 0% (0) | 0% (0) |
| | T8w | 100% (31) | 0% (0) | 0% (0) | 0% (0) | 0% (0) |
| | T12w | 100% (31) | 0% (0) | 0% (0) | 0% (0) | 0% (0) |

**Table S5: Dermatological Assessment of Tolerance based on subjective signs.** Related to Figure 7.

| Verum (lactobacilli-supplemented cream) | | | | | | |
|---|---|---|---|---|---|---|
| **Attribute** | **Time-point** | **0-None** | **1-Very mild** | **2-Mild** | **3-Moderate** | **4-Severe** |
| Itching | Tbaseline | 100% (36) | 0% (0) | 0% (0) | 0% (0) | 0% (0) |
| | T2w | 100% (36) | 0% (0) | 0% (0) | 0% (0) | 0% (0) |
| | T4w | 100% (36) | 0% (0) | 0% (0) | 0% (0) | 0% (0) |
| | T8w | 100% (36) | 0% (0) | 0% (0) | 0% (0) | 0% (0) |
| | T12w | 100% (36) | 0% (0) | 0% (0) | 0% (0) | 0% (0) |
| Burning | Tbaseline | 100% (36) | 0% (0) | 0% (0) | 0% (0) | 0% (0) |
| | T2w | 100% (36) | 0% (0) | 0% (0) | 0% (0) | 0% (0) |
| | T4w | 100% (36) | 0% (0) | 0% (0) | 0% (0) | 0% (0) |
| | T8w | 100% (36) | 0% (0) | 0% (0) | 0% (0) | 0% (0) |
| | T12w | 100% (36) | 0% (0) | 0% (0) | 0% (0) | 0% (0) |
| Heating sensation | Tbaseline | 100% (36) | 0% (0) | 0% (0) | 0% (0) | 0% (0) |
| | T2w | 100% (36) | 0% (0) | 0% (0) | 0% (0) | 0% (0) |
| | T4w | 100% (36) | 0% (0) | 0% (0) | 0% (0) | 0% (0) |
| | T8w | 100% (36) | 0% (0) | 0% (0) | 0% (0) | 0% (0) |
| | T12w | 100% (36) | 0% (0) | 0% (0) | 0% (0) | 0% (0) |
| Stinging | Tbaseline | 100% (36) | 0% (0) | 0% (0) | 0% (0) | 0% (0) |
| | T2w | 100% (36) | 0% (0) | 0% (0) | 0% (0) | 0% (0) |
| | T4w | 100% (36) | 0% (0) | 0% (0) | 0% (0) | 0% (0) |
| | T8w | 100% (36) | 0% (0) | 0% (0) | 0% (0) | 0% (0) |
| | T12w | 100% (36) | 0% (0) | 0% (0) | 0% (0) | 0% (0) |
| Tingling | Tbaseline | 100% (36) | 0% (0) | 0% (0) | 0% (0) | 0% (0) |
| | T2w | 100% (36) | 0% (0) | 0% (0) | 0% (0) | 0% (0) |
| | T4w | 100% (36) | 0% (0) | 0% (0) | 0% (0) | 0% (0) |
| | T8w | 100% (36) | 0% (0) | 0% (0) | 0% (0) | 0% (0) |
| | T12w | 100% (36) | 0% (0) | 0% (0) | 0% (0) | 0% (0) |
| Tightening | Tbaseline | 100% (36) | 0% (0) | 0% (0) | 0% (0) | 0% (0) |
| | T2w | 100% (36) | 0% (0) | 0% (0) | 0% (0) | 0% (0) |
| | T4w | 100% (36) | 0% (0) | 0% (0) | 0% (0) | 0% (0) |
| | T8w | 100% (36) | 0% (0) | 0% (0) | 0% (0) | 0% (0) |
| | T12w | 100% (36) | 0% (0) | 0% (0) | 0% (0) | 0% (0) |
| Others | Tbaseline | 100% (36) | 0% (0) | 0% (0) | 0% (0) | 0% (0) |
| | T2w | 100% (36) | 0% (0) | 0% (0) | 0% (0) | 0% (0) |
| | T4w | 100% (36) | 0% (0) | 0% (0) | 0% (0) | 0% (0) |
| | T8w | 100% (36) | 0% (0) | 0% (0) | 0% (0) | 0% (0) |
| | T12w | 100% (36) | 0% (0) | 0% (0) | 0% (0) | 0% (0) |
| **Placebo cream** | | | | | | |
| **Attribute** | **Time-point** | **0-None** | **1-Very mild** | **2-Mild** | **3-Moderate** | **4-Severe** |

| | | | | | | |
|---|---|---|---|---|---|---|
| Itching | Tbaseline | 100% (31) | 0% (0) | 0% (0) | 0% (0) | 0% (0) |
| | T2w | 100% (31) | 0% (0) | 0% (0) | 0% (0) | 0% (0) |
| | T4w | 100% (31) | 0% (0) | 0% (0) | 0% (0) | 0% (0) |
| | T8w | 100% (31) | 0% (0) | 0% (0) | 0% (0) | 0% (0) |
| | T12w | 96.8% (30) | 3.2% (1) | 0% (0) | 0% (0) | 0% (0) |
| Burning | Tbaseline | 100% (31) | 0% (0) | 0% (0) | 0% (0) | 0% (0) |
| | T2w | 100% (31) | 0% (0) | 0% (0) | 0% (0) | 0% (0) |
| | T4w | 100% (31) | 0% (0) | 0% (0) | 0% (0) | 0% (0) |
| | T8w | 100% (31) | 0% (0) | 0% (0) | 0% (0) | 0% (0) |
| | T12w | 100% (31) | 0% (0) | 0% (0) | 0% (0) | 0% (0) |
| Heating sensation | Tbaseline | 100% (31) | 0% (0) | 0% (0) | 0% (0) | 0% (0) |
| | T2w | 100% (31) | 0% (0) | 0% (0) | 0% (0) | 0% (0) |
| | T4w | 100% (31) | 0% (0) | 0% (0) | 0% (0) | 0% (0) |
| | T8w | 100% (31) | 0% (0) | 0% (0) | 0% (0) | 0% (0) |
| | T12w | 100% (31) | 0% (0) | 0% (0) | 0% (0) | 0% (0) |
| Stinging | Tbaseline | 100% (31) | 0% (0) | 0% (0) | 0% (0) | 0% (0) |
| | T2w | 100% (31) | 0% (0) | 0% (0) | 0% (0) | 0% (0) |
| | T4w | 100% (31) | 0% (0) | 0% (0) | 0% (0) | 0% (0) |
| | T8w | 100% (31) | 0% (0) | 0% (0) | 0% (0) | 0% (0) |
| | T12w | 100% (31) | 0% (0) | 0% (0) | 0% (0) | 0% (0) |
| Tingling | Tbaseline | 100% (31) | 0% (0) | 0% (0) | 0% (0) | 0% (0) |
| | T2w | 100% (31) | 0% (0) | 0% (0) | 0% (0) | 0% (0) |
| | T4w | 100% (31) | 0% (0) | 0% (0) | 0% (0) | 0% (0) |
| | T8w | 100% (31) | 0% (0) | 0% (0) | 0% (0) | 0% (0) |
| | T12w | 100% (31) | 0% (0) | 0% (0) | 0% (0) | 0% (0) |
| Tightening | Tbaseline | 100% (31) | 0% (0) | 0% (0) | 0% (0) | 0% (0) |
| | T2w | 100% (31) | 0% (0) | 0% (0) | 0% (0) | 0% (0) |
| | T4w | 100% (31) | 0% (0) | 0% (0) | 0% (0) | 0% (0) |
| | T8w | 100% (31) | 0% (0) | 0% (0) | 0% (0) | 0% (0) |
| | T12w | 100% (31) | 0% (0) | 0% (0) | 0% (0) | 0% (0) |
| Others | Tbaseline | 100% (31) | 0% (0) | 0% (0) | 0% (0) | 0% (0) |
| | T2w | 100% (31) | 0% (0) | 0% (0) | 0% (0) | 0% (0) |
| | T4w | 100% (31) | 0% (0) | 0% (0) | 0% (0) | 0% (0) |
| | T8w | 100% (31) | 0% (0) | 0% (0) | 0% (0) | 0% (0) |
| | T12w | 100% (31) | 0% (0) | 0% (0) | 0% (0) | 0% (0) |

## Supplementary Methods

**Methods S1: Registration of placebo-controlled study at ClinicalTrails.gov with additional details of the study.** Related to STAR methods. (see next page)

COVID-19 is an emerging, rapidly evolving situation.
Get the latest public health information from CDC: https://www.coronavirus.gov.
Get the latest research information from NIH: https://www.nih.gov/coronavirus.



# Skin Acceptance and Efficacy Assessment of a Topical Product in Acne Treatment When Compared to a Placebo.

The safety and scientific validity of this study is the responsibility of the study sponsor and investigators. Listing a study does not mean it has been evaluated by the U.S. Federal Government. Read our disclaimer for details.

ClinicalTrials.gov Identifier: NCT04216160

Recruitment Status : Active, not recruiting
First Posted : January 2, 2020
Last Update Posted : January 2, 2020

**Sponsor:**
**YUN** NV

**Collaborator:**
**Allergisa** Pesquisa Dermato-Cosmetica LTDA

**Information provided by (Responsible Party):**
YUN NV



## Study Description

Go to

Brief Summary:
In this study the topical use of cream with live probiotic bacteria was evaluated for its efficacy in reducing acne symptoms and its effect on the skin microbiota on patients with acne vulgaris. Patients with mild to moderate acne

used the probiotic cream for 8 weeks and clinical evaluation and microbiological sampling was done at start, 2, 4, 8 and 12 weeks (after 4 weeks without use of the product). Next-Generation Sequencing is used to analyze the skin microbiota of the patients.

| Condition or disease | Intervention/treatment | Phase |
|---|---|---|
| **Acne Vulgaris** | Other: ACN cream (**YUN**)<br>Other: Placebo cream (**YUN**) | Not Applicable |

Detailed Description:
Probiotics are live micro-organisms which when administered in adequate amounts can exert a health benefit on the host. This health-promoting effects have been extensively studied in the gastrointestinal niche but it becomes more and more clear that other niches are also interesting for the potential of probiotics. Recent breakthroughs in 'next generation sequencing' (NGS) technologies are making it now possible to map the microbiota after DNA extraction, which is very interesting for bacteria that are not or difficult to cultivate. The research into the microbiota of the skin with such new NGS technologies shows that there is also an equilibrium in the skin composition of the microbiota and that there is a disturbance of the skin microbiota in acne. Acne vulgaris is known as a multifactorial condition, both hormonal triggers and environmental factors play a role. However, it is also known that Cutibacterium acnes and Staphylococcus spp. play an important role in the inflammation of the sebaceous gland follicles. Therefore, probiotic strains with antipathogenic activity against these bacteria and suitable for application to the skin are potentially able to restore the balance of the skin microbiota and reduce acne symptoms. The main objective of this study was to verify the skin acceptance and efficacy of the cream with live probiotic bacteria for acne treatment in comparison with a placebo. More specifically to evaluate the effect of the 'live' Lactobacillus species as 'active ingredient' in relation to acne symptoms and skin microbiome modulation.

## Study Design

Go to

**Study Type :**
Interventional (Clinical Trial)

**Actual Enrollment :**
80 participants

**Allocation:**
Randomized

**Intervention Model:**
Parallel Assignment

**Masking:**
Quadruple (Participant, Care Provider, Investigator, Outcomes Assessor)

**Primary Purpose:**
Treatment

**Official Title:**
Skin Acceptance and Efficacy Assessment of a Topical Product in **Acne** Treatment When Compared to a Placebo, Under Normal Use Conditions.

**Actual Study Start Date :**
May 5, 2019

**Actual Primary Completion Date :**
November 28, 2019

**Estimated Study Completion Date :**
December 31, 2020

**Resource links provided by the National Library of Medicine**

NIH NLM

MedlinePlus related topics: Acne

U.S. FDA Resources

## Arms and Interventions

Go to

| Arm | Intervention/treatment |
|---|---|
| Experimental: Verum<br>Patients with mild to moderate acne using ACN Cream | Other: ACN cream (**YUN**)<br>Application of the facial cream ACN (**YUN**) twice a day, for 8 weeks +/- 2 days. Assessment before product use (T0), after 2, 4 and 8 weeks +/- 2 days of product use (T2w, T4w and T8w, respectively) and after 4 weeks +/- 2 days without product use (T12w).<br>Other Name: Topical cream with live probiotic bacteria |
| Experimental: Placebo<br>Patients with mild to moderate acne using the placebo cream | Other: Placebo cream (**YUN**)<br>Application of the facial placebo cream twice a day, for 8 weeks +/- 2 days. Assessment before product use (T0), after 2, 4 and 8 weeks +/- 2 days of product use (T2w, T4w and T8w, respectively) and after 4 weeks +/- 2 days without product use (T12w). |

## Outcome Measures

Go to

Primary Outcome Measures :

1. Change of inflammatory lesions compared to placebo. [ Time Frame: baseline, 2, 4 and 8 weeks of product use and 4 weeks without product use (Total 12 weeks). ]

   The subjects were assessed by a trained technician in order to perform the acne lesions counting.

2. Change of inflammatory lesions compared to baseline. [ Time Frame: baseline, 2, 4 and 8 weeks of product use and 4 weeks without product use (Total 12 weeks). ]

   The subjects were assessed by a trained technician in order to perform the acne lesions counting.

3. Overall tolerance of the treatment [ Time Frame: Baseline to week 12 ]

   Dermatological Assessment of Tolerance (DAT). On all visits, the dermatologist performed an assessment of the study subjects' faces according to a 5-point scale. The physician recorded in the subject's case report form possible discomforts sensation informed.

## Eligibility Criteria

Go to

**Information from the National Library of Medicine**

NIH NLM

*Choosing to participate in a study is an important personal decision. Talk with your doctor and family members or friends about deciding to join a study. To learn more about this study, you or your doctor may contact the study research staff using the contacts provided below. For general information, Learn About Clinical Studies.*

**Ages Eligible for Study:**
12 Years to 35 Years (Child, Adult)

**Sexes Eligible for Study:**
All

**Accepts Healthy Volunteers:**
No

**Criteria**

Inclusion Criteria:

- Healthy skin in the test areas;
- Subjects willing and capable to follow the study rules and a fixed schedule;
- Ability of giving consent for participation in the study;
- Subjects with good health in general and good mental condition;

- Subjects who present at least 10 inflammatory lesions;
- Oily skin on the face (minimum sebumetry value 100µg/cm² on frontal area (mean: 3 measurements)).

Exclusion Criteria:

- Pregnancy or breastfeeding;
- Subjects who present severe acne;
- Subjects who present more than two nodular lesions;
- Subjects who changed their oral contraception method up to three months before the study beginning;
- Subjects who did acne hormonal treatment less than 6 months before the study;
- Subjects who did oral isotretinoïne treatment less than 1 month before the study;
- Subjects who did topical acne treatment less than 90 months before the study;
- Subjects who did aesthetic treatment less than 6 months before the study (like laser and peeling);
- Subjects who did treatment with antibiotics within the last 4 months;
- Simultaneous participation in different studies from external research institutes on the same test sites;
- Inadequate language proficiency (spoken and written);
- Participate in the study under the influence of alcohol and/or drugs as well as addiction;
- Severe psychological disease or intellectual disability of understanding the study;
- Severe disease (heart/circulatory/liver, kidney and lungs disease, severe diabetes mellitus) or chronic infections (hepatitis, HIV);
- Immune insufficiency;
- Current use of the following topical or systemic medications: corticosteroids, immunosuppressive and anti-histaminic drugs;
- Skin diseases: vitiligo, psoriasis, atopic dermatitis;
- Confirmed allergies to cosmetic components or previous responses of intolerance after the application of cosmetic products of the same category of the investigational products;
- Other diseases or medications that might directly interfere in the study or put the subject's health under risk.

## Contacts and Locations

Go to

**Information from the National Library of Medicine**

NIH NLM

*To learn more about this study, you or your doctor may contact the study research staff using the contact information provided by the sponsor.*

*Please refer to this study by its ClinicalTrials.gov identifier (NCT number):* ***NCT04216160***

### Locations

#### Brazil

**Allergisa** Pesquisa Dermato-Cosmética Ltda
Campinas, SP, **Brazil**, 13084-791

### Sponsors and Collaborators

**YUN** NV

**Allergisa** Pesquisa Dermato-Cosmetica LTDA

### Investigators

Principal Investigator: Mariane Mosca **Allergisa** Pesquisa Dermato-Cosmética Ltda

## More Information

Go to

**Responsible Party:**
YUN NV

**ClinicalTrials.gov Identifier:**
NCT04216160 History of Changes

**Other Study ID Numbers:**
074785-01/02-04-19-PRV03

**First Posted:**
January 2, 2020 Key Record Dates

**Last Update Posted:**
January 2, 2020

**Last Verified:**
December 2019

**Individual Participant Data (IPD) Sharing Statement:**

**Plan to Share IPD:**
No

**Studies a U.S. FDA-regulated Drug Product:**
No

**Studies a U.S. FDA-regulated Device Product:**
No

**Additional relevant MeSH terms:**

**Acne Vulgaris**

Acneiform Eruptions

Skin Diseases

Sebaceous Gland Diseases

**Methods S2: Final report of placebo-controlled study from Allergisa, with additional details on the study design and population.** Related to STAR methods. (see next page)

# SKIN ACCEPTANCE AND EFFICACY ASSESSMENT OF A TOPICAL PRODUCT IN ACNE TREATMENT WHEN COMPARED TO A PLACEBO, UNDER NORMAL USE CONDITIONS

## FINAL REPORT

**TYPE OF PRODUCT:** Face Creams

**PRODUCTS NAMES:** Study Cream Batch A / Study Cream Batch B

**PRODUCTS CODE:** 074785-01 / 074785-02

**STUDY CODE:** All-E-ES-074785-01/02-04-19

**REPORT CODE:** All-E-ES-074785-01/02-04-19-RFV01-Rev01

**REPORT DATE:** 02/11/2020

**SPONSOR:** YUN NV

Enterprise number: 0838.163.142

Berkenlaan 4

2630 – Aartselaar – Belgium

Telephone: +32 473 81 31 26

**Study Responsibility:** Ingmar Claes - Chief Scientific Officer

**STUDY SITE:** ALLERGISA PESQUISA DERMATO-COSMÉTICA LTDA

452, Dr. Romeu Tórtima Avenue – Barão Geraldo

Postal code: 13084-791 – Campinas – SP – Brazil

Telephone: +55 (19) 3789 -8600

**Investigator in charge:** Mariane Martins Mosca

ALLERGISA

# SKIN ACCEPTANCE AND EFFICACY ASSESSMENT OF A TOPICAL PRODUCT IN ACNE TREATMENT WHEN COMPARED TO A PLACEBO, UNDER NORMAL USE CONDITIONS (074785-01/02)

## SUMMARY


**STUDY OBJECTIVES**

The objective of this study was to verify the skin acceptance and efficacy of the product on the acne treatment in comparison with a placebo under normal use conditions. More specifically to evaluate the effect of the 'live' *Lactobacillus* species as 'active ingredient' in relation to acne symptoms."

- *Primary outcomes of the clinical trial:*
  1. reduction of inflammatory lesions is significantly different between study cream A and study cream B
  2. reduction of inflammatory lesions is significantly different between study cream A and study cream B
  3. overall tolerance of the treatment

- *Other parameters evaluated*
  - Clinical Efficacy Assessments and Investigator Global Assessment (IGA) of acne severity, conducted by a dermatologist;
  - Self-Assessment Questionnaires (SAS) and Skin Self-Grading (SFG) by the study subjects; • Assessment of the sebum regulatory action of a product through instrumental analysis of sebumetry;
  - Analysis of images captured by the device Visia CR® (Canfield Scientific, Inc.) – Pores, Porphyrins, Redness of the inflammatory lesions (RBX), Recovery of the Skin barrier (RBX);
  - Microbiological collection for metagenomic analysis.

**METHODOLOGY**

Study subjects remained in a room with controlled temperature and air relative humidity for at least 30 minutes before the initial measurements and in the interval between them. The initial clinical assessment was performed by a dermatologist to confirm the inclusion and non-inclusion criteria and to assess the initial state of the skin (Dermatological Clinical Assessment – IGA and DAT–- T0). Then, a skin sebumetry assessment was performed to confirm the eligibility of the subjects. The subjects approved underwent to an acne lesions counting by a trained technician and facial images of the subjects were obtained to assess the pores, porphyrins, redness of the inflammatory lesions and recover of the skin barrier through the device Visia CR® (Canfield Scientific, Inc.). The collection of the facial material of the subject was performed to extract the microbioma of the DNA present in human skin with the purpose of studying the microbiota metagenomics. The self-assessment (SAS) and skin self-grading (SFG) were performed by the study subjects through questionnaires. The subjects used the product for 08 weeks ± 2 days and then they suspended its use for 04 weeks ± 2 days. They were assessed before product use (T0), after 2, 4 and 8 weeks ± 2 days of product use (T2w, T4w and T8w, respectively) and after 04 weeks ± 2 days without product use (T12w). On all visits, the same assessments performed on the initial visit (T0) were repeated: dermatological assessment, instrumental measurements, images capture, questionnaires and skin material collection for metagenomics. The study subjects should record in their daily-log all the applications performed and add possible comments about the product. They were also be instructed to perform the last product application on the previous day of the assessments at the Institute.

**INVESTIGATOR IN CHARGE** Mariane Martins Mosca

**STUDY LENGTH** 12 weeks.

ALLERGISA

| | |
|---|---|
| **FREQUENCY OF APPLICATION** | Twice daily. |
| **APPLICATION SITE** | Face |
| **POPULATION DESCRIPTION** | Female and Male subjects, aged from 12 to 33 years old (mean age: 18 years old, presenting at least 10 inflammatory lesions and oily skin on the face (minimum sebumetry value 100µg/cm² on frontal area). |
| **NUMBER OF SUBJECTS** | 67 subjects completed the study (36 for Study Cream Batch A and 31 for Study Cream Batch B) |
| **ETHICS** | This study was conducted in conformance with the Declaration of Helsinki principles, the applicable regulatory requirements, including Resolution CNS nº. 466/12, and in spirit of the Good Clinical Practices (Documento de las Américas and ICH E6: Good Clinical Practice). This study was approved by the Local Ethics Committee (LEC) of Investiga - Instituto de Pesquisas, registered by the National Research Ethics Commission (CONEP). |

**RESULTS / CONCLUSION**

***Primary outcomes of the clinical trial:***

**Acne**

Comparing both products, the product Study Cream Batch A presented higher reduction of inflammatory lesions in comparison with the Study Cream Batch B. The same result was observed when the skin redness was evaluated by image analysis, relating the redness to the presence of the inflammatory lesions, in which the skin redness was lower in users of the product Study Cream Batch A than in user of the Study Cream Batch B.

Usually, higher reduction is observed in more intensive treatments, such as those generate by peelings or ingestible medication. The observed reduction is consistent with a cosmetic acne treatment of good quality presenting an average response.

**Dermatological Assessment of Tolerance (DAT)**

Both products can be considered safe. However, the complaints of increase of acne and oiliness from the panel of Study Cream Batch B must be evaluated with caution since the aim of the product is reduction of acne.

**Study Cream Batch A**

During the study 2 cases of adverse events were registered with likely nexus, totalizing 5% of the evaluated population. One subject presented subjective signals after the product use, it was itching. One subject presented objective signal after the product use, it was redness skin.

**Study Cream Batch B**

During the study were registered 5 cases of adverse events with likely nexus. Two subjects presented objective signs after the product use. They presented an increase of acne.Three subjects presented subjective signals after the product use, being ichting and burning and one complaint of increase of skin oiliness. One subject had sensitive skin, however since the study was not focused on population with sensitive skin, this case did not affect the product safety assessment.

Thus, only 4 adverse events were related to the product use, totalizing 10% of the evaluated population.

**Skin Redness – Inflammatory Lesions – Image analysis**

The product Study Cream Batch A promoted a reduction of inflammatory lesions skin redness after 4 weeks of use in relation the product Study Cream Batch B.

**Recovery of the Skin Barrier - Image analysis**

The product Study Cream Batch A promoted a recovery of the skin barrier after 2 and 4 weeks of use in relation the product Study Cream Batch B.

ALLERGISA

**QUALITY ASSURANCE**

The study was conducted according to the Resolution CNS nº. 466/2012, and in the spirit of Good Clinical Practices and in conformity with the Standard Operating Procedures of Allergisa.

Data quality is assured, considering that our personnel is trained according to the study to be carried out, our equipment is always duly calibrated, and the methods used are recognized and/or validated.

The Quality Assurance Department is in charge of auditing the Management System; and is fully available for any specific study monitoring carried out by the sponsor.

The signature below means that the study was conducted as described above.

Heliara Lopes do Nascimento
Quality Assurance Manager
02/11/2020

CONTENTS

1 ABBREVIATION LIST ........ 8
2 INTRODUCTION ........ 10
2.1. Acne ........ 11
2.2. Porphyrins ........ 11
3 OBJECTIVE ........ 12
4 INVESTIGATIONAL PRODUCTS ........ 12
4.1. Identification ........ 13
4.2. Use Directions ........ 13
4.3. Product Use Compliance Check ........ 13
4.4. Storage ........ 13
5 APPLICABLE ETHICAL REMARKS ........ 13
5.1. Confidentiality of obtained data ........ 14
5.2. Study Subjects ........ 14
5.3. Informed Consent Form (ICF), Acceptance Form (AF) and Informed Consent Image Release (ICIR) ........ 14
5.4. Independent Ethics Committee ........ 14
6 STUDY PERIOD ........ 14
7 STUDY SUBJECTS ........ 15
7.1. Study subjects Recruitment ........ 15
7.2. Selection and Admission of Study Subjects ........ 15
7.3. Description of included population ........ 15
7.4. Inclusion Criteria ........ 16
7.5. Non-Inclusion Criteria ........ 16
7.6. Injunction and Constraint ........ 16
8 METHODOLOGY ........ 17
8.1. Study Design ........ 17
8.2. Materials and Equipment ........ 17
8.3. Test Site ........ 17

8.4. General Procedures ........ 17

1.1. Procedure Schedule ........ 26

8.5. Criteria and Procedures for Study Subjects Withdrawal ........ 26

9 ADVERSE EVENTS ........ 27

10 STATISTICAL ANALYSIS ........ 28

10.1. Objective parameter (Instrumental analysis – Sebumeter and Visia CR® and Counting of Acne Lesions) ........ 29

10.2. Ordinal and interval scaled data (Dermatological Clinical Assessment, Investigator Global Assessment (IGA) of acne severity and Self-Grading (SFG)) ........ 29

10.3. Nominal data (Self-Assessment (SAS)) ........ 30

11 RESULTS ........ 31

11.1. Protocol Deviation ........ 31

11.2. Adherence to the Study ........ 31

11.3. Occurrences during the Study ........ 32

11.4. *Primary outcomes of the clinical trial:* ........ 35

11.5. Acne ........ 35

11.6. Dermatological Assessment of Tolerance (DAT) ........ 48

11.7. Assessments of Acne Severity (IGA) ........ 52

11.8. Self-Assessment Questionnaire (SAS) ........ 56

11.9. Self-Grading Assessments (SFG) ........ 68

11.10. Sebumeter (Skin Oiliness) ........ 119

11.11. Pores ........ 123

11.12. Porphyrins Percentage ........ 130

11.13. Total Surface of Redness – Inflammatory Lesions ........ 133

11.14. Total Surface of Redness – Recovery of the Skin Barrier ........ 136

11.15. Best Cases ........ 141

12 CONCLUSION ........ 145

12.1. Acne ........ 145

12.2. Dermatological Assessment of Tolerance (DAT) ........ 146

12.3. Skin Redness – Inflammatory Lesions ........ 146

12.4. Recovery of the Skin Barrier ........ 147
12.5. Assessments of Acne Severity (IGA) ........ 147
12.6. Self-Assessment Questionnaire (SAS) ........ 148
12.7. Self-Grading Assessments (SFG) ........ 151
12.8. Skin Oiliness ........ 152
12.9. Pores ........ 152
12.10. Porphyrins ........ 153
13 REFERENCES ........ 154
APPENDIX 1. INFORMED CONSENT FORM ........ 156
APPENDIX 2. ACCEPTANCE FORM - TEENAGER ........ 164
APPENDIX 3. INFORMED CONSENT FORM AUTHORIZATION LEGAL GUARDIAN(S) ........ 170
APPENDIX 4. INFORMED CONSENT FOR IMAGE RELEASE (ICIR) ........ 179
APPENDIX 5. STUDY GROUP ........ 180
APPENDIX 6. RANDOMIZATION ........ 183
APPENDIX 7. QUESTIONNAIRES ........ 185
APPENDIX 8. RAW DATA AND STATISTICAL ANALYSIS ........ 201
APPENDIX 9. DNA EXTRACTION AND QUANTIFICATION ........ 303
APPENDIX 10. PRODUCT INFORMATION ........ 305

# 1 ABBREVIATION LIST

| | |
|---|---|
| °C | Celsius Degree |
| µg/cm² | Microgram per centimeter square |
| AF | Acceptance Form |
| ANOVA | Analysis of variance |
| ANVISA | Agência Nacional de Vigilância Sanitária (National Health Surveillance Agency). |
| cm | Centimeter |
| cm² | Centimeter square |
| CNS | Conselho Nacional de Saúde (National Health Council) |
| CONEP | National Research Ethics Commission |
| CRM | Regional Council of Medicine |
| DAT | Dermatological Assessment of Tolerance |
| DNA | Deoxyribonucleic Acid |
| Dr. | Doctor |
| e.g. | For example |
| EDTA | Ethylenediamine Tetraacetic Acid |
| etc | *Et cetera* |
| FDA | Food and Drug Administration |
| GCP | Good Clinical Practices |
| HIV | Human Immunodeficiency Virus |
| ICF | Informed Consent Form |
| ICH | Good Clinical Practice |
| ICIR | Informed Consent Image Release |
| IEC | Independent Ethics Committee |
| IGA | Investigator Global Assessment |
| LEC | Local Ethics Committee |
| LSD | Least significant difference |
| Ltda | Limited |
| mL | Mililiter |
| mM | Milimolar |
| nº | Number |
| pH | Hydrogen Potential |
| RBX | Red Brown X color space |
| RGB | Red Green Blue color space |
| RH | Relative Humidity |
| ROI | Region of Interest |
| SAS | Self-Assessment of Product Performance questionnaire |
| SFG | Self-Grading Assessments |

| | |
|---|---|
| SP | São Paulo |
| Txx | Time-points os the study |
| UV | Ultraviolet |
| UV | Ultra-violet |
| UVA | Ultraviolet A |

## 2 INTRODUCTION

Industry awareness and consumer's and regulatory agencies requirements caused cosmetic manufacturers to adopt procedures that lead them to know better their products: to conduct clinical tests on safety and efficacy, which are coordinated by expert physicians, before marketing a product. These procedures provide cosmetic companies with greater safety, credibility and reliability among their consumers.

Once the cosmetic product becomes freely available for the consumer, it must be safe when applied under normal or reasonably foreseeable conditions of use - ANVISA Guide for the Safety Evaluation of Cosmetic Products (Guia para Avaliação de Segurança de Produtos Cosméticos da ANVISA). For this, the raw materials used in the product formulation should be raw materials with proved safety and with established use in the cosmetic industry. In addition, the safety of the final formula must be tested before it is marketed.

The acceptance studies assess the safety of the products under real-use conditions, which allows knowing the product under the same marketed conditions. Therefore, in-use studies are performed with the finished product, before it is introduced into the market. (BARAN & MAIBACH, 1994). The objective of cosmetics safety assessment studies is to confirm the absence of risks associated with using the cosmetic product. In order to evaluate the irritation and sensitization potential of a product, a series of variables should be taken into account: components used in the formulation, ingredient concentration, absorption, amount applied, skin condition, application directions and frequency, as well as the cumulative effect (DOOMS-GOOSSENS, 1993).

According to the Good Clinical Practices, an adverse event is any untoward medical occurrence in a study subject or clinical investigation subject using a product, which does not necessarily have a causal relationship with the treatment (International Conference on Harmonisation of Technical Requirements for Registration of Pharmaceuticals for Human Use – ICH).

The contact of the skin with topical products, such as cosmetic products, may trigger different types of reactions. Among these adverse reactions, we can point out eczematous contact dermatitis, urticaria, acne and spots (SAMPAIO & RIVITTI, 2000). In general, the contact dermatitis results from two mechanisms: the primary irritation, through the action of irritant substances; or the sensitization, in the presence of an allergenic ingredient.

Tests conducted with humans are governed by very strict laws in order to protect and safeguard people. These laws vary from country to country. In Brazil, these studies are allowed, provided that they comply with the precepts of the Declaration of Helsinki and the CNS 466/12 Resolution (NATIONAL HEALTH COUNCIL, 2013).

In addition to safety, this study can also assess sensory characteristics of the product, and detect additional complaints and comments as to its "performance". Concerning the support of claims for cosmetic products, the following directives were published by COLIPA (2001):

The benefits provided by a cosmetic product must be consistent with the consumers' expectations generated by the claim; In order to evaluate if a claim is appropriate, it is necessary to take into account

the general consumers' impression concerning the presentation or the product advertisement. The *claims* must be supported by solid, clear and relevant evidences. Such evidences may result from experimental studies (biochemical/instrumental methods, sensory evaluations, technical evaluations and evaluations without the participation of study subjects: in vitro testing in cell cultures, use of hair locks, etc), and consumers' evaluations. (ASTM E 1958-06).

By performing clinical studies, the company has the opportunity to know in advance the possible considerations and complaints that may arise when the product is marketed, being able to develop strategies, such as specific training for its Consumer Service Staff before launching the product (BARAN & MAIBACH, 1994).

## 2.1. Acne

Acne is a very common dermatological problem, which affects a significant portion of the world population, and mainly individuals in the 11-25 age range. It is characterized by comedones, papules, pustules, cysts and/or scars, located mostly on the “seborrheic areas”, which are body regions with a higher amount of sebaceous glands – center-facial area, dorsum and thorax (VIGLIOGLIA & RUBIN, 1991).

In physiopathology, one can observe as important elements on this subject: the increase of sebaceous secretion by the gland; follicular hyperkeratosis with subsequent obstruction, causing keratin and sebum accumulation in the follicle; and bacteria colonization and inflammation (SAMPAIO & RIVITTI, 2000).

Its causes are not fully known; however, it is known that genetic aspects and emotional / immunological / hormonal / environmental factors are involved in the genesis or aggravation of the picture, as well as the alteration of the local flora, with an expansion of the bacterial population, especially the *Propioniumbacterium acnes*.

The efficacy evaluation of a product with antiacne properties may be performed through in vitro and experimental studies in laboratory animals or in human beings. Studies in animals are meant to toxicity assessment, when there are unknown drugs in the product. The in vitro studies determine the antimicrobial capacity of the product over the microorganism which are usually present in the process (*P. acne*) (VIGLIOGLIA & RUBIN, 1991). In humans, it is possible to assess the action of the products under real use conditions, considering the different types of skin.

## 2.2. Porphyrins

In the pilosebaceous follicles, the porphyrins are produced by *Propionibacterium acnes* (*P. acnes*), which are normal inhabitants of the human skin and the pilosebaceous ducts. Porphyrins are high fluorescent pores containing P. acnes and the porphyrins associated appear red-orange when the face skin is illuminated with UVA radiation of long wavelength. It is known that the intensity of the follicular fluorescence and its extension of the facial involvement are proportional to the density of P. acnes. Porphyrin fluorescence appears first on the nose and on the chin, and later increases the incidence on the adult age and decreases after 50 years old, possibly reflecting the sebum secretion rate.

The porphyrins may also have cytotoxic and comedogenic potential. With sun exposure, the porphyrins may liberate singlet oxygen that, in its turn, may oxidate lipids (lipidic peroxidation) on the skin,

producing irritating lipidic peroxides and cytotoxics that may cause skin damage. Thus, the presence of porphyrins on the follicular pores may accentuate or accelerate the hyperpigmentation, wrinkles and other signs of photoaging by sun exposure.

## 3 OBJECTIVE

The objective of this study was to verify the skin acceptance and efficacy of the product on the acne treatment in comparison with a placebo under normal use conditions. More specifically to evaluate the effect of the 'live' *Lactobacillus* species as 'active ingredient' in relation to acne symptoms."

- *Primary outcomes of the clinical trial:*
  - 1. reduction of inflammatory lesions is significantly different between study cream A and study cream B;
  - 2. reduction of inflammatory lesions is significantly different between study cream A and study cream B;
  - 3. overall tolerance of the treatment - Dermatological Assessment of Tolerance (DAT)

- *Other parameters evaluated*
  - Clinical Efficacy Assessments and Investigator Global Assessment (IGA) of acne severity, conducted by a dermatologist;
  - Self-Assessment Questionnaires (SAS) and Skin Self-Grading (SFG) by the study subjects; • Assessment of the sebum regulatory action of a product through instrumental analysis of sebumetry;
  - Analysis of images captured by the device Visia CR® (Canfield Scientific, Inc.) – Pores, Porphyrins, Redness of the inflammatory lesions (RBX), Recovery of the Skin barrier (RBX);
  - Microbiological collection for metagenomic analysis.

## 4 INVESTIGATIONAL PRODUCTS

Products information, as declared by the Sponsor, are described in Appendix 10. A sample of each product was cataloged and it can be found in Allergisa's files until the study final report is confirmed.

## 4.1. Identification

Table 1. Investigational products identification

| Product Name | Type of Product | Product Code |
|---|---|---|
| Study Cream Batch A | Face Cream | 074785-01 |
| Study Cream Batch B | Face Cream | 074785-02 |
| YUN's SKN Wash | Shower Gel | Auxiliary Product |

## 4.2. Use Directions

**FACIAL CREAM (Study Cream Batch A / Study Cream Batch B):** Apply the product twice a day, once in the morning and once at night (for example, before going to bed).
**AUXILIARY PRODUCT:** May be used daily for washing, in replacement of the usual soap.

## 4.3. Product Use Compliance Check

The compliance of product use by the subjects was checked through the daily log completed by the subjects and through the weighing of the product before and after 8 weeks of use.

## 4.4. Storage

All products sent by the sponsor were initially stored in the samples room at the study center, with controlled temperature and restricted access. Products release was controlled by the principal investigator or by a previously designated technical staff.

At the moment of being given the product, the subjects will be instructed on how to correctly store it and to keep it out of reach of children and/or animals.

# 5 APPLICABLE ETHICAL REMARKS

This study was conducted in conformance with the Declaration of Helsinki principles, the applicable regulatory requirements, including Resolution CNS nº. 466/12, and in the spirit of the Good Clinical Practice principles (Documento de las Américas and ICH E6: Good Clinical Practice).

Before the study starts, the subjects were informed about its objective, methodology and duration, and about the possibly expected benefits and the constraints related to the study. An Informed Consent Form (Appendix 1) and an Informed Consent Image Release Form (Appendix 4), written in conformity with the Declaration of Helsinki and Resolution CNS No 466/2012 and approved by the Independent Ethics Committee (IEC) of Investiga - Instituto de Pesquisas, registered by the National Ethics Commission [Comissão de Ética em Pesquisa (CONEP)] were signed by the study subjects.

In the case of subjects under 18 years old, an Acceptance Form should have been signed by the minor for their participation (Appendix 2), and the legal guardian must have been also signed a form authorizing the minor's participation (Appendix 3).

## 5.1. Confidentiality of obtained data

All data to be found or proved by the study results were considered as being confidential information and sponsor's property. No information - as well as all documents generated during the study - should be copied or disclosed without a previous written consent of the sponsor. All information are kept confidential until the results are published.

## 5.2. Study Subjects

In order to maintain confidentiality of subjects' data, all data collected were identified by a number they was given at the beginning of the study. No personal information was be disclosed together with study data. If required, the Investigator in charge could allow the study monitor to access all study-related subjects' data. This must include all documents containing the subject's clinical history for checking suitability for the study, diagnoses and any other document concerning the subject in the study.

## 5.3. Informed Consent Form (ICF), Acceptance Form (AF) and Informed Consent Image Release (ICIR)

The process of obtaining the ICF must confirm the voluntary nature of subjects participation in the study. All study-related aspects were explained to the subject, before they sign the ICF, AF and the ICIR. The investigator in charge is completely responsible for obtaining the ICF and AF in compliance with the specification of the GCP, Resolution CNS no. 466/2012 and the international regulatory requirements (ICH).

## 5.4. Independent Ethics Committee

The study will be conducted in compliance with the ICH directives for Good Clinical Practices and carried out based on the ethical principles established in the CNS Resolution no. 466/2012.

Before the beginning of the study, the protocol, the Informed Consent form (ICF), the Acceptance Form (AF) and the Informed Consent For Image Release (ICIR) will be sent for review by the Investigator Instituto de Pesquisas Independent Ethics Committee (IEC) for written approval. Any written information given to the subject and all notifications and amendments to the study will also be sent to this IEC. The unified statement issued by the IEC will be archived with the study documents kept by the study site.

The study technical documentation is in Allergisa files, where it will be archived for a 15-year period.

# 6 STUDY PERIOD

The total duration of the study was 17 weeks. The total duration of the study for each subject was 12 weeks ± 02 days.

- **Start Date of the first group:** 05/14/2019;
- **End Date of the last group:** 09/09/2019.

# 7 STUDY SUBJECTS

## 7.1. Study subjects Recruitment

The study subjects were recruited by the recruitment department of the Study Center that has a computerized and updated register system. The subjects registered into this system are interested in participating in clinical trials. They were contacted and asked to take part in the selection process and if they met all required criteria, they would be included in the study.

## 7.2. Selection and Admission of Study Subjects

During the subjects' selection for the study, the physician in charge certified that the subjects had no pathologies that could interfere with the study results. The physician is also responsible for all information contained in the subject's assessment form, by checking all inclusion and non-inclusion criteria for admission of the subject in the study.

## 7.3. Description of included population

The description of the population included in the study is available on the following table. The detailed description of the population per subject is available in the Appendix 5.

The list of subjects randomized by investigational product is available at Appendix 6.

Table 2. Study Population Description

| Total of subjects | | | | | | Age (years old) | | |
|---|---|---|---|---|---|---|---|---|
| Recruited[1] | Included[2] | Excluded[3] | Withdrawal[4] | Gender F | Gender M | Mínimum | Maximum | Mean |
| 100 | 79 | 21 | 00 | 49 | 30 | 12 | 33 | 18 |

*[1]subjects who attended the Institute and signed the ICF.*

*[2]subjects that were approved in the study.*

*[3]Subjects that did not meet the inclusion criteria or presented any of the non-inclusion criteria.*

*[4]subjects that withdrew from the study after the study consent for personal reasons and were not included.*

*Caption: F=Female; M=Male*

The description of the population randomization included in the study is available on the following table. The list of subjects randomized by investigational product is available at Appendix 6.

Table 3. Study Population Randomization

| Treatment | Total of subjects | | | Age (years old) | | |
|---|---|---|---|---|---|---|
| | Included | Gender F | Gender M | Mínimum | Maximum | Mean |
| Study Cream A | 40 | 24 | 16 | 12 | 29 | 17 |
| Study Cream B | 39 | 25 | 14 | 13 | 33 | 18 |

### 7.4. Inclusion Criteria

- Healthy skin in the test areas;
- Subjects willing and capable to follow the study rules and a fixed schedule;
- Ability of giving consent for participation in the study;
- Subjects with good health in general and good mental condition;
- Any gender;
- 12 to 35 years old;
- Subjects who present at least 10 inflammatory lesions;
- Oily skin on the face (minimum sebumetry value 100μg/cm² on frontal area (mean: 3 measurements)).

### 7.5. Non-Inclusion Criteria

- Pregnancy or breastfeeding;
- Subjects who present severe acne;
- Subjects who present more than two nodular lesions;
- Subjects who changed their oral contraception method up to three months before the study beginning;
- Subjects who did acne hormonal treatment less than 6 months before the study;
- Subjects who did oral isotretinoïne treatment less than 1 month before the study;
- Subjects who did topical acne treatment less than 90 months before the study;
- Subjects who did aesthetic treatment less than 6 months before the study (like laser and peeling);
- Subjects who did treatment with antibiotics within the last 4 months;
- Simultaneous participation in different studies from external research institutes on the same test sites;
- Inadequate language proficiency (spoken and written);
- Participate in the study under the influence of alcohol and/or drugs as well as addiction;
- Severe psychological disease or intellectual disability of understanding the study;
- Severe disease (heart/circulatory/liver, kidney and lungs disease, severe diabetes mellitus) or chronic infections (hepatitis, HIV);
- Immune insufficiency;
- Current use of the following topical or systemic medications: corticosteroids, immunosuppressive and anti-histaminic drugs;
- Skin diseases: vitiligo, psoriasis, atopic dermatitis;
- Confirmed allergies to cosmetic components or previous responses of intolerance after the application of cosmetic products of the same category of the investigational products;
- Other diseases or medications that might directly interfere in the study or put the subject's health under risk.

### 7.6. Injunction and Constraint

- Do not apply any other product to the test site;
- Do not change any cosmetic habits, including personal hygiene;

- Do not perform and aesthetic or dermatological treatments on the test region during the study period;
- Do not expose to sunlight excessively.

# 8 METHODOLOGY

## 8.1. Study Design

Comparative double-blind placebo-controlled randomized clinical study. The blinding comprised: studied subjects, dermatologists, trained techinicians, investigators, statisticians and the writer of the report.

## 8.2. Materials and Equipment

- Visia CR® (Canfield Scientific, Inc.);
- Sebumeter SM 815 (Courage & Khazaka electronic GmbH);
- Template of 6.0cm x 5.0cm;
- Template of 4.0cm x 4.0cm;
- Template of 2.5cm x 2.5cm;
- Black cover;
- Black hair band;
- Thermohygrometer;
- Acclimated Room;
- Magnifying glass with light;
- Gloves, masks and caps;
- kit QIAamp PowerFecal DNA kit (Qiagen);
- Equipment Qubit 4 Fluorometer – ThermoFischer Scientific.
- 1 plastic bag containing:
  - 1 sterile cotton swab (Bac-Swab brand DME sterile),
  - 1 tube of 15 mL with collection saline solution (Tris, 50mM pH 7.6; EDTA 1mM pH 8.0, Twen 20 0.5%)
  - 1 tube of 2mL with DNA conserving liquid;
  - A sterile paper mask, disposable with an area of 12.5 cm² for collection;

Disposable gloves, a pair of scissors and a timer to mark the non-provided collection time.

## 8.3. Test Site

The products were applied to the study subjects' face.

## 8.4. General Procedures

The subjects were informed about the study objective, methodology and duration, and about the possible expected benefits and the constraints related to the study. Those who agreed in participated in the study signed an Informed Consent Form (Appendix 1) and the Informed Consent for Image Release (Appendix 2).

ALLERGISA

Study subjects remained in a room with controlled temperature and air relative humidity (20°C ± 2°C and 50% ± 5 RH) for at least 30 minutes before the initial measurements and in the interval between them. The initial clinical assessment was performed by a dermatologist to confirm the inclusion and non-inclusion criteria and to assess the initial state of the skin (Dermatological Clinical Assessment – IGA and DAT - T0). Then, a skin sebumetry assessment was performed to confirm the eligibility of the subjects. The subjects approved underwent to an acne lesions counting by a trained technician and facial images of the subjects were obtained to assess the pores, porphyrins, redness of the inflammatory lesions and recover of the skin barrier through the device Visia CR® (Canfield Scientific, Inc.). The collection of the facial material of the subject was performed to extract the microbioma of the DNA present in human skin with the purpose of studying the microbiota metagenomics. The self-assessment (SAS) and skin self-grading (SFG) were performed by the study subjects through questionnaires.

The subjects used the product for 08 weeks ± 2 days and then they suspended its use for 04 weeks ± 2 days. They were assessed before product use (T0), after 2, 4 and 8 weeks ± 2 days of product use (T2w, T4w and T8w, respectively) and after 04 weeks ± 2 days without product use (T12w). On all visits, the same assessments performed on the initial visit (T0) were repeated: dermatological assessment, instrumental measurements, images capture, questionnaires and skin material collection for metagenomics.

The study subjects should have recorded in the daily-log of the product use all the applications performed and possible comments about the product. They were also be instructed to perform the last product application on the previous day of the assessments at the Institute.

### 8.4.1. Dermatological Clinical Assessment (DAT and IGA)

The subjects were assessed by a dermatologist on all the visits. On the initial visit (T0) to verify the inclusion and non-inclusion criteria of the study and assessment of the clinical efficacy parameters and on the other visits (T2w / T4w / T8w / T12w), to assess the safety (DAT) and clinical efficacy (IGA) (Appendix 7).

#### 8.4.1.1. Dermatological Assessment of Tolerance (DAT)

On all visits, the dermatologist performed an assessment of the study subjects' faces according to a 5-point scale (Appendix 7). The physician recorded in the subject's case report form possible discomforts sensation informed.

Subjects were also supervised by the physician throughout the study and were also assessed in case there was any symptom or sign, in order to confirm the correct and accurate use of the product and to detect possible adverse events.

Subjects were instructed to contact the study coordinator at any time, in case they presented any complaints. In these cases, they would be sent for assessment and guidance by the dermatologist in charge, who would conduct a dermatological examination, then, they would rate the reaction and follow the appropriate procedure (guidance and/ or medication and photographic documentation, when necessary).

#### 8.4.1.2. Assessment of the Acne Severity (IGA)

This methodology of global assessment consists in the use of a scale pre-defined by the FDA (Food and Drug Administration) in 2005, for the scoring of acne severity grade (Appendix 7).

This scale has five degrees of severity (0-4), in which each class is defined by a clinically relevant and morphologically different description, with the objective of minimizing the variability among those who evaluate or diagnose (COSTA, 2005). The five characteristics are described below:

- Grade 0: Clean - no non-inflammatory and inflammatory lesions
- Grade 1: Almost clean - Almost clean; rare non-inflammatory lesions, with more than one small inflammatory lesion.
- Grade 2: Mild – Mild severity; superior to Grade 1; a few non-inflammatory lesions with no more than a few inflammatory lesions (papules / pustules only, no nodular lesions).
- Grade 3: Moderate - Moderate severity: higher than Grade 2: up to a lot of non-inflammatory lesions and there can be a few inflammatory lesions, no more than one small nodular lesion
- Grade 4: Severe - Severe; higher than Grade 3: up to a lot of inflammatory and non-inflammatory lesions, no more than a few nodular lesions.

The global assessment of the degree of acne severity was performed by a dermatologist on all the study time-points.

### 8.4.2. Counting of Acne Lesions

The subjects were assessed by a trained technician in order to perform the acne lesions counting and scoring on 5 face areas. The counting of the acneic lesions was performed according to the description on Table 4.

Table 4. Types of lesions assessed

| | |
|---|---|
| Open comedones | Corneo-sebaceous masses with a visible surface (dark spots) without signs of inflammation. |
| Closed comedones | Slightly papulous corneo-sebaceous masses, with whitish or normal surface coloring. |
| Papules | Skin bumps with pinkish or reddened coloration, which might be painful. |
| Pustules | Skin bumps with visible pus secretion in the center. |

The counting was performed with the aid of a surface magnifying glass and templates with fixed area.

The areas assessed were the malar, the mentum and the frontal area. The inflammatory lesions were counted on the whole face, while the non-inflammatory lesions were counted in specific templates, according to what is described below:

- Right and left malar (a 6.0cm x 5.0cm template placed according to the nasal wing fold in each half-face);
- Mentum (a 2.5cm x 2.5cm template placed on the mentum central portion);
- Right and left frontal area (a 4.0 x 4.0cm mold was laterally placed to the glabella).

The assessments were performed on all the study time-points.

#### 8.4.3. Self-Assessment (SAS) and Self-Grading of the Skin (SFG) by the Study Subjects

The study subjects were instructed to assess the skin of their face through self-assessment questionnaires (SAS) and skin self-grading (SFG).

So subjects knew the meaning of each tested attribute, they were instructed by a trained technician.

While the SAS questionnaires were completed only on T2w, T4w and T8w, the SFG questionnaires were completed on all the study time-points.

#### 8.4.4. Skin Oiliness Instrumental Evaluation with the equipment Sebumeter SM 815 (Courage & Khazaka electronic GmbH)

The skin oiliness measurements were performed by duly trained technicians, by using the equipment Sebumeter SM 815, Courage & Khazaka electronic GmbH. Measurements are based on photometry of a special translucent plastic tape, which becomes transparent in presence of lipids. This tape is applied to the skin for thirty seconds and the transparency of the tape is then measured by the device. A 1cm² measurement area is used. The results provided by the device are expressed in µg/cm².

To confirm the inclusion criteria of oily skin, three measurements of the frontal area were performed on the subjects. The subjects who presented at least 100 µg/cm² of sebumetry, as mean of the three measurements, were considered included in the study.

The measurements were performed on all the study time-points.

#### 8.4.5. Assessment with the Equipment Visia CR® (Canfield Scientific, Inc.);

Three images were obtained of the subjects' face, being 1 frontal and 2 lateral through the device Visia CR® (Canfield Scientific, Inc.). In this image, the subject's identity was preserved, and, by signing the

informed consent for image release, the subject gave his or her written consented for obtaining and releasing the image.

This device captures face digital photographs and it has been especially designed to allow the light emitted to deliver the ideal lighting for achieving good results. The system allows capturing and storing images, using visible light (Standard), polarized light flash and UV rays flash.

Subjects were instructed to keep eyes gently closed while images were being taken.

The images were taken on all study time-points.

#### 8.4.6. Image Analysis through the Software FrameScan®

The images obtained through the equipment Visia CR® (Canfield Scientific, Inc.) were analyzed through the software FrameScan®, which is used for analysis of colorimetric images and/or quantitative morphological of digital photographic images. The software allows the extraction of images characteristics such as colorimetry measurements, luminosity, vascularization, pigmentation or homogeneity, in addition to morphology analysis of elements such as pigment spots, eyelashes, wrinkles, among others. Due to those characteristics, this software is commonly used to measure the effect of several products targeted to skincare.

In this study, parameters were used to analyze pores, porphyrins, redness of inflammatory lesions and facial skin barrier recovering of the study subjects. These parameters were calculated from a mask designed in an interest area, on the image of each subject, obtained in time-point T0 and replicated in the image obtained in time-points T2w, T4w, T8w and T12w, assuring the same area in all time-points of the study.

##### 8.4.6.1. Pores Assessment

The Standard 2 images obtained through the equipment Visia CR® (Canfield Scientific, Inc.) were analyzed through the software FrameScan®; which is used for quantitative morphological analysis of digital photographic images. This light consists of a photography with diffuse flashes of light, leading to a slight brightness reduction. An assessment will be performed by the Software FrameScan®.

A small ROI is marked on the cheek region, where there is a high concentration of pores and the size and number of pores a recognized by the software in pixels. The image binarization was performed to delimit the affected surface by the enlarged pores.

The parameters used for the pores analysis was *number of pores* and *total surface*. The *number of pores* is the amount of pores recognized by the software in the evaluated area. The recognition occurs through the discrimination of continuous blocks of dark pixels, in which each block is recognized as one pore. The *total surface* is the sum of the pixels present in the pores on the selected area on the face. The numerical reduction of both parameters shows the pores visibility reduction on subjects.

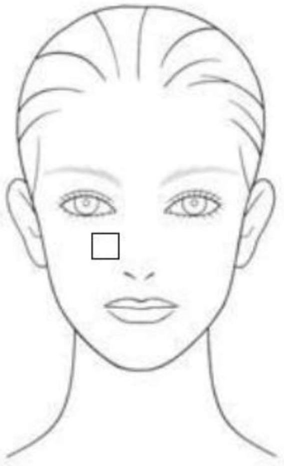

Figure 1. Example of the evaluated area for the pores analysis

#### 8.4.6.2. Porphyrins Assessment

The images obtained through the device Visia CR® (Canfield Scientific, Inc.) were analyzed by the software ImageJ®. For this assessment, right and left lateral images and frontal UV images were analyzed. The images UV and UV2 are ideal for this type of analysis, because porphyrins present an orange-red color in this type of light (Figure 2). The image was thresholded, highlighting the porphyrins.

On areas where the porphyrins are more numerous or intense, the region of interest (ROI) is delimited and all the porphyrins on this region are recognized by this software (Figure 3). This area can be the cheek, nose or forehead, according to the characteristics of each subject, since these regions have a high incidence of porphyrins. Then, the percentage of the area occupied by the porphyrins inside the ROI is calculated (Figure 4). The decrease of the percentage of the area occupied indicated the decrease of the amount of porphyrins on the subjects' skin, which indicates, in turn, the reduction of the population of *P. acnes.*

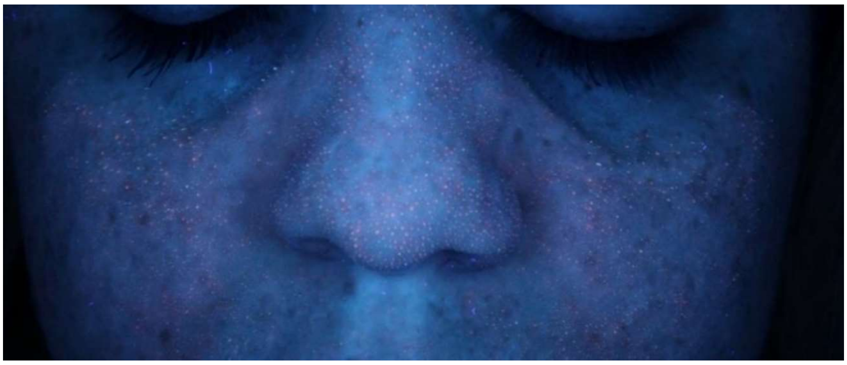

Figure 2. Example of the UV image and view of the porphyrins.

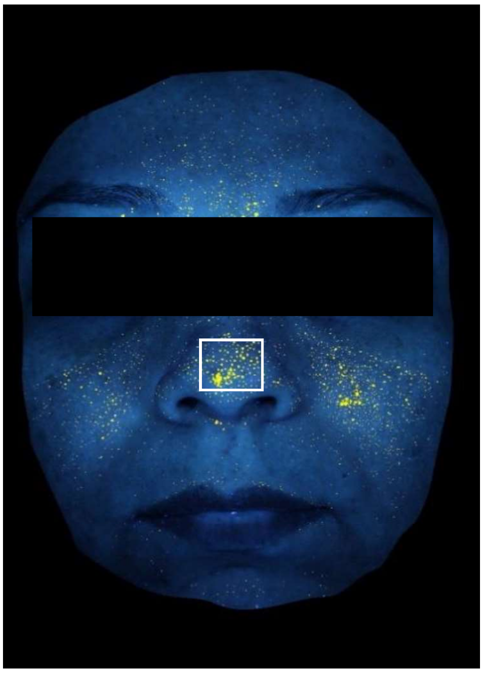

Figure 3. UV image with the mask (yellow points) after tabulation and area selected for analysis

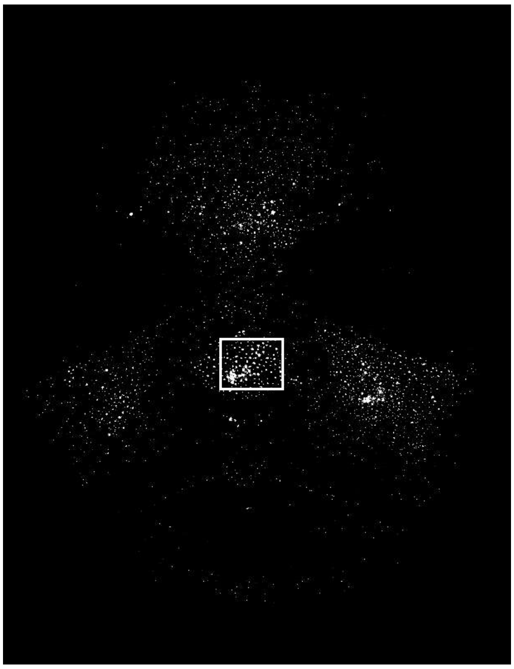

Figure 4. Image of the porphyrins mask after tabulation and area selected for analysis.

#### 8.4.6.3. Assessment of the skin redness and recovery of Skin Barrier

The image analysis for the skin redness assessment was performed through the software FrameScan® in the module analysis Morphology of Spots, using the lateral images (left and right) with red filter RBX facial (it enhances the face vascularization). The image of the skin captured with the digital camera is composed of Red (R), Green (G) and Blue (B) and is presented on the native space RGB of the camera. The RBX transforms this RGB image into the RBX color-space where the Red and Brown channels represent hemoglobin and melanin, respectively. The red filter, used for this analysis, offers the view of the vascular conditions of each subject.

Two analysis were done through the RBX images. In one approach, the skin redness caused by skin acne was assessed, and on another approach, the integrity of the barrier function, both analyses differed from each other, due to the assessment area, where the redness is generated by different skin dysfunctions.

On the analysis of the redness caused by acne, the analyst selected a large area in one of the lateral sides of the face (in the cheek area), taken by acne lesions. The threshold was assigned to highlight acne lesions and thus, the inflammatory lesions present in the area were considered in the analysis, as shown in Figure 5. Thus, this analysis was focused on the reduction of inflammatory lesions redness.

On the analysis of integrity of the barrier function, the area selected for this analysis is positioned near the nose, chosing areas without inflammatory lesions. At the T zone the oiliness is high, which might affect the skin barrier leading to subclinic inflammatory process. Since disruption of the skin barrier present

on the stratum corneum has been correlated with some dermatosis, the reduction of the skin irritation may be related to a higher integrity of the barrier function (Elias *et al* 1999).

For both analysis, the software replicates the ROI automatically on the other images obtained in all the experimental time-points for each subject. The total area of redness was the parameter assessed and it is given in pixels. The binarization of the image (that is, the thresholding between red and white) was done to delimit through the redness (black areas after the binarization) and the portion of the skin to be assessed (Figure 5).

The parameter used for the skin irritation analysis was:

- Total Surface: This parameter is the total of the redness area, given by the sum of the number of pixels recognized as red (microvascularization). With the treatment, it was expected a reduction of this parameter, indicating that the skin irritation was reduced.

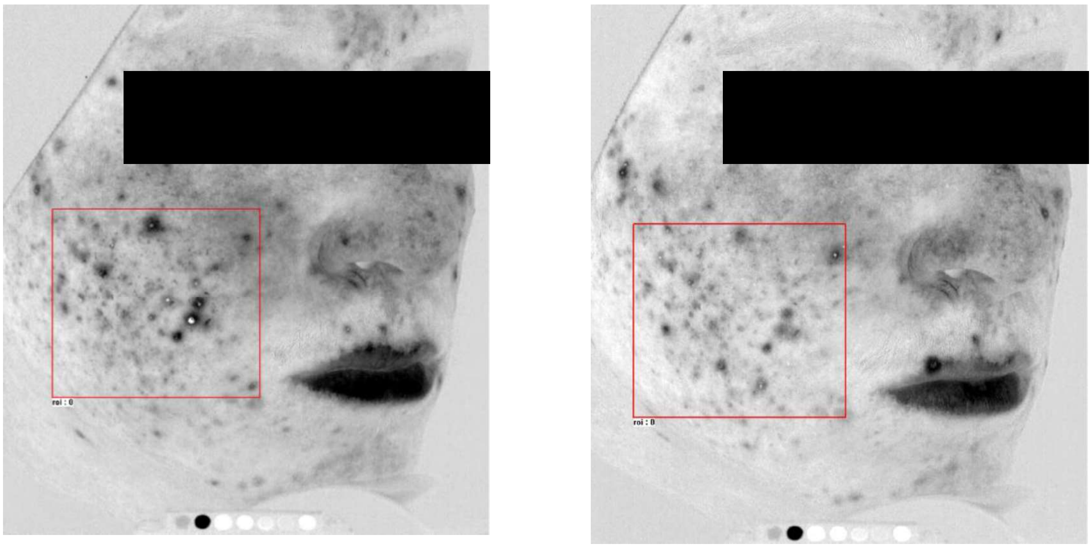

Figure 5. Example of area selected for redness analysis, comprising the inflammatory lesions on the same area, before (left) and after (right) the treatment.

### 8.4.7. Microbiota Collection by non-invasive Method

A human skin microbioma collection was performed as well as an extraction of the DNA of this microbioma with the purpose of studying the metagenomic of the microbiota. The collection was be performed on the subjects' faces.

The microorganisms studied are part of the natural skin microbiota. All the human material, such as the skin surface cells, were ignored and not used for analysis.

The collection of the study subjects microbiota was be done by a technician of Allergisa trained by Gentros. The collection was performed in a non-invasive way, with the help of a sterile swab (Bac-Swab brand DME sterile) soaked in saline solution (Tris, 50mM pH 7.6; EDTA 1mM pH 8.0; Twen 20 0.5%). The area of collection was standardized with the help of a sterile, flexible, plastic and individual mold, which limits the area of the collection to 12.5cm$^2$. The swab was gently rubbed over this restricted area by the template for 15 seconds. Right after the collection of the swab's stem, it was immersed in a tube of 2mL

with conserving liquid to avoid the degradation of the DNA and the stem cutted in a way that only the tip containing the material collect was stored in the tube. Tubes containing the swabs was identified with the subjects number. Once the collection was finished, the tubes were stored at 4°C until the microbioma DNA extraction for a maximum of one week.

The collection was performed on all study time-points.

##### 8.4.7.1.1 Collection procedure

- 1. The date and time of the start of the procedure and the indicate area of collection on the human body scheme were recorded on the subject’s file;
- 2. Surgical gloves were used during the procedure;
- 3. When opening the cotton swab, the stem did not touch where the rubbing was done;
- 4. The paper mask was brought closer to the collection region always on the position A-B in relation to who was collecting;
- 5. The cotton swab was wetted on the saline solution and rubbed during 15 seconds inside the mask-free area;
- 6. The cotton swab was inserted in the 2mL tube with conserving liquid, and cutted, with the help of scissors, the remnant stem; the tube was closed well. Attention was paid to do not let the conserving liquid spill.
- 7. The paper with the area of collection and the 2mL tube with the collection were gave back.

#### 8.4.7.2. Extraction and Quantifying of the extracted DNA

A DNA extraction of microorganisms of the microbiota samples collected from the study subjects’ faces was performed using the kit QIAamp PowerFecal DNA kit (Qiagen) according to recommendations of the manufacturer.

The microorganisms studied are part of the natural skin microbiota. All the human material, such as the skin surface cells, were ignored and not used for analysis, under the responsibility of Gentros, a partner company, specialist in metagenomic analysis.

After the extraction, the DNA was quantified by fluorimetry with the Equipment Qubit 4 Fluorometer – ThermoFischer Scientific.

The quantification result can be seen in appendix 9, table 171 with the extraction samples (microorganisms DNA). These samples were stored by Gentros until the moment of submission to the sponsor under controlled conditions (at 4ºC). The samples was submitted to the sponsor, in Belgium for analysis of skin microbioma. No type of human material was sent for the sponsor.

The samples were discarded right after analysis according to Resolution 441 of the National Health Council (Conselho Nacional de Saúde).

There was no analysis of the subjects genetic material (DNA).

ALLERGISA

## 1.1. Procedure Schedule

Table 5: Study schedule

| | T0 | T2w | T4w | T8w | T12w |
|---|---|---|---|---|---|
| Signature of the Informed Consent Form and Informed Consent For Image Release | X | - | - | - | - |
| Dermatological Clinical Assessment for confirmation of the inclusion and non-inclusion criteria (T0) and safety assessment (T2w / T4w / T8w / T12w) | X | X | X | X | X |
| Assessment of the acne degree (IGA) and assessment of the clinical efficacy by a dermatologist | X | X | X | X | X |
| Acne lesions count by a trained technician | X | X | X | X | X |
| Skin oiliness measurements through the equipment Sebumeter | X | X | X | X | X |
| Facial images capture with the device Visia CR®, for the record and analyses of:<br>- Pores<br>- Porphyrins<br>- RBX (redness of inflammatory lesions)<br>- RBX (recover of the skin barrier) | X | X | X | X | X |
| Skin self-grading (SFG) by the subjects | X | X | X | X | X |
| Self-Assessment questionnaire (SAS) by the subjects | - | X | X | X | X |
| Microbiota Collection by non-invasive Method | X | X | X | X | X |
| Distribution (D) / Return (R):<br>• Investigational Products<br>• Diary of Product Use | D | - | - | R | - |
| Assessment of product acceptance and compliance by checking Daily Log | - | X | X | X | - |
| Investigational products weighing | X | - | - | X | - |
| Assessment of Adverse Events (if applicable) | - | X | X | X | X |

## 8.5. Criteria and Procedures for Study Subjects Withdrawal

The exclusion of a study subject by the investigator could have occurred due the following reasons:

- Study subjects not included: subjects who signed the ICF, but who did not meet the inclusion and exclusion criteria of the study;
- Subjects who present - at the Investigator's discretion - any problem that would prevent the product application from continuing, at any time during the study;
- Consent withdrawal by the study subject, regardless of the reason;
- Lack of adhesion of the study subject to the study. A significant lack of adhesion will be recorded if the subject does not visit the study center for assessments;
- Serious Adverse Event;

- Concurrent disorder or treatment: any pathological process or treatment that occurs during the study period and that might interfere with the study product, such as a medication interaction or masking of results.

Those subjects removed from the study by the investigator would be assessed in case they present any event possibly related to the study, even after their removal. Those subjects removed due to occurrence of an adverse event would be continually assessed until the case is completely resolved.

Those subjects who are removed from study after the inclusion stage were not replaced.

## 9 ADVERSE EVENTS

An adverse event is any untoward medical occurrence in a patient or clinical investigation subject administered a product and that does not necessarily have a causal relationship with this treatment. An adverse event can therefore be any unfavorable and unintended sign (including an abnormal laboratory finding), symptom, or disease temporally associated with the use of the investigational product (adapted from ICH, 1996).

According to the Good Clinical Practices (ICH, 1996), a Serious Adverse Event is any untoward medical occurrence that at any dose:

- Results in death;
- Is life-threatening;
- Requires inpatient hospitalization or prolongation of existing hospitalization;
- Results in persistent or significant disability/incapacity;
- Is a congenital anomaly/birth defect.

Thus any new sign, symptom or disease, or clinically significant worsening compared to the condition at the first visit, should be considered an Adverse Event. Lack of clinical or self-assessment of a cosmetic product or drug is not considered an Adverse Event.

Clinical signs and dermatological or systemic diseases observed during the selection process of the study subjects are not considered as Adverse Events. This information is recorded on the medical assessment form as a reason for non-inclusion and the subjects are then not included in the study.

The adverse events occurred as a result of incorrect product use (either cosmetics or drugs products) - such as inappropriate frequency or incorrect application - will be considered as adverse events that do not interfere with the product evaluation, since the subject- in this situation - does not follow the correct use directions stated on the product label.

An Adverse Event Form is completed for all events occurred. The study sponsor is notified of an adverse event through a Notification of Occurrence form sent by electronic mail or in the Final Study Report.

In case there is an adverse event with doubtful causal nexus, an investigation process is initiated in order to determine if such event is or is not related to the study or investigational product.

The procedures adopted during the event investigation are defined by the physician in charge, based on the nature of the reaction, the subject's medical history and on factors that may interfere with the occurrence of the event, such as medication or other concomitant disorders.

When closing a final diagnosis, the relationship of an Adverse Event to the study or study products can be defined by using one of the following expressions:

For the conclusion of the final diagnosis, the relation of an Adverse Event can be defined using the decision tree Colipa (2016), according to the following description:

- Very likely: Only cases in which the clinical condition is considered to be evocative will be classified as a very likely nexus, the following conditions occurring together: (i) the temporality of the facts is compatible with an adverse reaction to cosmetics and (ii) there is a laboratory test that confirm the relationship with the test product (e.g. positive patch test for the test product).
- Likely: The cases in which the clinical condition is considered to be evocative will be classified as likely causal nexus, occurring with the following conditions together: (i) the temporality of the facts is compatible with an adverse reaction to cosmetics and (ii) there is no laboratory test to confirm the relationship with the test product (e.g. diagnosis of contact dermatitis, without patch test, cosmetic acne - there are no laboratory tests to confirm the relationship with the product).
- Not clearly attributable: Cases in which the clinical scenario is not considered to be evocative or the chronology is not clearly compatible or unknown, will be classified as nexus not clearly attributable.
- Improbable: The following two cases are associated with an improbable nexus: the clinical scenario is not considered to be evocative, the chronology is not clearly compatible or unknown, and the result of the investigation with the test product is negative (patch test or re-exposure).

Excluded: The cases in which the diagnosis corresponds to a dermatosis of well-known cause and / or known to be caused by the use of cosmetics will be classified as excluded nexus (e.g. vitiligo, tineas, pityriasis rosea, pityriasis versicolor, psoriasis, folliculitis, solar melanose, ephelides, among others), when there is no correlation between the subject's complaint and the use of a cosmetic product (for example: muscle pain, lack of appetite, stomach pain, diarrhea, insect bites, among others) or the chronology is clearly incompatible with an adverse reaction to the cosmetic product (for example: there is no improvement in the scenario, even with the interruption of the product; there is relapse of the scenario, without the reintroduction of the product; the signs and symptoms started before the start of the product use).

## 10 STATISTICAL ANALYSIS

Exploratory data analysis was performed (descriptive statistics), according to Guideline for the Statistical Analysis of Efficacy Studies Version 1.4 – October 2018.

The normality in the study was verified with the Shapiro Wilks test with significance level of 1%.

Within one parameter, parametric and non-parametric analysis methods were not be mixed.

The number of subjects in the study was 36 for Study Cream Batch A and 31 for Study Cream Batch B.

Confidence level: 95%

Softwares: XLSTAT 2019 and Minitab 14.

The raw data and statistical analysis were available at Appendix 8.

The detailed statistical approach for each type of data are described below.

## 10.1. Objective parameter (Instrumental analysis – Sebumeter and Visia CR® and Counting of Acne Lesions)

Comparison among treatments, if a baseline measurement at T0 was performed:

*Analysis of the baseline situation (pre-test):*

Comparisons between baseline between treatments were performed through pairwise Student t tests using original data. When normality was rejected, the Wilcoxon Signed rank test was performed. The bilateral hypothesis was used.

*Analysis after application (post-test)*

For each point in time, Student t test for independent samples using the differences to baseline T0 was performed.

Comparison between points in time:

For each treated test site, a repeated measure ANOVA with the factor point in time as qualitative variable was performed using original data. The post hoc pairwise comparisons used was the Fisher's least significant difference (LSD) test.

## 10.2. Ordinal and interval scaled data (Dermatological Clinical Assessment, Investigator Global Assessment (IGA) of acne severity and Self-Grading (SFG))

Comparison among treatments, if a baseline measurement at T0 was performed:

*Analysis of the baseline situation (pre-test):*

Comparisons among treatments at baseline T0 were be performed via Wilcoxon rank sum (Mann-Whitney) tests using original data.

*Analysis after application (post-test)*

For each point in time, comparisons among treatments were performed via Wilcoxon rank sum (Mann-Whitney) tests using differences to baseline T0.

Comparison between points in time:

For each treatment, comparison among points in time were performed via pairwise Wilcoxon's signed rank tests using original data.

ALLERGISA

### 10.3. Nominal data (Self-Assessment (SAS))

In case of the yes/no scale with “do not know” only the agreement level is of particular interest. Therefore, it is sufficient to represent only the agreement level in the results section. More detailed results like the absolute and relative frequencies for all categories ”yes”, “no” and “do not know” were presented in the appendix 8.
