# Supplementary material for: Selective targeting of skin pathobionts and inflammation with topically applied lactobacilli
Source: Cell Rep Med. 2022 Feb 15;3(2):100521. doi: 10.1016/j.xcrm.2022.100521 (PMC8861818; doi:10.1016/j.xcrm.2022.100521)
Supplement: Document S2. Article plus supplemental information [file mmc2.pdf]

Cell Reports
Medicine

Article

Selective targeting of skin pathobionts and inflammation with topically applied lactobacilli

Graphical abstract

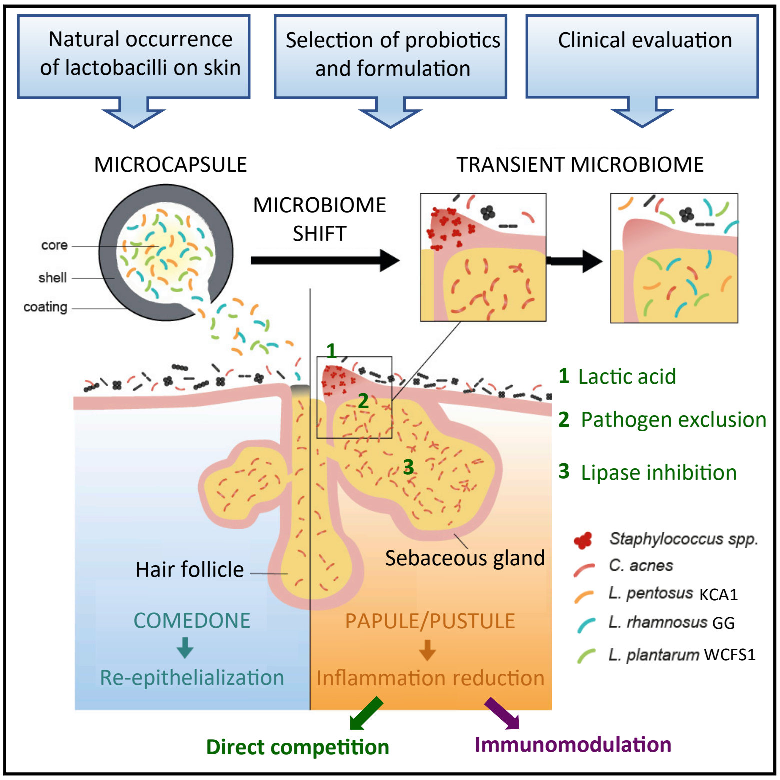

Highlights

- Lactobacilli are underestimated members of the skin microbiota
- Using lactobacilli in topical formulations is challenging but promising
- Lactobacilli can reduce acne lesions after daily application on the skin

Authors

Sarah Lebeer, Eline F.M. Oerlemans, Ingmar Claes, ..., Camille N. Allonsius, Filip Kiekens, Julien Lambert

Correspondence

sarah.lebeer@uantwerpen.be

In brief

In their paper, Lebeer et al. describe that lactobacilli naturally occur on the skin and can be useful as probiotics for the skin. They carefully selected lactobacilli for microencapsulation and use in a topical cream and demonstrated its ability to modulate the skin microbiome and reduce acne symptoms.

Lebeer et al., 2022, Cell Reports Medicine *3*, 100521
February 15, 2022 © 2022 The Authors.
https://doi.org/10.1016/j.xcrm.2022.100521

CellPress

Cell Reports Medicine

CellPress
OPEN ACCESS

Article

Selective targeting of skin pathobionts and inflammation with topically applied lactobacilli

Sarah Lebeer,[1,4,5,*] Eline F.M. Oerlemans,[1,4] Ingmar Claes,[1,4] Tim Henkens,[2] Lize Delanghe,[1] Sander Wuyts,[1] Irina Spacova,[1] Marianne F.L. van den Broek,[1] Ines Tuyaerts,[1] Stijn Wittouck,[1] Ilke De Boeck,[1] Camille N. Allonsius,[1] Filip Kiekens,[2] and Julien Lambert[3]

[1]University of Antwerp, Department of Bioscience Engineering, Groenenborgerlaan 171, B-2020 Antwerp, Belgium
[2]University of Antwerp, Department of Pharmaceutical, Biomedical and Veterinary Sciences, Laboratory of Pharmaceutical Technology and Biopharmacy, Universiteitsplein 1, B-2610 Wilrijk, Belgium
[3]University Hospital Antwerp/University of Antwerp, Department of Dermatology and Venereology, Wilrijkstraat 10, 2650 Edegem, Belgium
[4]These authors contributed equally
[5]Lead contact
*Correspondence: sarah.lebeer@uantwerpen.be
https://doi.org/10.1016/j.xcrm.2022.100521

SUMMARY

Tailored skin microbiome modulation approaches with probiotics are highly challenging. Here, we show that lactobacilli are underestimated members of the skin microbiota. We select specific strains of nomadic lactobacilli for their functional applicability on the skin and capacity to inhibit growth and inflammation by skin pathobionts. The strains are formulated as microcapsules for topical formulations and tested in patients with mild-to-moderate acne. The selected lactobacilli are able to reduce inflammatory lesions in a pilot and placebo-controlled study. Daily application for 8 weeks is associated with an *in vivo* temporary modulation of the microbiome, including a reduction in relative abundance of staphylococci and *Cutibacterium acnes*, and an increase in lactobacilli. The reduction in inflammatory lesions is still apparent 4 weeks after the topical application of the lactobacilli ended, indicating a possible additional immunomodulatory effect. This study shows that carefully selected and formulated lactobacilli are a viable therapeutic option for common acne lesions.

INTRODUCTION

Being the most extensive interface of the human body with the environment, the skin acts as a home to an important part of our commensal microbiota. Similar to the gut, the skin microbiome has essential roles in the education of our immune system and the protection against invading pathogens and other foreign substances. With recent advances in DNA sequencing approaches, our knowledge has improved on the biogeography of the skin microbiota at different body sites.[1] We are now transitioning from these descriptive, observational studies toward a better understanding of the functional roles of the commensal microbiota, allowing the design of tailored modulation approaches. Compared with the richer environment of our intestines, the skin lacks many nutrients beyond basic proteins and lipids, with sweat, sebum, and the *stratum corneum* being main resources.[2] In addition, the skin is a cool, acidic, and desiccated environment, and skin cells are frequently renewed and shed, so that strategies targeting the skin microbiome are highly challenging. Probiotics are live micro-organisms that, when administered in adequate amounts, confer a health effect on the host.[3] They are generally applied in the gut, but the definition also holds true for skin applications, although other terms such as live biotherapeutic products or bacteriotherapy appear to be more preferred.[4] Such strategies have not yet widely been considered for direct application on the skin, in a large part because of the technical challenges.[5]

One of the most common skin diseases is acne vulgaris, a chronic inflammatory skin condition of the sebaceous follicles and glands. The pathogenesis of acne vulgaris is multifactorial, with increased sebum production, alteration in the quality of sebum lipids, dysregulation of the hormone environment, and follicular hyperkeratinization as contributing factors. In addition, specific strains of the facultative anaerobe *Cutibacterium acnes* (formerly known as *Propionibacterium acnes*[6]) are involved in the inflammation of the skin, especially by secreting lipase enzymes that are able to metabolize sebum into free fatty acids, which may lead to skin irritation.[7] Yet, the observation that almost all adults are colonized with *C. acnes* but only a minority have acne, highlights that other bacteria such as *Staphylococcus* species can be linked to acne pathogenesis as pathobionts or disease modulators.[8] Therefore, both oral and topical antibiotics such as doxycycline, minocycline, and clindamycin are frequently used by acne patients,[9] often also for their

This is an open access article under the CC BY license (http://creativecommons.org/licenses/by/4.0/).

anti-inflammatory effects. However, because of the rising problems of antibiotic resistance, alternative therapies need to be developed.[10]

Here, we explored the potential of topically applied live lactobacilli to beneficially modulate cutaneous microbial interactions and host inflammatory responses in subjects with mild-to-moderate acne. Although lactobacilli have a long history of safe use in fermented foods,[11] the gastrointestinal tract,[12] urogenital tract,[13] and nasal cavity,[14] it was not certain whether lactobacilli could also thrive and have health-promoting activities on the skin and whether they could be developed in a skin cream in viable state.

RESULTS

Detection of lactobacilli in skin microbiome samples

Because lactobacilli are not considered to be commensals of the skin, we first monitored the prevalence and relative abundance of lactobacilli on the skin of healthy volunteers. Their relative abundance was explored through 16S amplicon sequencing via Illumina MiSeq (separate runs for V1V2 and V4 variable regions) of facial skin samples (cheek). Thirty volunteers (15 male and 15 female, age ranged from 25 to 64 years old, median: 26), who did not display acne-related lesions, were included. In the samples of all female volunteers and 12 male volunteers, sequences classified as lactobacilli were found. They generally did not occur in the top five of most abundant taxa present on the skin. However, some volunteers showed a relative high abundance of lactobacilli taxa (amplicon sequence variants or ASVs), up to 6.4% (based on V1V2 16S sequencing) or 14.3% (by V4 16S sequencing) (Figures 1A, S1A, and S1B). The relative abundance of these taxa based on both runs (V1V2 and V4) was also 10-fold higher in women compared with men, with an average relative abundance of 0.8% (1.4% in women and 0.2% in men, Kruskal-Wallis $p = 0.0005$; Figure S1B). Lactobacilli-related taxa could thus be potential endogenous members of the skin microbiota, although their relative abundance is lower than that of *Staphylococcus*, *Corynebacterium, Cutibacterium* (often still classified as *Propionibacterium*), and *Streptococcus*, which were the most dominant taxa in our dataset for both variable regions sequenced (Figure S1A).

Nevertheless, skin microbiome samples are low in bacterial biomass and thus prone to contamination, either *in vivo* (e.g., when a healthy volunteer touches his/her face after having touched body sites or fermented foods rich in lactobacilli) or during the wet-lab procedure preparing the amplicon sequencing (e.g., from previous runs for vaginal microbiome samples). Therefore, to confirm our in-house-generated data and investigate whether our results were facial site-specific, the presence of lactobacilli was also substantiated in publicly available skin metagenome shotgun datasets by using the curatedMetagenomicData R-package.[20] In total, 512 samples from six different skin studies[15–18,21,22] were analyzed. Of these samples, 38% (197/512) showed the presence of at least one species of lactobacilli, but only 36 skin samples showed a relative abundance higher than 1%. Yet high relative abundances up to 52% on the skin were also observed (average relative abundance based on curatedMetagenomicData was 0.05%; Figure 1A). We further included 16S amplicon data from the Human Microbiome Project (V3-V5),[15] where a similar prevalence of lactobacilli (36%, 677/1881 samples) and relative abundance of 0.1% were observed (Figure 1A). The relative abundance of sequences of lactobacilli in the skin samples was also compared with the publicly available data of other human body sites (both 16S and shotgun metagenomes from curatedMetagenomicData; Figure 1A). As expected, the vagina showed the highest relative abundance of lactobacilli-related taxa, but the skin turned out to be the second most important niche for these taxa.

Moreover, to have a better idea of the phylogenetic diversity of all lactobacilli present on the skin, we plotted all data on a phylogenetic tree of the Lactobacillaceae[23] (as recently taxonomically redefined) (Figure 1B). These data indicate that taxa typically associated with the human vagina, *Lactobacillus crispatus*, *L. iners*, *L. gasseri*, and *L. jensenii* were also found as the most prevalent lactobacilli on the skin (Figures 1C and 1D). Also, nomadic or free-living lactobacilli,[19] namely species from *Latilactobacillus* (previously the *Latilactobacillus sakei* group), *Lactiplantibacillus* (previously *Lactobacillus plantarum* group), and *Lacticaseibacillus* (previously *Lactobacillus casei* group) were frequently detected (Figures 1C and 1D). The occurrence of lactobacilli on the skin is in agreement with the fact that after normal delivery through the birth canal, these bacteria originating from the vagina of the mother are among the first to colonize neonate skin.[24] The data presented here (Figures 1A–1D) indicate that lactobacilli are still present in adults but do not stay dominant in the different human body skin sites studied. The detection of some nomadic lactobacilli on the skin suggests that some lactobacilli on the skin could also result from fermented food sources and thus be transient passengers. Yet their consistent presence and relative abundance between 0.05 and 1% in our own data and the publicly available data, did lead us to postulate that they could play a role as keystone microbes, recently redefined as taxa exerting a considerable influence on microbiome structure and functioning irrespective of their abundance across space and time.[25] Therefore, we subsequently aimed to manipulate biotic interactions of lactobacilli on the skin by supplementing the endogenous lactobacilli with well-selected potential probiotic lactobacilli.

Rationale for *in vitro* strain selection

Various strains of the Lactobacillaceae were selected from our in-house available laboratory collection (Table S1) for tailored application on the skin. A thorough screening approach was applied based on the rationale that the strains had to be safe, be applicable (being robust and showing lifestyle flexibility, as described for lactobacilli by Duar et al.[19]), and have the capacity to exert beneficial functions on the human skin, including microbiome modulation, immune modulation, and epithelial barrier enhancement (Figure 2A). Key properties were substantiated with laboratory tests, genome screening, and information available in the literature. Three strains were selected, i.e., *Lacticaseibacillus rhamnosus* GG, *L. plantarum* WCFS1, and *Lactiplantibacillus pentosus* KCA1. The rationale for these strains was based on their genome availability,[26–28] knowledge of their epithelial interaction capacity,[29,30] and their robustness and growth capacity (Figure S2A), in addition to information on their

CellPress
OPEN ACCESS

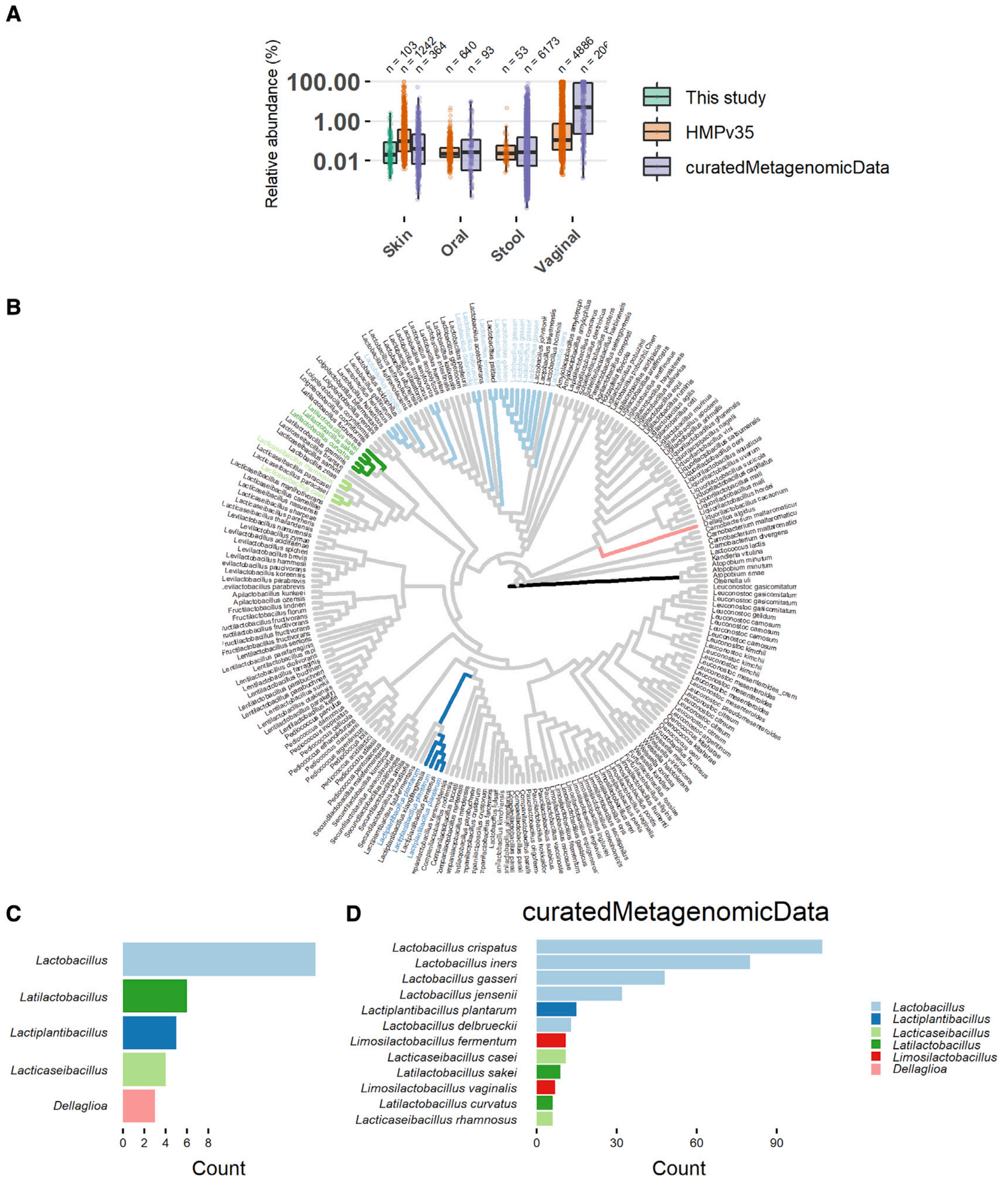

Figure 1. Taxa of lactobacilli found in skin samples of 16S rRNA amplicon and shotgun metagenomic data

(A) Comparison of relative abundance of lactobacilli in different niches in the proof-of-concept study (merged two technical replicates), the Human Microbiome Project (HMPv35)[15] and shotgun metagenomic datasets from three studies,[16–18] accessed through the curatedMetaganomicData R Package. The y axis is represented in log scale.)

(B) 16S rRNA cladogram of the Lactobacillaceae. Branches are colored based on phylogenetic placement of the ASVs of lactobacilli from this study and the phylogenetic group (as described by Duar et al.[19]) that they belong to. Tip labels are colored based on the 12 most abundant species of lactobacilli found in the skin shotgun metagenomic datasets.

(C and D) The most abundant lactobacilli in this study (C) and the skin shotgun metagenomic datasets (D) colored according to the phylogenetic group they belong to

safety in humans after oral,[31–33] nasal,[34] and vaginal[35] high-dose application. *L. rhamnosus* GG was also selected because of previous reports on its capacity to inhibit the toxic effects of *Staphylococcus aureus* on epidermal keratinocytes,[36] its strain-dependent capacity to promote re-epithelialization via secreted proteins such as Msp1/2 (p40/p75),[37] and to augment tight-junction barrier function in human primary epidermal keratinocytes[38] and because of our previous experience with this probiotic

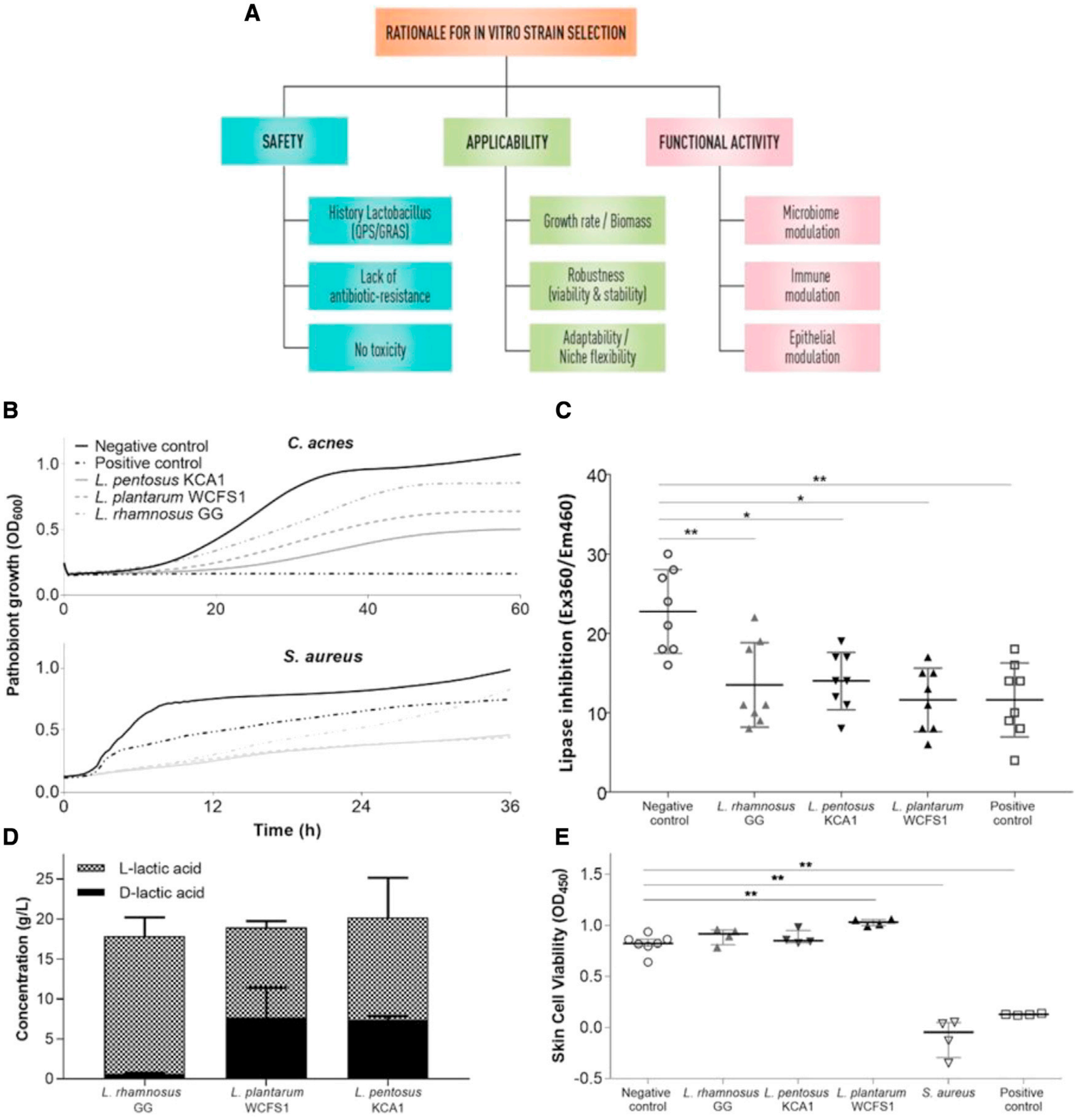

Figure 2. ***In vitro*** **selection of lactobacilli for targeted application against acne vulgaris**

(A) Schematic overview of the rationale for the selection. Each criterion needs to be taken into account upon selection. Laboratory or genomic prediction tests exist for each criterion. More information can be found in the main text.

(B) Antimicrobial activity of the spent-culture supernatant of the selected strains against the two pathobionts tested, *C. acnes* and *S. aureus*, and compared with the positive control (10 mg/mL Clindamycin, a common antibiotic used in acne). MRS at pH4, which is comparable to the pH of the spent supernatant of lactobacilli, was used as a negative control. Three technical replicates were combined to create the graphs.

(C) Inhibition of lipase activity of *C. acnes* by the spent-culture supernatant of the selected *Lactobacillus* strains and compared with the positive control (10 mg/mL Clindamycin) and the negative control (MRS). Each shape represents one technical replicate (means +/- SD).

(D) Concentration of L-lactic acid and D-lactic acid as key antimicrobial and skin-modulating molecules produced by the selected lactobacilli after overnight incubation in MRS broth. Each bar graph results from at least three technical replicates (means +/- SD).

(E) Skin cell viability results of normal human epidermal keratinocytes after addition of the selected lactobacilli compared with the negative control keratinocyte growth medium 2, and positive controls, *S. aureus* and Triton X-, measured at 450 nm using an XTT assay. Each shape represents one biological replicate. Medians with interquartile ranges are also shown. Statistical analysis was performed using a Mann-Whitney test, where *$p < 0.05$ and **$p < 0.01$

strain.[29] For microbiome modulation, *C. acnes* was targeted as a model pathobiont associated with the inflammatory character of acne vulgaris. *S. aureus* was also targeted as an important pathogen causing skin inflammation.[39]

When the activity of spent culture supernatant of our collection of lactobacilli was screened for antimicrobial effects on the growth of *C. acnes* in suspension, all strains tested inhibited the growth of *C. acnes* ATCC6919 and *S. aureus* ATCC29213,

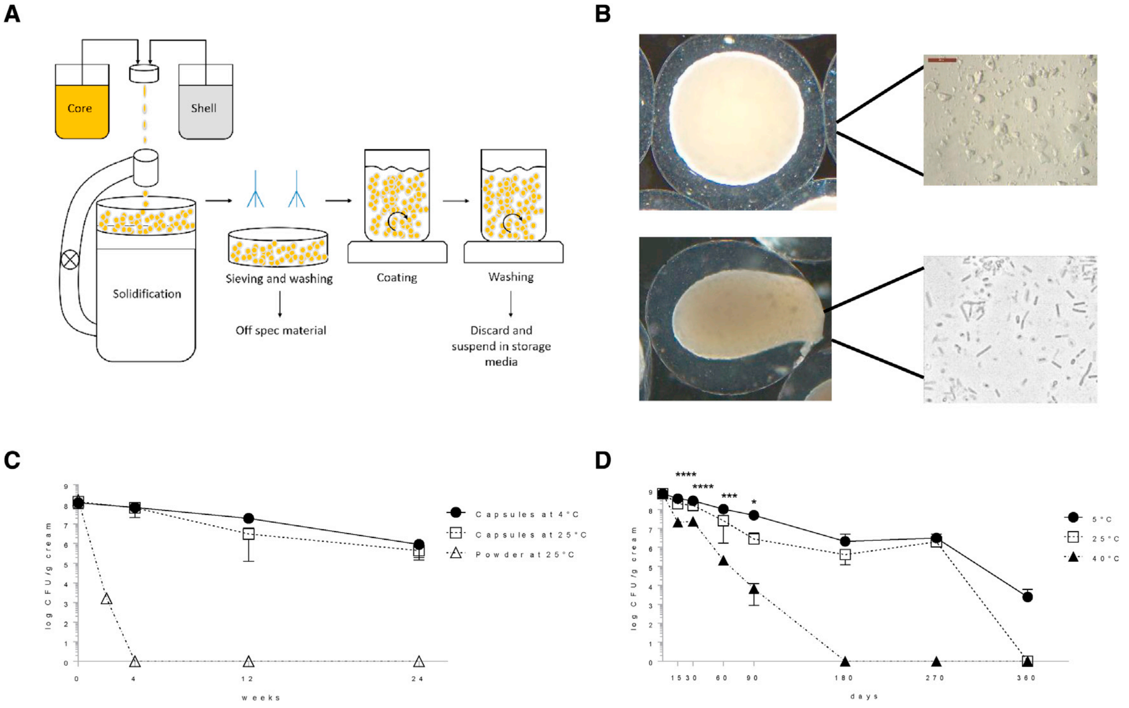

Figure 3. Formulating live lactobacilli in a topical cream

(A) Schematic representation of the micro-encapsulating process with the bacteria in the core suspension and an outer shell made by the shell solution.

(B) Resulting microcapsules with a core of freeze-dried bacteria suspended in oil compared with microcapsules in which a force is applied just before application on the skin, thereby releasing the bacteria and activating them through water uptake.

(C) Survival of encapsulated bacteria, suspended in an O/W cream, compared with the non-encapsulated freeze-dried bacterial powder in the cream. Results are shown as mean with SD.

(D) Survival of the encapsulated bacteria in O/W cream tested according to the International Council for Harmonisation of Technical Requirements for Pharmaceuticals for Human Use Q1A(R2). Results are shown as mean with SD. Statistical analysis was performed using a two-way ANOVA, where *$p < 0.05$, ***$p < 0.001$, and ****$p < 0.0001$.

but *L. pentosus* KCA1 (vaginal origin) and *L. plantarum* WCFS1 (saliva origin) were among the strains tested able to exert the highest inhibition (Figures 2B and S2B). Other related strains tested, including *Staphylococcus epidermidis* 12,228, did not inhibit *C. acnes* growth (Figure S2). In addition, the selected strains of lactobacilli were able to significantly reduce the lipase activity of *C. acnes* (Figure 2C). These lipase enzymes are involved in inflammation of the skin induced by *C. acnes*, because they metabolize sebum into free fatty acids, which may lead to skin irritation.[7] Furthermore, because lactic acid has a strong antimicrobial activity,[40] it may be involved in maintaining the anti-inflammatory status of the skin and has a documented dose-dependent capacity to ameliorate the appearance of acne in dermatology.[41] We therefore substantiated lactic acid production by the selected lactobacilli (Figure 2D). We also validated that the three selected lactobacilli did not exhibit toxic or overt inflammatory responses on primary skin cells (Figure 2E), in agreement with genome predictions,[26–28] and also lacked known antibiotic-resistance genes (Resfinder[42]) on mobile elements and known virulence genes.[43] Laboratory validation of antibiotic-resistance profiles according to the guidelines of the European Food Safety Authority (EFSA)[44] confirmed a phenotypic lack of antibiotic resistance of concern. Furthermore, these assays confirmed that standard beta-lactam antibiotics could be used as fallback scenario in the (unlikely) event of a bacteremia when applied on the skin of patients. The fact that these properties can be checked on the basis of comparative genome analyses is a clear advantage that individual well-known strains have over undefined microbial mixtures such as fecal microbiota transplants, for which fatal cases were reported recently by the transfer of drug-resistant bacteria.[45]

Viable probiotic formulation in O/W cream

We then aimed to design a topical formulation suitable for the application of live bacteria in a sufficient dose on the skin. The selected bacteria were freeze-dried for stability reasons[46] and embedded in the core of two-compartment microcapsules (Figure 3A). Various processing conditions were optimized as described in the STAR Methods section and schematized in Figure 3A, resulting in capsules of 1,500–2,000-μm diameter with a core of suspended freeze-dried bacteria that could be released upon application of mechanical pressure, such as rubbing on the skin (Figure 3B). Ingredients were selected so that they did not significantly impact on the growth capacity of the encapsulated bacteria after release, skin commensals, and pathobionts (tested for *Staphylococcus epidermis*, *L. crispatus*, and *S. aureus*) (Figures S2C and S2D). This formulation and encapsulation approach significantly improved the

viability for storage at 4°C and even at 25°C compared with non-encapsulated freeze-dried bacteria when suspended in a carrier oil-in-water (O/W) cream (Figure 3C) and this for up to 9 months (Figure 3D).

Subsequently, the skin irritation potential was checked on 20 healthy volunteers with skin patch tests according to Basketter et al.[47] No erythema, dryness, or edema was observed in any of the volunteers studied (skin irritation index: 0.00) (Table S2). For comparison, adapalene products, which are naphthoic acid derivatives with retinoid activity and documented efficacy in the treatment of mild-to-moderate acne vulgaris, have a mean cumulative irritation index in healthy subjects with normal skin between 0.25 and 1.[48] Also, the widely used combined clindamycin-benzoylperoxide treatment for moderate acne has been reported to frequently induce dry skin, flaky/peeling skin, irritated skin, itchy skin, and redness in acne patients.[49]

Skin microbiome modulation with live lactobacilli

Subsequently, we applied the topical cream in an open-label "proof-of-concept" longitudinal trial. Ten volunteers applied the cream with 10^8 CFU of live lactobacilli per application (±1 g/application) for 8 weeks twice daily. Samples were taken before, during, and 2 weeks after the intervention (Figure 4A). Patients with mild-to-moderate acne symptoms that were not using antibiotics or another acne treatment were included by the responsible dermatologist (Table S3). The impact of the cream with lactobacilli on their facial skin microbiome was monitored by 16S amplicon sequencing of the V1 and V2 hypervariable regions at four different time points over a period of 10 weeks (Figure 4A). In this way, the skin baseline microbiome before, during, and after the treatment was compared. The skin acne microbiome of these patients at the time of inclusion was especially characterized by an increased relative abundance of *Staphylococcus* taxa (p = 0.0058, Wilcoxon rank sum test) compared with the healthy controls (Figure 4B) (Figure S3 for three specific *Staphylococcus* ASVs). After application of the cream with the lactic acid-producing lactobacilli, the facial skin samples of our acne patients at visit 2 (4 weeks) and visit 3 (8 weeks) clearly clustered separately on a PCOA plot (Figure 4C). Interestingly, in seven of 10 patients at visit two and eight of 10 patients at visit 3, the ASVs for lactobacilli were found in high relative abundances (between 20.9% and 92.8%), whereas in three patients at visit two and two patients at visit 3, their relative abundance was below 5% (between 0.015% and 1.1%; Figure 4D), with also a significant correlation between relative abundance of lactobacilli and comedones at visit 2 (Figure S4C). ASV analysis via the EzBioCloud database[50] and comparison with the whole-genome sequences[26–28] for rRNA copy variants confirmed that the detected ASVs matched the applied lactobacilli. Interestingly, the three probiotic strains appeared to persist on the skin in similar numbers (Figure 4D). To substantiate that the lactobacilli detected on the skin were still viable, samples were also plated on selective MRS agar for lactobacilli. Most samples at visit 2 (7/9) and visit 3 (6/7) were culture positive, indicating that (at least some of) the applied lactobacilli were metabolically active on the skin (Figure 4D). At visit 4 (2 weeks after the cessation of the treatment), most ASVs for lactobacilli had disappeared, and growth in MRS medium was also markedly reduced, further substantiating that the lactobacilli detected originated from the applied topical cream, although the (endogenously present) *L. delbrueckii* group (*Lactobacillus* genus *strictu sensu*) could still be detected. We then explored whether the presence of lactobacilli during treatment had impacted on the pathobionts of acne (*C. acnes* and *Staphylococcus* taxa). The relative abundance of both pathobiont taxa had indeed already dropped at visits 2 and 3 and increased again at visit 4 ($p < 0.05$ for visit three versus visit 1; Wilcoxon test for *Staphylococcus*; Figure 4B).

To confirm the observed skin microbiome modulation with live lactobacilli in the pilot open-label study, a double-blind placebo-controlled study was set-up including 79 acne patients with at least nine inflammatory lesions at the time of inclusion (12–33 years, all genders) (Figure 5A). Patients were randomized, and they applied either the active cream with encapsulated lactobacilli or the vehicle placebo, namely the same cream and microcapsules but without lactobacilli as active ingredient. The impact of the cream with lactobacilli on their facial skin microbiome was monitored by 16S amplicon sequencing at five different time points: before, during (after 2, 4, and 8 weeks), and 4 weeks after the intervention (Figures 5A and 5B). Notably, the swabs for these microbiome analyses were taken at least 1 h after the last application. During the intervention study, ASVs of Lactobacillaceae were found in high relative abundances in the verum group (Figure 5C). ASV analysis and comparison with the whole-genome sequences for rRNA copy variants again confirmed that the strains in the cream matched the ASVs in the sequencing data for 100%: *L. rhamnosus* GG matched the ASV *Lactobacillaceae* 1, while *L. plantarum* WCFS1 and *L. pentosus* KCA1 matched the ASV *Lactobacillaceae* 2. A significant increase in relative abundance of taxa of the Lactobacillaceae was observed at 2, 4, and 8 weeks. Of note, their relative abundance also decreased with time, which was in line with the decrease in viability of the lactobacilli in the cream during storage (Figure 5D). This was carefully monitored as part of the quality control and assurance. An increase of *Lactobacillaceae* ASVs was also observed in the placebo/vehicle group, suggesting that the cream formulation might also stimulate the colonization of endogenous lactobacilli on the skin, but this increase showed not to be significant. We then checked the relative abundance of acne pathobionts *C. acnes* and *Staphylococcus* taxa. A significant decrease in relative abundance of staphylococci was observed after 2 weeks of intervention ($p < 0.05$), in line with the highest relative abundance of lactobacilli at these time points (Figure 5D). No major impact on the relative abundance of *Cutibacterium spp.* was seen (Figure S5), although this could also have been due to the 16S rRNA V4 region. This region is not optimal for *Cutibacterium* but was chosen because of the focus on lactobacilli and staphylococci. We also explored whether the application of live lactobacilli has pathobiont-specific or more broad-acting microbiome effects (Figure S6). No significant impact of the lactobacilli intervention was found for the alpha diversity (measured by observed diversity and inverse Simpson index; Figure 6B). On the PCOA plots (Figure 6A) also, no clustering of the skin samples by treatment group or by time point could be observed. These observations are in line with specific effects of the lactobacilli cream on specific skin taxa without having a disrupting the microbiome balance.

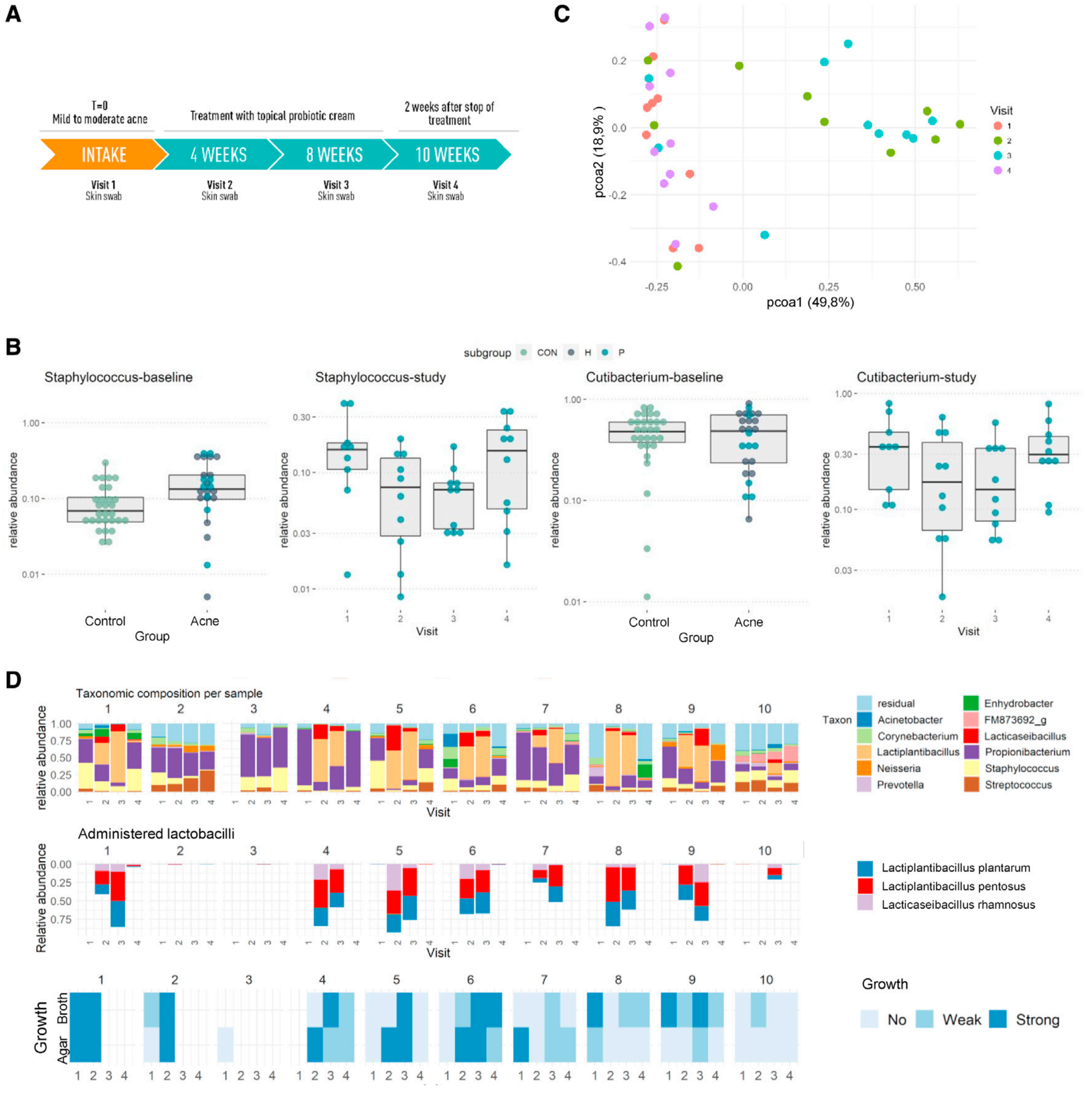

Figure 4. Impact of the lactobacilli cream on the skin microbiome: proof-of-concept study

(A) Schematic overview of the proof-of-concept intervention study with the O/W cream containing the selected and formulated lactobacilli, the visits at which a skin swab was taken, and dermatological symptom analysis performed by the dermatologist. The cream was applied twice daily for 8 weeks with a minimal dose of 106 CFU per application.

(B) Relative abundance of *Staphylococcus* and Lactobacillales respectively at baseline (left) and over the four visits of the study (right), resulting from 16S sequencing of the V1-V2 hypervariable regions. For the baseline, skin samples of the 30 healthy volunteers without acne symptoms (see Figure 1) and 27 patients with mild-to-moderate acne symptoms were compared. Of these 27 acne patients, 10 patients (indicated with light blue dots) were included in the intervention study with lactobacilli (Study) shown at the right side of each panel. Significant differences, as indicated by a pairwise Wilcoxon test with Holm correction for multiple testing, are indicated as $^{**}p \leq 0.01$ and $^{***}p \leq 0.001$.

(C) PCOA plot distributing samples according to beta-diversity (Bray-Curtis distance). While the first dimension (x axis) was able to capture 49.8% of variation in the samples, a second dimension (y axis) shows 18.9% of variation. Similar samples are located closely to each other and colored by visit.

(D) Microbial communities during the study period with the genus-level taxonomy indicated (top), relative abundance of the three ASVs resulting from the lactobacilli in the cream (middle), and observed growth on MRS medium (top row on agar [A], bottom row growth in MRS broth [B]) after addition of the skin samples (bottom). Other lactobacilli ASVs were not observed at a higher relative abundance than 1%. Samples were ordered by participant and by visit. For B and C, each sample (or dot) represents two merged technical replicates (if both passed QC).

Lactobacilli-mediated improvement of acne symptoms

Subsequently, since the acne pathogenesis is more than pathobiont overgrowth[51] and probiotic effects often include anti-inflammatory effects at other body sites,[52] other effects of the lactobacilli cream were evaluated. Acne lesions were clinically scored as the presence of inflammatory lesions and comedones. This analysis showed an overall improvement of the acne lesions in the patient group of the open-label pilot study

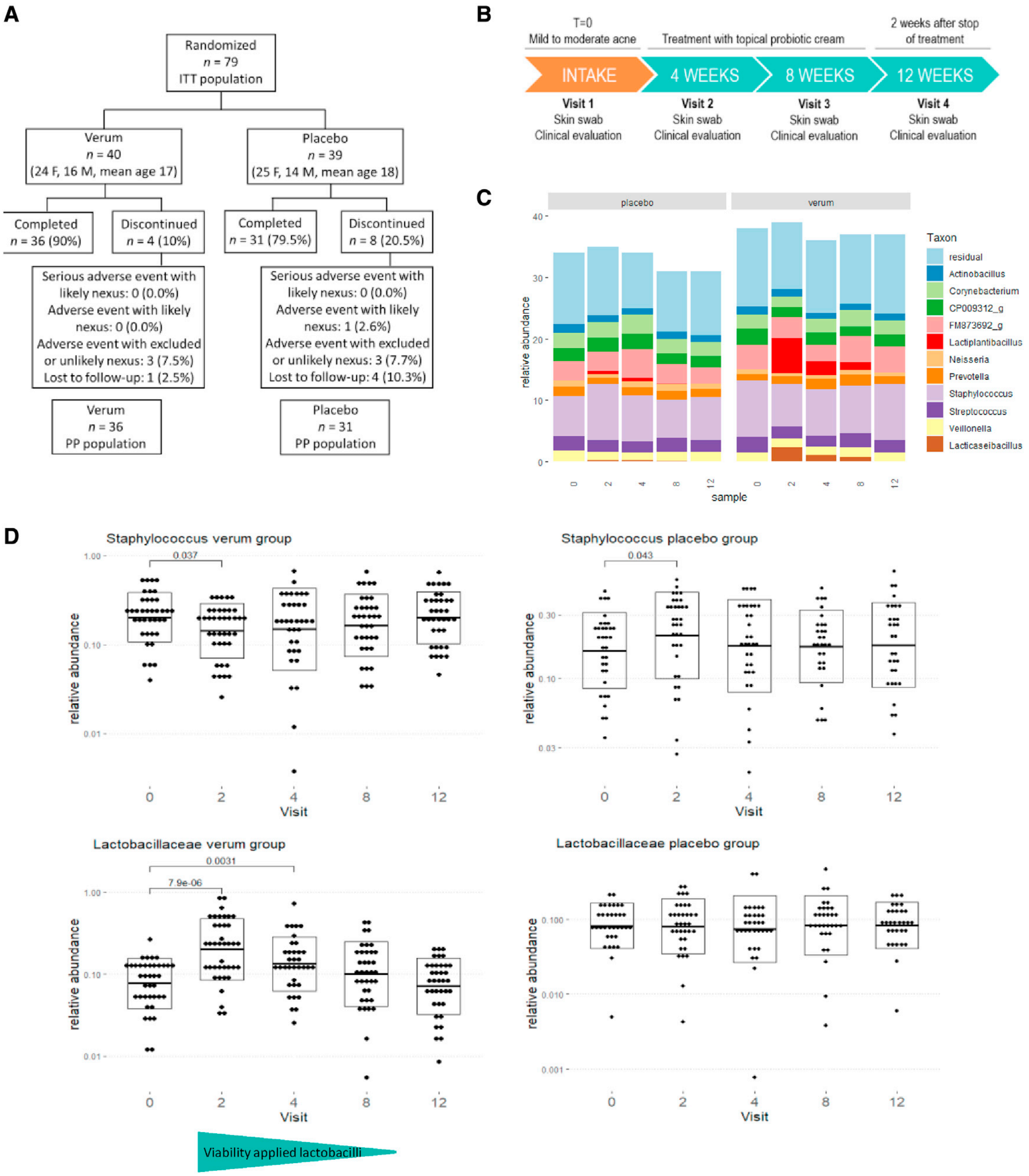

Figure 5. Impact of the lactobacilli cream on the skin microbiome: DBPC study

(A) Flowchart of double-blind placebo-controlled (DBPC) study detailing enrollment and adverse events.

(B) Schematic overview of the DBPC intervention study. The cream (verum or active) was applied twice daily for 8 weeks with a minimal dose of 10^6 CFU per application for the verum cream.

(C) Summary of microbial communities during the study period with the genus-level taxonomy indicated. Other *Lactobacill*aceae ASVs were not observed at a higher relative abundance than 1%. Samples were ordered by participant and by visit.

(D) Relative abundances of *Staphylococcus* genus and *Lactobacillaceae* family (former *Lactobacillus* genus complex) during the study period, as found through 16S sequencing of the V4 hypervariable region.

(12–25 years, male) treated with the lactobacilli-supplemented cream, as reflected by a significant reduction in inflammatory lesions already at visits 2 (T [4 weeks]) and 3 (T8w [8 weeks]) compared with visit 1 (T0) and a significant reduction in comedone counts at visit 2 (Figure 7A). More importantly, the double-blind placebo group could confirm that the lactobacilli played a key role in the observed clinical effects of the pilot study. The patients who topically applied the live lactobacilli (n = 36 completed the trial) showed a significantly higher percentage of reduction in inflammatory lesions than the placebo

CellPress
OPEN ACCESS

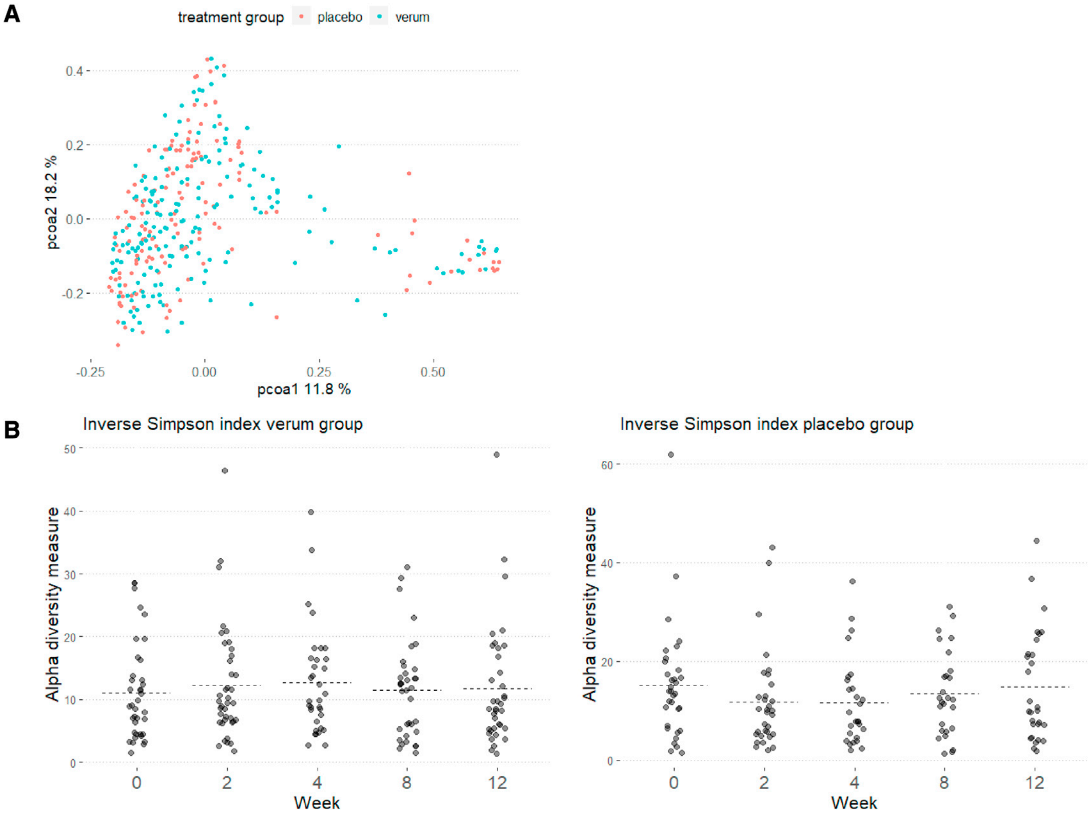

Figure 6. Impact of the lactobacilli cream on the skin microbiome diversity during the DBPC study
(A) PCOA plot distributing samples according to beta-diversity (Bray-Curtis distance). Samples are colored by week of visit. Similar samples are located close to each other.
(B) Alpha -diversity as indicated by Inverse Simpson Index (richness and evenness) and observed diversity (richness) throughout the study period, grouped by week of visit.

group (n = 31 completed the trial) after 4 weeks (34.4% versus 1.7%), 8 weeks (13.7% versus 9.2%), and 12 weeks (22.1% versus −7.9%). These data indicate a rather fast-acting activity, since topical acne products often show significant differences from vehicle only after eight or 12 weeks of treatment.[53] Most remarkably, the lactobacilli were able to maintain the reduction in inflammatory lesions after the treatment had been stopped, pointing toward a possible immunomodulatory effect involving the adaptive immune system. In addition, based on clinical and subjective signs, this study confirmed the exceptionally low irritation potential of both verum (2.8% with mild erythema at 4 weeks, 2.8% very mild scaling at 4 and 8 weeks) and placebo cream (3.2% with very mild scaling at 8 weeks, 3.2% with very mild itching at 12 weeks) (Tables S4 and S5). These data, with no adverse events reported, highlight that the topical cream also has a high safety profile in addition to its efficacy.

DISCUSSION

Acne vulgaris is a common reason for long-term antibiotic use, with dermatologists prescribing antibiotics more commonly than any other physician group.[9] Here, we applied a multiphasic and multidisciplinary approach to substantiate that lactobacilli have potential as skin probiotics to target acne. First, we provided detailed information that lactobacilli are members of the human skin microbiota, with relative abundances between those of human vaginal[13] and stool[12] samples. Of interest, phylogenetic placement of the lactobacilli-related sequences detected in our data (ASVs) and public datasets showed that the dominant *Lactobacillus* taxa (*L. crispatus, L. iners, L. gasseri, L. jensenii*) of the vaginal community were also among the most prevalent taxa of lactobacilli of the skin. Previous studies have briefly acknowledged the presence of lactobacilli in the skin microbiota;[54,55] however, such detailed analysis of specific taxa of lactobacilli in the skin niche had not yet been performed. We also noted that the prevalence (presence/absence) of *Lactobacillaceae* taxa in our dataset was higher than in the Human Microbiome Project dataset. This is probably due to the different approaches used, such as shotgun sequencing, which can have a bias against lower abundant taxa. This is further supported by the fact that the relative abundance of *Lactobacillaceae* in all datasets were similar, in line with the fact that our dataset was not notably biased toward *Lactobacillaceae*. Moreover, our detailed phylogenetic analyses of the lactobacilli detected is also of interest in view of the recent taxonomic changes in the *Lactobacillus*

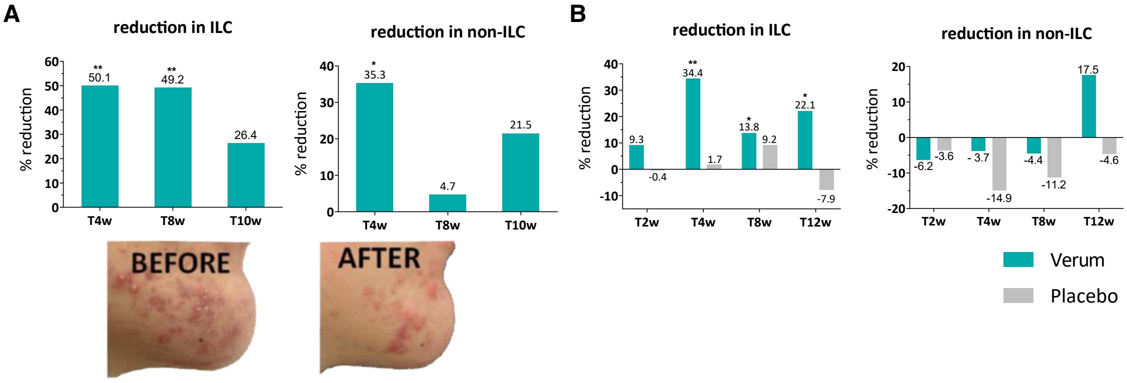

Figure 7. Effect of the lactobacilli cream on acne symptoms

(A) Proof-of-concept study. The percent reduction in inflammatory lesion counts (ILC;, left) and non-inflammatory lesion counts (non-ILC; right) over the course of the study, grouped by visit (averages are shown). All 10 patients included in the pilot study showed a clinical improvement after the application of the cream, as exemplified by a picture of the acne spot area of one patient at T0 versus T4w. Written informed consent was obtained from the patient to use these photographs.
(B) DBPC clinical trial. The percent reduction in ILC and non-ILC over the course of the study, grouped by visit. A significant reduction in ILC was seen at T4w, T8w, and T12w for the verum group and not for the placebo group, which confirms the anti-inflammatory capacity of the live lactobacilli. Averages are shown. Statistical analyses were performed using a Wilcoxon matched-pairs signed rank test, where *$p < 0.05$ and **$p < 0.001$.

genus complex.[23] These changes now allow for more functional studies on the habitat adaptation of specific taxa. Here, we show that the species *L. crispatus*, *L. iners*, *L. gasseri*, and *L. jensenii*, all still belonging to the genus *Lactobacillus strictu sensu*, have a broader human adaptation to stratified epithelium than merely the human vaginal epithelial cells, based on their association found here with (healthy) skin. However, it is possible that lactobacilli on the skin are transient passengers originating from food, the oral cavity, or the vagina, where they frequently occur. Nevertheless, it means the skin is (continuously) exposed to these taxa, and this exposure may have an impact on health, as we also aimed to study in this paper. Indeed, to exert a potential health benefit, the bacteria should not necessarily be endogenous stable members of the microbiota. Therefore, to apply selected lactic acid bacteria on the skin, not only functional properties were considered here, but also technical properties, because host-adapted microbes are often less able to survive and thrive outside the host. Here, we screened for robustness to (processing) stress conditions and good growth capacity, in addition to safety and lack of (transferable) antibiotic resistance properties, lactic acid production, inhibition of lipase activity by *C. acnes*, and growth of *S. aureus* as key probiotic properties (as rationalized in Figure 2A). The inhibition of lipase activity of *C. acnes* by lactic acid-producing lactobacilli had not yet been documented, but is in line with earlier research on the related cheese-associated *Propionibacterium* species that can utilize lactic acid as a nutrient and as an alternative metabolic pathway for lipolysis.[56] Following this mechanistically driven screening, we did manage to translate our results directly from *in vitro* laboratory tests with skin cells and pathogens to human volunteers without the need for animal testing. The selected *L. rhamnosus* GG, *L. plantarum* WCFS1, and *L. pentosus* KCA1 could inhibit the growth of *C. acnes*, and *S. aureus in vitro* could survive the formulation in capsules in an O/W cream and were found in similar amounts on the facial skin of patients with mild-to-moderate acne. Twice daily topical application of this cream with live lactobacilli was able to reduce inflammatory acne lesions and comedone formation in the 10 patients included in an open-label pilot study and was associated with a specific reduction in *Staphylococcus* relative abundance but no overall impact on skin microbiome diversity (as summarized in Figure 7). A follow-up placebo-controlled trial confirmed that the lactobacilli formed the key active ingredient for the reduction of inflammatory lesions. Remarkably, this reduction in inflammatory lesions persisted 4 weeks after treatment cessation. 16S rRNA ASV-based comparison of the facial microbiome of 30 healthy volunteers and 27 patients with acne symptoms suggested indeed that *Staphylococcus* taxa are increased in acne patients and that *Staphylococcus* could thus form an interesting acne target to investigate further. This role of *Staphylococcus* needs more attention in future research on acne, because it is currently somewhat understudied in contrast to other skin disorders such as atopic dermatitis.[57]

ASV-level analysis of the sequenced V4 region of the 16S rRNA gene did not allow identification of the *Staphylococcus* taxa up to species/strain level, so that no distinction between *S. epidermidis* and *S. aureus* and more specific virulent strains should still be made. Yet, our results show that the nomadic or habitat-flexible lactobacilli chosen here from the *L. rhamnosus* and *L. plantarum* group could have long-term immunomodulatory effects against the *Staphylococcus* pathobionts and inflammation associated with acne, in line with the fact that beneficial effects observed in the clinical trial were still being observed 4 weeks after treatment stop. This points toward the stimulation of adaptive immune responses, such as increasing antibody production against the pathobionts, or immunoregulatory mechanisms helping to keep *S. aureus* in check. It will be very interesting in future experiments to explore whether the specifically selected lactobacilli could promote differentiation of a subpopulation of tissue-resident memory T cells (T_{RM}) or other immunomodulatory interactions as has been described previously.[58]

Conclusion

In this study, we show that live lactobacilli have probiotic potential for the skin and especially to reduce inflammatory lesions in acne. As compliance is key in acne management, the relatively fast reduction of the inflammatory lesions is an added value. The randomized placebo-controlled study demonstrated that the live lactobacilli are the key active pharmaceutical (or better pharmabiotic) ingredient responsible for the reduction in acne symptoms. Furthermore, the topical probiotic cream proved to be well tolerated and even to improve moisturization (skin hydration increased 37.3% after 14 days and 45.6% after 28 days of use), which is a unique property in the context of acne. We have performed in this study a detailed microbiological and molecular screening of the lactobacilli before their *in vivo* application on human skin of volunteers and patients, yet we acknowledge that more molecular studies are needed to unravel underlying immunomodulatory mechanisms in relation to the selected live lactobacilli and to translate the *in vivo* findings back to exact mechanisms. Further scrutinization of our results is necessary to contribute to a new era of skin therapeutics based on microbiome modulation as well as more fundamental and mechanistic insights into the possible keystone functions of lactic acid bacteria for skin health.

Limitations of the study

The study represented here uses 16S amplicon sequencing of two regions as main approach for the microbiome analyses. Despite its many advantages, it also has some important limitations. First, although it provides quantitative information in terms of relative abundances, translating these compositional data to absolute abundances remains very challenging. Although qPCR can provide estimations of absolute concentrations, this approach is sensitive to contamination (also the case for amplicon sequencing in low-biomass samples as used here), aspecific binding of primers, suboptimal primer efficiency, and is only applicable to dedicated taxa without providing an overview picture, which is why we have not included that analysis here. Another limitation of 16S amplicon sequencing is that it does not allow taxonomic classification of the detected bacterial variant to species level with the necessary certainty (and therefore uses ASVs), so care should be taken when assigning species. Future work should for example include shotgun metagenomic sequencing to account for this. Furthermore, while we have mainly focused here on the activity of the lactobacilli to directly impact on skin pathogens/pathobionts and the skin microbiota, it is also possible that the lactobacilli indirectly influence the microbiota through immune modulation, as mentioned above. We have not monitored this in this study and therefore cannot provide information on the possible immunomodulatory mechanisms involved. In addition, we did not specifically investigate the (longitudinal) viability and activity of the applied lactobacilli on the skin, e.g., through RNA sequencing or metabolomics. Such approaches are very challenging for the skin. In the proof-of-concept study, we did include culture to show some viability and activity, but this analysis should be further substantiated in the future. Finally, we report two intervention studies with 10 (proof of concept) and 68 participants (RCT of which 31 were allocated to the placebo group), but to detect more subtle changes to the microbiota, larger study groups are needed.

STAR★METHODS

Detailed methods are provided in the online version of this paper and include the following:

- KEY RESOURCES TABLE
- RESOURCE AVAILABILITY
  - Lead contact
  - Materials availability
  - Data and code availability
- EXPERIMENTAL MODEL AND SUBJECT DETAILS
  - Human subjects
  - Sample collection
- METHOD DETAILS
  - Bacterial growth
  - Human skin cell culture
  - Illumina MiSeq *16S rDNA* gene amplicon sequencing
  - Biostatistical and bioinformatics analysis
  - Analysis of public datasets
  - Formulation of lactobacilli in microcapsules and O/W cream
  - Determination of skin hydration (Corneometry)
- QUANTIFICATION AND STATISTICAL ANALYSIS
- ADDITIONAL RESOURCES

SUPPLEMENTAL INFORMATION

Supplemental information can be found online at https://doi.org/10.1016/j.xcrm.2022.100521.

ACKNOWLEDGMENTS

We kindly acknowledge Dr. K. Anukam for providing us *Lactobacillus pentosus* KCA1. We thank Wannes Van Beeck, Dieter Vandenheuvel, Leen Van Ham, and all other members of the ENdEMIC group for their assistance and/or fruitful discussions. We thank S. Van Elslander for help in the design of Figures 2A, 4A, and 6, and A. Simons for the design of Figure 3A. We also greatly thank Dr. E. Pasolli for the assistance with the CuratedMetagenomicData package. This work was funded by grants from the Flanders Agency for Innovation & Entrepreneurship (www.vlaio.be/en), including an IWT-SBO project grant (IWT/50052) for the fundamental part of the research and a VLAIO R&D project grant with Yun Probiotherapy NV (formerly known as Axca Bvba) as SME partner for the applied part of the project. Sander Wuyts, Ilke De Boeck and Camille Allonsius held personal PhD grants (IWT-SB 141198, FWO 1S17916N, and FWO 1S03518N, respectively) during this study. S.L. currently holds an ERC Starting Grant Lacto-Be on habitat flexibility and adaptation of lactobacilli.

AUTHOR CONTRIBUTIONS

S.L., I.C., and J.L. designed the study; J.L. clinically evaluated the patients and collected patient samples in the proof-of-concept study; E.O. prepared the clinical and control samples for MiSeq sequencing with the help of I.T. and I.D.B. in the proof-of-concept study; L.D. prepared the samples for MiSeq sequencing of the DBPC trail; Sa.Wu. did the shotgun metagenome analysis; Sa.Wu. St.Wi., L.D., and E.O. analyzed sequence data; E.O., M.V.B., C.A., and I.S. did part of the microbiological and cell culture lab experiments; I.C., T.H., and F.K. formulated the lactobacilli in the topical cream; S.L. drafted the manuscript; and all authors approved the manuscript.

DECLARATION OF INTERESTS

YUN NV (www.yun.be) was funded as a biotech company focusing on probiotics for the skin after the scientific studies mentioned in this paper. I.C. and T.H. were employed at the University of Antwerp at the time of the study but are currently working at the R&D Department of YUN (www.yun.be). I.C. is now Chief Scientific Officer of YUN NV. T.H. is R&D manager of YUN NV. S.L. and J.L. are members of the scientific advisory board of YUN NV. I.C., T.H., and S.L. are minority stakeholders of YUN. The PhD research of L.D. is currently funded by VLAIO through a Baekeland mandate in collaboration with YUN NV. Based on the data presented here, YUN NV has selected and formulated three *Lactobacillus* strains: *L. pentosus* YUN-V1.0, *L. plantarum* YUN-V2.0, and *L. rhamnosus* YUN-S1.0 in their commercial ACN product. Patents related to this work include dermatological preparations for maintaining and/or restoring healthy skin microbiota (WO2017220525A1) and preservation of micro-organisms (WO2018002248). The remaining authors have no conflicts of interest to declare.

Received: August 28, 2021
Revised: October 29, 2021
Accepted: January 16, 2022
Published: February 15, 2022

REFERENCES

1. Grice, E.A., Kong, H.H., Conlan, S., Deming, C.B., Davis, J., Young, A.C., Bouffard, G.G., Blakesley, R.W., Murray, P.R., Green, E.D., et al. (2009). Topographical and temporal diversity of the human skin microbiome. Science *324*, 1190–1192.
2. Byrd, A.L., Belkaid, Y., and Segre, J.A. (2018). The human skin microbiome. Nat. Rev. Microbiol. *16*, 143–155.
3. Hill, C., Guarner, F., Reid, G., Gibson, G.R., Merenstein, D.J., Pot, B., Morelli, L., Canani, R.B., Flint, H.J., Salminen, S., et al. (2014). Expert consensus document: the International Scientific Association for Probiotics and Prebiotics consensus statement on the scope and appropriate use of the term probiotic. Nat. Rev. Gastroenterol. Hepatol. *11*, 506–514.
4. Nakatsuji, T., Hata, T.R., Tong, Y., Cheng, J.Y., Shafiq, F., Butcher, A.M., Salem, S.S., Brinton, S.L., Rudman Spergel, A.K., Johnson, K., et al. (2021). Development of a human skin commensal microbe for bacteriotherapy of atopic dermatitis and use in a phase 1 randomized clinical trial. Nat. Med. *27*, 700–709.
5. Delanghe, L., Spacova, I., Van Malderen, J., Oerlemans, E., Claes, I., and Lebeer, S. (2021). The role of lactobacilli in inhibiting skin pathogens. Biochem. Soc. Trans. *49*, 617–627.
6. Scholz, C.F.P., and Kilian, M. (2016). The natural history of cutaneous propionibacteria, and reclassification of selected species within the genus propionibacterium to the proposed novel genera acidipropionibacterium gen. Nov., cutibacterium gen. nov. and pseudopropionibacterium gen. nov. Int. J. Syst. Evol. Microbiol. *66*, 4422–4432.
7. Higaki, S. (2003). Lipase inhibitors for the treatment of acne. J. Mol. Catal. B Enzym. *22*, 377–384.
8. Dréno, B. (2017). What is new in the pathophysiology of acne, an overview. J. Eur. Acad. Dermatol. Venereol. *31*, 8–12.
9. Del rosso, J.Q., Webster, G.F., Rosen, T., Thiboutot, D., Leyden, J.J., Gallo, R., Walker, C., Zhanel, G., and Eichenfield, L. (2016). Status report from the scientific panel on antibiotic use in dermatology of the American acne and rosacea society part 1: antibiotic prescribing patterns, sources of antibiotic exposure, antibiotic consumption and emergence of antibiotic resistance, impac. J. Clin. Aesthet. Dermatol. *9*, 18–24.
10. Williams, H.C., Dellavalle, R.P., and Garner, S. (2012). Acne vulgaris. In The Lancet, pp. 361–372.
11. Bernardeau, M., Guguen, M., and Vernoux, J.P. (2006). Beneficial lactobacilli in food and feed: long-term use, biodiversity and proposals for specific and realistic safety assessments. FEMS Microbiol. Rev. *30*, 487–513.
12. Heeney, D.D., Gareau, M.G., and Marco, M.L. (2018). Intestinal Lactobacillus in health and disease, a driver or just along for the ride? Curr. Opin. Biotechnol. *49*, 140–147.
13. Petrova, M.I., Lievens, E., Malik, S., Imholz, N., and Lebeer, S. (2015). Lactobacillus species as biomarkers and agents that can promote various aspects of vaginal health. Front. Physiol. *6*, 1–18.
14. De Boeck, I., van den Broek, M.F.L., Allonsius, C.N., Spacova, I., Wittouck, S., Martens, K., et al. (2020). Lactobacilli Have a Niche in the Human Nose. Cell Reports *31*, 107674.
15. Huttenhower, C.; Human Microbiome Project Consortium (2012). Structure, function and diversity of the healthy human microbiome. Nature *486*, 207–214.
16. Chng, K.R., Tay, A.S.L., Li, C., Ng, A.H.Q., Wang, J., Suri, B.K., Matta, S.A., McGovern, N., Janela, B., Wong, X.F.C.C., et al. (2016). Whole metagenome profiling reveals skin microbiome-dependent susceptibility to atopic dermatitis flare. Nat. Microbiol. *1*, 16106.
17. Oh, J., Byrd, A.L., Deming, C., Conlan, S., Kong, H.H., Segre, J.A., Barnabas, B., Blakesley, R., Bouffard, G., Brooks, S., et al. (2014). Biogeography and individuality shape function in the human skin metagenome. Nature *514*, 59–64.
18. Tett, A., Pasolli, E., Farina, S., Truong, D.T., Asnicar, F., Zolfo, M., Beghini, F., Armanini, F., Jousson, O., De Sanctis, V., et al. (2017). Unexplored diversity and strain-level structure of the skin microbiome associated with psoriasis. npj Biofilms Microbiomes *3*, 14.
19. Duar, R.M., Lin, X.B., Zheng, J., Martino, M.E., Grenier, T., Pérez-Muñoz, M.E., Leulier, F., Gänzle, M., and Walter, J. (2017). Lifestyles in transition: evolution and natural history of the genus Lactobacillus. FEMS Microbiol. Rev. *41*, S27–S48.
20. Pasolli, E., Schiffer, L., Manghi, P., Renson, A., Obenchain, V., Truong, D.T., Beghini, F., Malik, F., Ramos, M., Dowd, J.B., et al. (2017). Accessible, curated metagenomic data through ExperimentHub. Nat. Methods *14*, 1023–1024.
21. Olm, M.R., Brown, C.T., Brooks, B., Firek, B., Baker, R., Burstein, D., Soenjoyo, K., Thomas, B.C., Morowitz, M., and Banfield, J.F. (2017). Identical bacterial populations colonize premature infant gut, skin, & oral microbiomes & exhibit different in situ growth rates. Genome Res *27*, 601–612.
22. Ferretti, P., Pasolli, E., Tett, A., Asnicar, F., Gorfer, V., Fedi, S., Armanini, F., Truong, D.T., Manara, S., Zolfo, M., et al. (2018). Mother-to-Infant microbial transmission from different body sites shapes the developing infant gut microbiome. Cell Host Microbe *24*, 133–145.e5.
23. Zheng, J., Wittouck, S., Salvetti, E., Franz, C.M.A.P., Harris, H.M.B., Mattarelli, P., O'Toole, P.W., Pot, B., Vandamme, P., Walter, J., et al. (2020). A taxonomic note on the genus Lactobacillus: description of 23 novel genera, emended description of the genus Lactobacillus Beijerinck 1901, and union of Lactobacillaceae and Leuconostocaceae. Int. J. Syst. Evol. Microbiol. *70*, 2782–2858.
24. Chu, D.M., Ma, J., Prince, A.L., Antony, K.M., Seferovic, M.D., and Aagaard, K.M. (2017). Maturation of the infant microbiome community structure and function across multiple body sites and in relation to mode of delivery. Nat. Med. *23*, 314–326.
25. Banerjee, S., Schlaeppi, K., and van der Heijden, M.G.A. (2018). Keystone taxa as drivers of microbiome structure and functioning. Nat. Rev. Microbiol. *16*, 567–576.
26. Kankainen, M., Paulin, L., Tynkkynen, S., Von Ossowski, I., Reunanen, J., Partanen, P., Satokari, R., Vesterlund, S., Hendrickx, A.P.A., Lebeer, S., et al. (2009). Comparative genomic analysis of Lactobacillus rhamnosus GG reveals pili containing a human-mucus binding protein. Proc. Natl. Acad. Sci. U S A *106*, 17193–17198.
27. Kleerebezem, M., Boekhorst, J., van Kranenburg, R., Molenaar, D., Kuipers, O.P., Leer, R., Tarchini, R., Peters, S.A., Sandbrink, H.M., Fiers, M.W.E.J., et al. (2003). Complete genome sequence of Lactobacillus plantarum WCFS1. Proc. Natl. Acad. Sci. *100*, 1990–1995.

CellPress
OPEN ACCESS

28. Anukam, K.C., Macklaim, J.M., Gloor, G.B., Reid, G., Boekhorst, J., Renckens, B., van Hijum, S.A.F.T., and Siezen, R.J. (2013). Genome sequence of lactobacillus pentosus KCA1: vaginal isolate from a healthy premenopausal woman. PLoS One *8*, e59239.

29. Segers, M.E., and Lebeer, S. (2014). Towards a better understanding of Lactobacillus rhamnosus GG - host interactions. Microb. Cell Fact. *13*, S7.

30. van den Nieuwboer, M., van Hemert, S., Claassen, E., and de Vos, W.M. (2016). Lactobacillus plantarum WCFS1 and its host interaction: a dozen years after the genome. Microb. Biotechnol. *9*, 452–465.

31. Tapiovaara, L., Lehtoranta, L., Poussa, T., Mäkivuokko, H., Korpela, R., and Pitkäranta, A. (2016). Absence of adverse events in healthy individuals using probiotics - analysis of six randomised studies by one study group. Benef. Microbes *7*, 161–169.

32. van Baarlen, P., Troost, F.J., van Hemert, S., van der Meer, C., de Vos, W.M., de Groot, P.J., Hooiveld, G.J.E.J., Brummer, R.-J.M., and Kleerebezem, M. (2009). Differential NF-kB pathways induction by Lactobacillus plantarum in the duodenum of healthy humans correlating with immune tolerance. Proc. Natl. Acad. Sci. *106*, 2371–2376.

33. van Baarlen, P., Troost, F., van der Meer, C., Hooiveld, G., Boekschoten, M., Brummer, R.J.M., and Kleerebezem, M. (2011). Human mucosal in vivo transcriptome responses to three lactobacilli indicate how probiotics may modulate human cellular pathways. Proc. Natl. Acad. Sci. U S A *108* (*Suppl*), 4562–4569.

34. Skovbjerg, S., Roos, K., Holm, S.E., Grahn Håkansson, E., Nowrouzian, F., Ivarsson, M., Adlerberth, I., and Wold, A.E. (2009). Spray bacteriotherapy decreases middle ear fluid in children with secretory otitis media. Arch. Dis. Child. *94*, 92–98.

35. Reid, G., and Bruce, A.W. (2001). Selection of lactobacillus strains for urogenital probiotic applications. J. Infect. Dis. *183*, 77–80.

36. Mohammedsaeed, W., McBain, A.J., Cruickshank, S.M., O'Neill, C.A., and Neill, A.O. (2014). Lactobacillus rhamnosus GG inhibits the toxic effects of Staphylococcus aureus on epidermal keratinocytes. Appl. Environ. Microbiol. *80*, 5773–5781.

37. Mohammedsaeed, W., Cruickshank, S., McBain, A.J., and O'Neill, C.A. (2015). Lactobacillus rhamnosus GG lysate increases Re-epithelialization of keratinocyte scratch assays by promoting migration. Sci. Rep. *5*, 16147.

38. O'Neill, C.A., Sultana, R., McBain, A.J., and Lysates, B. (2013). Strain-dependent augmentation of tight-junction barrier function in human primary epidermal keratinocytes by lactobacillus and. Appl. Environ. Microbiol. *79*, 4887–4894.

39. Tong, S.Y.C., Davis, J.S., Eichenberger, E., Holland, T.L., and Fowler, V.G. (2015). Staphylococcus aureus infections: epidemiology, pathophysiology, clinical manifestations, and management. Clin. Microbiol. Rev. *28*, 603–661.

40. Sanders, M.E., Benson, A., Lebeer, S., Merenstein, D.J., and Klaenhammer, T.R. (2018). Shared mechanisms among probiotic taxa: implications for general probiotic claims. Curr. Opin. Biotechnol. *49*, 207–216.

41. Tang, S.C., and Yang, J.H. (2018). Dual effects of alpha-hydroxy acids on the skin. Molecules *23*, 863.

42. Zankari, E., Hasman, H., Cosentino, S., Vestergaard, M., Rasmussen, S., Lund, O., Aarestrup, F.M., and Larsen, M.V. (2012). Identification of acquired antimicrobial resistance genes. J. Antimicrob. Chemother. *67*, 2640–2644.

43. Chen, L., Xiong, Z., Sun, L., Yang, J., and Jin, Q. (2012). VFDB 2012 update: toward the genetic diversity and molecular evolution of bacterial virulence factors. Nucleic Acids Res *40*, D641–D645.

44. European Food Safety Authority (2012). Guidance on the assessment of bacterial susceptibility to antimicrobials of human and veterinary importance. EFSA J *10*, 2740.

45. DeFilipp, Z., Bloom, P.P., Torres Soto, M., Mansour, M.K., Sater, M.R.A., Huntley, M.H., Turbett, S., Chung, R.T., Chen, Y.-B., and Hohmann, E.L. (2019). Drug-resistant E. coli bacteremia transmitted by fecal microbiota transplant. N. Engl. J. Med *381*, 2043–2050.

46. Broeckx, G., Vandenheuvel, D., Claes, I.J.J., Lebeer, S., and Kiekens, F. (2016). Drying techniques of probiotic bacteria as an important step towards the development of novel pharmabiotics. Int. J. Pharm. *505*, 303–318.

47. Basketter, D.A., Whittle, E., Griffiths, H.A., and York, M. (1994). The identification and classification of skin irritation hazard by a human patch test. Food Chem. Toxicol. *32*, 773–775.

48. Queille-Roussel, C., Poncet, M., Mesaros, S., Clucas, A., Baker, M., and Soloff, A.M. (2001). Comparison of the cumulative irritation potential of adapalene gel and cream with that of erythromycin/tretinoin solution and gel and erythromycin/isotretinoin gel. Clin. Ther. *23*, 205–212.

49. Feldman, S.R., and Chen, D.M. (2011). How patients experience and manage dryness and irritation from acne treatment. J. Drugs Dermatol *10*, 605–608.

50. Yoon, S.H., Ha, S.M., Kwon, S., Lim, J., Kim, Y., Seo, H., and Chun, J. (2017). Introducing EzBioCloud: a taxonomically united database of 16S rRNA gene sequences and whole-genome assemblies. Int. J. Syst. Evol. Microbiol. *67*, 1613–1617.

51. Kwon, H.H., and Suh, D.H. (2016). Recent progress in the research about Propionibacterium acnes strain diversity and acne: pathogen or bystander? Int. J. Dermatol. *55*, 1196–1204.

52. Lebeer, S., Vanderleyden, J., and De Keersmaecker, S.C.J. (2010). Host interactions of probiotic bacterial surface molecules: comparison with commensals and pathogens. Nat. Rev. Microbiol. *8*, 171–184.

53. Eichenfield, L.F., Sugarman, J.L., Guenin, E., Harris, S., and Bhatt, V. (2019). Novel tretinoin 0.05% lotion for the once-daily treatment of moderate-to-severe acne vulgaris in a preadolescent population. Pediatr. Dermatol. *36*, 193–199.

54. Zeeuwen, P.L.J.M., Boekhorst, J., van den Bogaard, E.H., de Koning, H.D., van de Kerkhof, P.M.C., Saulnier, D.M., van Swam, I.I., van Hijum, S.A.F.T., Kleerebezem, M., Schalkwijk, J., et al. (2012). Microbiome dynamics of human epidermis following skin barrier disruption. Genome Biol *13*, R101.

55. Li, X., Yuan, C., Xing, L., and Humbert, P. (2017). Topographical diversity of common skin microflora and its association with skin environment type: an observational study in Chinese women. Sci. Rep. *7*, 18046.

56. Thierry, A., Deutsch, S.M., Falentin, H., Dalmasso, M., Cousin, F.J., and Jan, G. (2011). New insights into physiology and metabolism of Propionibacterium freudenreichii. Int. J. Food Microbiol. *149*, 19–27.

57. Geoghegan, J.A., Irvine, A.D., and Foster, T.J. (2018). Staphylococcus aureus and atopic dermatitis: a complex and evolving relationship. Trends Microbiol. *26*, 484–497.

58. Ho, A.W., and Kupper, T.S. (2019). T cells and the skin: from protective immunity to inflammatory skin disorders. Nat. Rev. Immunol. *19*, 490–502.

59. van den Broek, M.F.L., De Boeck, I., Claes, I.J.J., Nizet, V., and Lebeer, S. (2018). Multifactorial inhibition of lactobacilli against the respiratory tract pathogen Moraxella catarrhalis. Benef. Microbes *9*, 429–439.

60. Unno, M., Cho, O., and Sugita, T. (2017). Inhibition of Propionibacterium acnes lipase activity by the antifungal agent ketoconazole. Microbiol. Immunol. *61*, 42–44.

61. Allonsius, C.N., Vandenheuvel, D., Oerlemans, E.F.M., Petrova, M.I., Donders, G.G.G., Cos, P., Delputte, P., and Lebeer, S. (2019). Inhibition of Candida albicans morphogenesis by chitinase from selected lactobacilli. Sci. Rep. *9*, 1–12.

62. Kozich, J.J., Westcott, S.L., Baxter, N.T., Highlander, S.K., and Schloss, P.D. (2013). Development of a dual-index sequencing strategy and curation pipeline for analyzing amplicon sequence data on the miseq illumina sequencing platform. Appl. Environ. Microbiol. *79*, 5112–5120.

63. Suzuki, M.T., and Giovannoni, S.J. (1996). Bias caused by template annealing in the amplification of mixtures of 16S rRNA genes by PCR. Appl. Environ. Microbiol. *62*, 625–630.

64. Callahan, B.J., McMurdie, P.J., Rosen, M.J., Han, A.W., Johnson, A.J.A., and Holmes, S.P. (2016). DADA2: high-resolution sample inference from Illumina amplicon data. Nat. Methods *13*, 581–583.

65. Jervis-Bardy, J., Leong, L.E.X., Marri, S., Smith, R.J., Choo, J.M., Smith-Vaughan, H.C., Nosworthy, E., Morris, P.S., O'Leary, S., Rogers, G.B., et al. (2015). Deriving accurate microbiota profiles from human samples with low bacterial content through post-sequencing processing of Illumina MiSeq data. Microbiome *3*, 19.

66. McMurdie, P.J., and Holmes, S. (2013). Phyloseq: an R Package for reproducible interactive analysis and graphics of microbiome census data. PLoS One *8*, e61217.

CellPress
OPEN ACCESS

STAR★METHODS

KEY RESOURCES TABLE

REAGENT or RESOURCE	SOURCE	IDENTIFIER
Bacterial strains		
Lacticaseibacillus rhamnosus GG	Willem de Vos (Kankainen et al., 2009)	ATCC53103
Lactiplantibacillus plantarum WCFS1	Kleerebezem et al. (2003)	WCFS1
Lactiplantibacillus pentosus KCA1	Anukam et al. (2013)	KCA1
Lacticaseibacillus casei	American Type Culture Collection (ATCC)	ATCC334
Lacticaseibacillus casei shirota	Commercial probiotic product	N/A
Lacticaseibacillus casei DN-114001	Commercial probiotic product	DN-114001
Lactobacillus crispatus LMG12005	BCCM/LMG	LMG12005
Lactobacillus helveticus 1807	Commercial probiotic product	1807
Lacticaseibacillus paracasei LMG12586	BCCM/LMG	LMG12586
Lactiplantibacillus pentosus ATCC8041	BCCM/LMG	ATCC8041
Lactiplantibacillusplantarum ATCC8014	BCCM/LMG	ATCC8014
Lactiplantibacillusplantarum 5057	Yoon et. Al (2017)	5057
Limosilactobacillus reuteri RC14	ATCC	RC14
Lacticaseibacillus rhamnosus GR-1	Segers et al. (2014)	GR-1
Latilactobacillus sakei ATCC15521	ATCC	ATCC15521
Staphylococcus epidermidis ATCC12228	ATCC	ATCC12228
Cutibacterium acnes (previously known as *Propionibacerium acnes*)	ATCC	ATCC6919
Staphylococcus aureus	ATCC	ATCC29213
Biological samples		
adult human skin swabs	University of Antwerp Universital Hospital Antwerp Allergisa, Brazil	N/A
Chemicals, peptides, and recombinant proteins		
de Man, Rogosa and Sharpe (MRS)	Difco	BD288210
Mueller Hinton	LabM limited	LAB039-A
Reinforced clostridial broth	LabM Limited	LAB023
Tween 80 (polysorbate)	Carl Roth Belgium	9139.1
Keratinocyt Growth medium 2	Promocell	C-20111
2,3-Bis(2-methoxy-4-nitro-5-sulfophenyl)-2H-tetrazolium-5-carboxanilide (XTT)	Sigma Aldrich	X12223
FloqSwab	Copan	502CS01
BacSwab	DME	1028
Trizma base	Sigma Aldrich	77-86-1
Ethylenediaminetetraaceticacid (EDTA)	Sigma Aldrich	60-00-4
Tween-20	Biorad	170-6531
PBS	Gibco	14040-091
SeaKem^R LE Agarose	Lonza	50004
1.0N NaOH	Illumina	NA
PhiX sequencing control v3	Illumina	FC-110-3001
Triton X-100	Sigma Aldrich	9036-19-5
Clindamycin	Sigma Aldrich	21462-39-5
PBS	Gibco	14040-091
4-methyl umbelliferyl oleate	Sigma Aldrich	75164-25MG
Tris-HCl	Roche	10812846001

(*Continued on next page*)

Continued

REAGENT or RESOURCE	SOURCE	IDENTIFIER
NaCl	Carl Roth Belgium	3957.1
CaCl2	Carl Roth Belgium	A119.1
Sodium citrate	Carl Roth Belgium	2611.3
Critical commercial assays		
QIAamp Powerfecal DNA kit	Qiagen	12830-50
Agencourt AMPure XP	Beckman Coulter	A63881
NucleoSpin 96 Tissue kit	Machery-Nagel	MN 740609.50
D-Lactic acid/L-lactic acid kit	R-biopharm	11112821035
Deposited data		
DNA-Seq data	This paper	PRJEB27311<br>PRJEB44792
R code	This paper	https://github.com/LebeerLab/skin_acne_study
DNA-Seq data	The Human Microbiome Project Consortium (2012)	https://www.hmpdacc.org/
Experimental models: Cell lines		
Normal Human Epidermal Keratinocytes (NHEK)	Promocell	C-12001
Oligonucleotides		
Barcoded 515F primer (V4 primer) AAT GAT ACG GCG ACC ACC GAG ATC TAC **ACA TCG TAC G**TA TGG TAA TTG TGT GCC AGC MGC CGC GGT AA	Kozich et al. (2013)	N/A
Bacterial *16S rRNA* gene V4 region (806R) GGA CTA CHV GGG TWT CTA AT	Kozich et al. (2013)	N/A
Barcoded 27F primer (V1V2 primer) AAT GAT ACG GCG ACC ACC GAG ATC TAC AC**A AGC AGC A**TA TGG TAA TTC GAG AGT TTG ATC MTG GCT CAG	Suzuki et al. (1996)	N/A
Barcoded 388R primer (V1V2 primer) CAA GCA GAA GAC GGC ATA CGA GAT **ACC TAG TA**A GTC AGT CAG CCG CTG CCT CCC GTA GGA GT	Suzuki et al. (1996)	N/A
Software and algorithms		
GraphPad Prism	GraphPad Software	https://www.graphpad.com/
DADA2, version 1.6.0	Callahan et al. (2016)	https://benjjneb.github.io/dada2/index.html
R version 3.6.3 (R Core Team, 2020)	R Core Team (2020)	https://www.r-project.org/
Tidyamplicons	N/A	github.com/Swittouck/tidyamplicons
MicrobeDS R package	N/A	https://github.com/twbattaglia/MicrobeDS
curatedMetagenomicData R package	Pasolli, et al. (2017)	https://github.com/waldronlab/curatedMetagenomicData
Phyloseq	McMurdie & Holmes (2013)	https://github.com/joey711/phyloseq
EZBioCloud 16S database	Yoon et al. (2017)	https://www.ezbiocloud.net
Other		
MiSeq Desktop sequencer	Illumina	(M00984, Illumina)
Synergy HTX multi-mode reader	Biotek	N/A
Take3	Biotek	N/A
StepOne Plus Real-Time PCR System (v.2.0)	Applied Biosystems	N/A
EVE^{TM} Automatic cell counter	NanoEntek	EVE-MC
Qubit 3.0 Fluorometer	Life Technologies	Q33216
Corneometer MPA 5 CPU	Courage and Khazala	S/N 10359198; probe S/N 11284693

RESOURCE AVAILABILITY

Lead contact

Further information and requests for resources and reagents should be directed to and will be fulfilled by the Lead Contact, Sarah Lebeer (sarah.lebeer@uantwerpen.com).

Materials availability

All unique/stable reagents generated in this study are available from the Lead Contact with a completed Materials Transfer Agreement.

Data and code availability

- Sequencing data of the proof-of-concept clinical trial is available at the European Nucleotide Archive with the accession number PRJEB27311 (https://www.ebi.ac.uk/ena/browser/view/PRJEB27311?show=reads).
- Sequencing data of the placebo-controlled clinical trial is are available with the accession number PRJEB44792 (https://www.ebi.ac.uk/ena/browser/view/PRJEB44792?show=reads).
- The R code generated during this study can be found on GitHub at https://github.com/LebeerLab/skin_acne_study.
- Any additional information required to reanalyze the data reported in this paper is available from the Lead Contact upon request.

EXPERIMENTAL MODEL AND SUBJECT DETAILS

Human subjects

A proof-of-concept clinical trial was performed on patients with mild-to-moderate acne vulgaris included after careful assessment of the responsible dermatologist by counting of comedones and inflammatory lesions (Table S3). Patients were men between 12-25 years. Exclusion criteria were use of oral antibiotics within 4 weeks prior to start of the study and use of systemic retinoids within 6 months prior to start of study. A double-blind placebo-controlled clinical trial was performed on 79 patients wild mild -to-moderate acne vulgaris, aged from 12 to 33 years old (mean age: 18 years old), presenting at least 9 inflammatory lesions and oily skin on the face. Important exclusion criteria were the use of antibiotics within the last 4 months and acne treatments less than 90 months before the study (list of inclusion and exclusion criteria in Methods S1 and S2 and on ClinicalTrials.gov). All subjects provided written informed consent before the study began.

The protocol of this study was in accordance with the Declaration of Helsinki. The POC trial with the first patients was approved by the ethics committee of the University Hospital of Antwerp (Belgium) before initiation of the study. The study was given the approval number B300201628507 (Belgian registration) and registered online at clinicaltrials.gov with unique identifier NCT03469076. The second trial was approved by the Local Ethics Committee (LEC) of Investigation – Instituto de Pesquisas, registered by the National Research Ethics Commission (CONEP) of Brazil. The study was registered online at clinicaltrials.gov with unique identifier NCT04216160 (Methods S2).

Sample collection

Skin samples were collected by brushing the cheek (control group) or the affected area on the face (patients) with a FloqSwab (Copan) (first proof-of-concept study) or a sterile BacSwab (DME) soaked in saline solution (Tris, 50 mM pH 7.6; EDTA 1 mM pH 8.0; Tween 20 0.5%) over an area of ± 10 cm^2 or around the lesions for 15 seconds. Swabs were then transferred to a falcon containing 800 µl Bead solution of QIAamp PowerFecal DNA kit (Qiagen). Samples were stored at 4°C until further processing (maximally 14 days). Before DNA extraction, samples were vortexed for 1 minute, after which the Bead solution was transferred to the bead tube. Subsequent steps of the DNA extraction were executed according to manufacturer's instructions.

Proof-of-concept clinical trial

Patients were asked to apply the topical probiotic cream (containing 10^8 CFU of each *Lactobacillus* strain per application of 1 g of the topical cream) twice daily for 56 days (8 weeks). The patients were seen by a dermatologist at start (before the therapy) (visit 1), week 4 (visit 2), week 8 (visit 3) and week 10 (visit 4). A skin swab was taken at each visit, total DNA was extracted and amplified for 16S amplicon sequencing as described above. Moreover, a clinical scoring was performed, and a photograph taken at each visit.

Double-blind placebo-controlled clinical trial

The study subjects remained in a room with controlled temperature and air relative humidity for at least 30 minutes before the initial measurements and in the interval between them. The initial clinical assessment was performed by a dermatologist to confirm the inclusion and exclusion criteria and the assess the initial state of the skin (Dermatological Clinical Assessment – IGA and DAT -T0). An acne lesions counting was performed by a trained technician. The subjects used the product for 8 weeks ± 2 days and then they suspended its use for 4 weeks ± 2 days. They were assessed before product use (T0), after 2, 4 and 8 weeks ± 2 days of product use (T2w, T4w and T8w, respectively) and after 04 weeks ± 2 days without product use (T12w). On all visits, an acne lesions counting was performed by a trained technician. The study subjects should record all the applications performed and add possible

comments about the product (in a daily-log). They were also be instructed to perform the last product application on the previous day of the assessments at the institute.

METHOD DETAILS

Bacterial growth

Strains of lactobacilli were grown at 37°C in de Man, Rogosa and Sharpe (MRS) medium (BD Difco, Erembodegem, Belgium). *Cutibacterium acnes* ATCC6919 was inoculated in reinforced clostridial broth (LabM Limited, Heywood, UK), supplemented with 0.2% Tween20 and cultured microaerobically (5% CO2) at 37°C. *Staphylococcus aureus* ATCC29213 was grown in Mueller-Hinton broth at 37°C. Solid media contained 1.5% (w/v) agar. Time-course experiments were also performed analysing the antimicrobial activity of spent culture supernatant (SCS) of the selected *Lactobacillus* strains against C. *acnes* and *S. aureus* ATCC29213 (cfr.[59]). Additionally, the impact of this SCS (10%) on the lipase activity of C. *acnes* was determined as previously described.[60] Concentrations of D- and L-lactic acid were measured through a commercially available kit (R-Biopharm, Darmstadt, Germany) as previously described .[61]

Human skin cell culture

Normal human epidermal keratinocytes (NHEK) cells from juvenile foreskin from pooled donors were purchased from Promocell (Heidelberg, Germany) and cultured according to manufacturer's recommendations in Keratinocyt Growth medium 2 (Promocell, Heidelberg, Germany). Cytotoxicity of probiotic strains was assessed using the 2,3-Bis(2-methoxy-4-nitro-5-sulfophenyl)-2H-tetrazolium-5-carboxanilide (XTT, Sigma-Aldrich) cell viability assay. NHEK cells were seeded at a density of 5000 cells/well in 96-well plates and cultured until confluent. Overnight cultures of probiotic strains or *S. aureus* were added to the wells with or without NHEK cells at 10^6 CFU/well and incubated for 2 h at 5% CO2 and 37°C. Triton X-100 (0.5%) was used as a positive control.

Illumina MiSeq *16S rDNA* gene amplicon sequencing

The primers used for Illumina MiSeq sequencing were based on the previously described 27F-338R or 515F-806R primers[62,63] and altered for dual-index paired-end sequencing, as described earlier[62] (Key resources table). Separate runs were carried out for V1V2 and V4 *rRNA* gene variable regions. Quality control and processing of reads was performed using the R package DADA2, version 1.6.0.[64] Denoised reads (amplicon sequence variants or ASVs) were merged and read pairs with one or more conflicting bases between the forward and reverse read were removed. Chimeric sequences were removed using the function "removeBimeraDenovo". Finally, ASVs were classified from the kingdom to the genus level using the EzBioCloud 16S database.[50] A species annotation was added to each ASV by listing the species of all 16S sequences in the database that showed an exact match to the ASV sequence. Contaminants were identified using the approach of Jervis-Bardy et al.[65] ASVs with a strong negative correlation between relative abundances and total sample read counts were considered contamination. For each ASV, this correlation was calculated and tested for significance. ASVs with a p-value less than 0.0001 were removed. Samples were filtered by removing those with less than 1000 reads left after all read and ASV filtering steps. Results for negative controls (for both DNA extraction kits and PCR) can be found in Figure S7.

Biostatistical and bioinformatics analysis

Processing of the ASV table, ASV annotations (e.g. classification) and sample annotations (metadata) were performed using the in-house R package "tidyamplicons", publicly available at github.com/SWittouck/tidyamplicons. For the analyses at the genus level, ASV read counts were aggregated at the genus level or, if unavailable, at the most specific level at which taxonomic annotation was available.

Analysis of public datasets

Processed OTU-table and sample metadata from the Human Microbiome Project (HMPv35)65 and the shotgun metagenomic datasets were retrieved using the MicrobeDS R package and curatedMetagenomicData R package[20] respectively. All data was loaded, processed and visualized in the R-environment using Phyloseq.[66] All scripts are available at https://github.com/LebeerLab/skin_acne_study.

Formulation of lactobacilli in microcapsules and O/W cream

A single colony of the three selected probiotic strains was grown until stationary phase and lyophilized (± 10^{11} CFU/gram) and subsequently encapsulated via a core-shell encapsulation approach. Briefly, the strains were mixed in equal amounts and homogeneously suspended to obtain a stable oil-based feed core suspension. The shell feed solution contained a hydrocolloid alginate polymer as gelling agent. Both liquid feeds were pumped to a concentric nozzle, to obtain a concentric fluid flow. The laminar liquid flow was broken up by a vibrational unit to obtain spherical -droplets that were solidified upon falling in a calcium-based solidification solution, forming the capsules. The collected capsules (10^9 - 10^{10} CFU/gram) were washed and suspended in an oil-in-water cream. The ingredients of this cream, mainly the emulsifiers and preservatives, were selected to be compatible with the micro-capsules and bacteria, both during storage and upon release of the probiotics. Hereto, the impact of the topical cream without the capsules on the growth of four skin reference bacteria (*S. aureus, S. epidermidis, L. crispatus* and *C. acnes*) was evaluated at a concentration of 1, 10 and 100 mg/ml, by a time-course analysis of OD600 measurements as described above. Mechanical force (rubbing on the skin) was

confirmed to break the capsules, releasing the inner core material containing the suspended probiotics. Skin irritation tests with the cream containing the encapsulated lactobacilli were performed as described previously.[47]

Determination of skin hydration (Corneometry)

The capacity to improve skin hydration of the cream with lactobacilli was tested after 14 and 28 days of use, twice daily, by 20 healthy volunteers (all female, average age 48,6, inner sides of forearms). Skin hydration was measured with Corneometer MPA 5 CPU (Courage and Khazaka, Cologne, Germany; S/N 10359198; probe S/N 11284693) by registering the electrical capacitance of the skin surface. Five measurements were performed on each test area and the mean was used to define the hydration state of the stratum corneum.

QUANTIFICATION AND STATISTICAL ANALYSIS

Statistical details can be found in the figure legends. Statistical analyses were performed in R Studio, using a Mann-Whitney test, Two-way ANOVA or pairwise Wilcoxon test with Holm correction for multiple testing.

ADDITIONAL RESOURCES

The POC trial with the first patients was approved by the ethics committee of the University Hospital of Antwerp (Belgium) before initiation of the study. The study was given the approval number B300201628507 (Belgian registration) and registered online at clinicaltrials.gov with unique identifier: https://clinicaltrials.gov/ct2/show/NCT03469076?term=NCT03469076&draw=2&rank=1

The second trial was approved by the Local Ethics Committee (LEC) of Investiga – Instituto de Pesquisas, registered by the National Research Ethics Commission (CONEP) of Brazil. The study was registered online at clinicaltrials.gov with unique identifier: https://clinicaltrials.gov/ct2/show/NCT04216160?term=NCT04216160.&draw=2&rank=1

Cell Reports Medicine, Volume *3*

Supplemental information

Selective targeting of skin pathobionts and inflammation with topically applied lactobacilli

Sarah Lebeer, Eline F.M. Oerlemans, Ingmar Claes, Tim Henkens, Lize Delanghe, Sander Wuyts, Irina Spacova, Marianne F.L. van den Broek, Ines Tuyaerts, Stijn Wittouck, Ilke De Boeck, Camille N. Allonsius, Filip Kiekens, and Julien Lambert

Supplementary information

Supplementary Figures

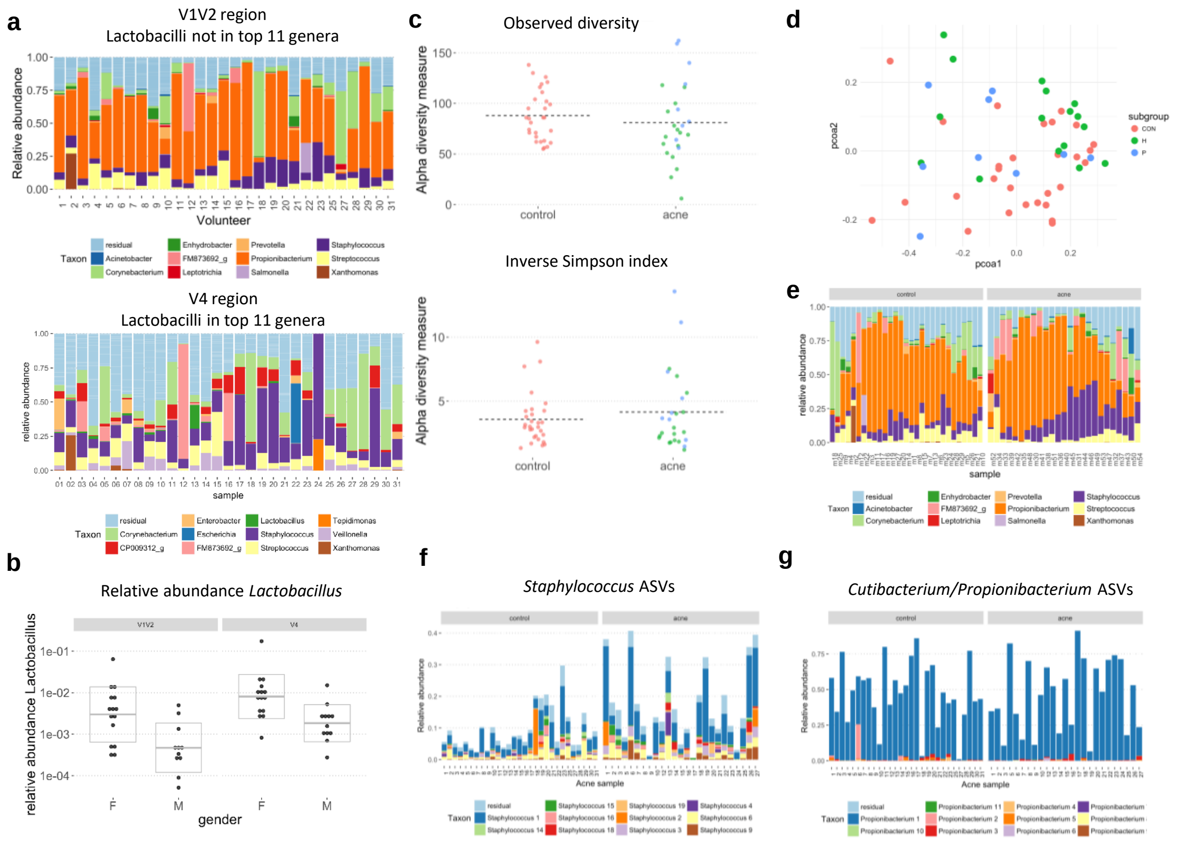

Figure S1: Microbiome analysis of facial skin samples of control subjects (n= 30) (without acne symptoms) and comparison with subjects with mild-to moderate acne symptoms (n = 27) at baseline, without treatment.

(a) Barplots showing taxonomy of the control group of volunteers (n= 30) without acne symptoms, as derived from V1V2 region (top) and V4 region (bottom) of the rRNA gene sequencing (b) Gender-specific relative abundance data for *taxa* of lactobacilli in the control group based on the different variable regions sequenced (V1V2 or V4) (c) Alpha-diversity of samples as indicated by observed diversity (richness, left) and Inverse Simpson index (richness and evenness, right). (d) PCOA plot of baseline samples, based on Bray-Curtis distances between samples. Samples of the control group are shown in red, while acne samples of subjects that were included for the study treatment (n= 10) are indicated in blue and acne samples of subjects that were not included in the study treatment are shown in green (n= 17). (e) Barplot showing taxonomy (derived from V1V2 rRNA gene sequencing data) at genus level of the control and acne group. Samples are divided by group and clustered hierarchically to minimize Bray-Curtis distance. (f) Barplot showing relative abundances of 11 most abundant *Staphylococcus* ASVs in the control and acne group. (g) Barplot showing relative abundances of 11 most abundant *Propionibacterium/Cutibacterium* ASVs in the control and acne group. Related to Figure 1.

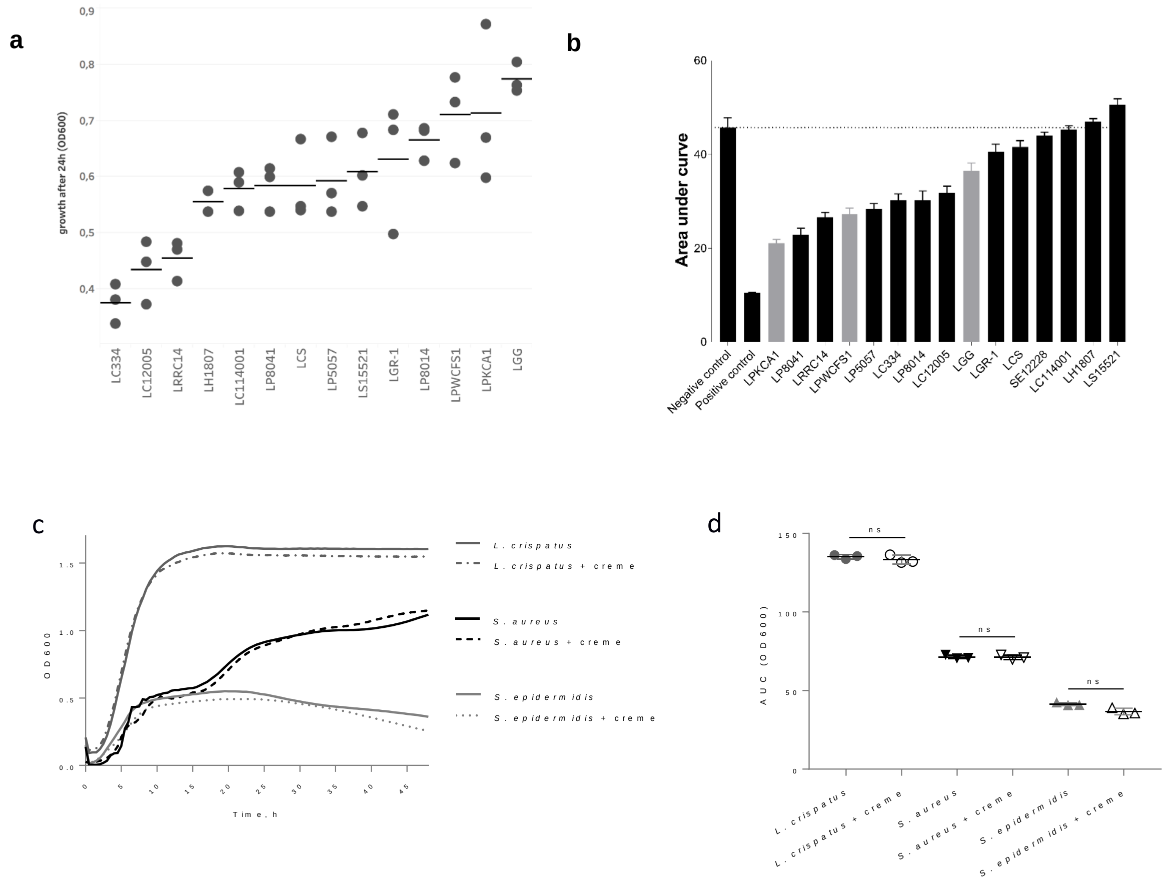

Figure S2: *In vitro* selection of the lactobacilli strains

(a) Optical densities of the cultures of lactobacilli tested after inoculation of MRS broth with 1% preculture and 24h incubation at 37°C. (b) Bacterial growth of *C. acnes* in the presence of Lactobacillus SCS. The antimicrobial activity of the SCS of the selected Lactobacillus strains against C. acnes is depicted as the area under the growth curve (OD600) and compared to the positive control (10 mg/mL Clindamycin, a common antibiotic used in acne). MRS at pH4, which is comparable to the pH of the SCS of lactobacilli, was used as a negative control. (B) Lactic acid bacteria used: *LPKCA1: L. pentosus KCA1, LP8041: L. plantarum ATCC 8041, LRRC14: L. reuteri RC14, LPWCFS1: L. plantarum WCFS1, LP5057: L. plantarum 5057, LC334: L. casei ATCC334, LP8014: L. pentosus ATCC 8014, LC12005: L. crispatus LMG 12005, LGG: L. rhamnosus GG, LGR-1: L. rhamnosus GR-1, LCS: L. casei Shirota, SE12228: S. epidermidis ATCC 12228, LC114001: L. casei DN-114001, LH1807: L. helveticus 1807, LS15521: L. sakei ATCC 15521.* (c) Impact of the ingredients of the topical cream without microcapsules on the growth capacity of the skin bacteria *Lactobacillus crispatus, C. acnes, Staphylococcus aureus* and *Staphylococcus epidermidis*. Growth curves of *L. crispatus*, *S. aureus* and *S. epidermidis* without (full lines) or with addition of topical cream ingredients at 1 mg/ml (dotted lines). Similar data were obtained for 100 mg/ml, but then we also observed more interference of the oily cream ingredients and the OD measurements. Data depicted as mean OD600 values plotted over the course of 48 hours; (d) Area under the curve (AUC) of *L. crispatus, S. aureus* and *S. epidermidis* growth curves with or without addition of topical cream. Individual AUC values depicted per tested well repetition with mean and SD per group, n = 3; ns: non-significant p-value (> 0.05) as calculated by the Mann-Whitney U-test**.** Related to Figure 2.

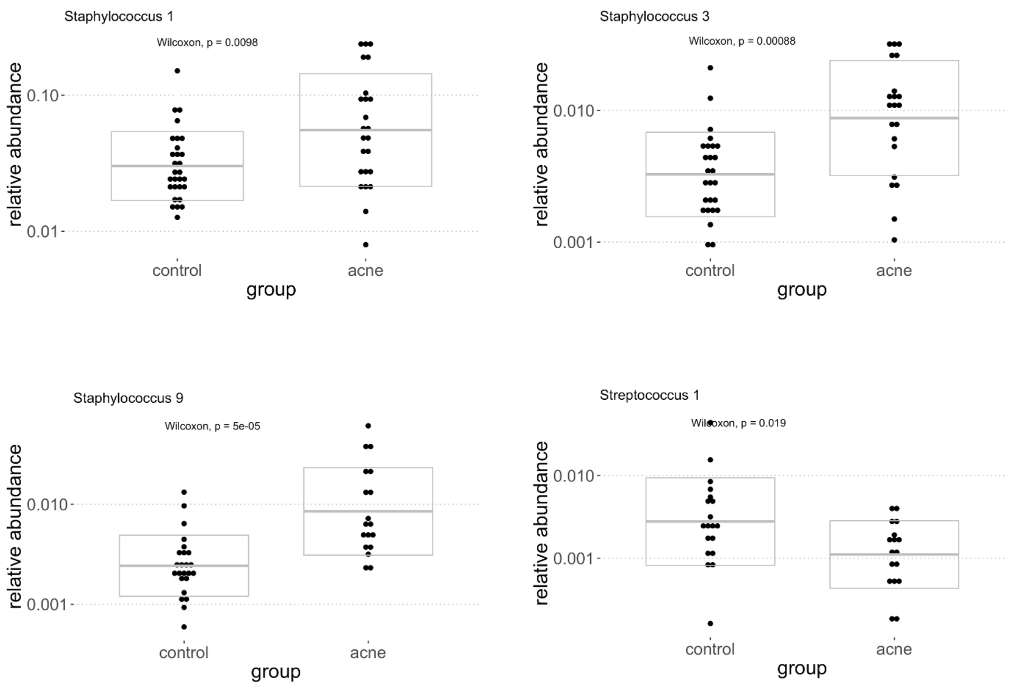

Figure S3: **Relative abundances of specific ASVs in the control group (n = 30) versus acne patients (n = 27)**

Staphylococcus 1, Staphylococus 3,, Staphylococcus 9 (all annotated as *Staphylococcus epidermidis)* and *Streptococcus 1 (*annotated as *Streptococcus salivarius* according to EZ taxon) that showed significantly higher (A-C) or lower (D) relative abundances in the acne group as compared to the control group at baseline. To test for significant differences, a Kruskal-Wallis test was performed and p-values are indicated in the graphs. It is important to not here that although we suggest possible species-level classification here, this is actually not possible based on ASV analysis alone. These annotations should therefore be considered while keeping in mind the uncertainty for such classification. Related to Figure 4.

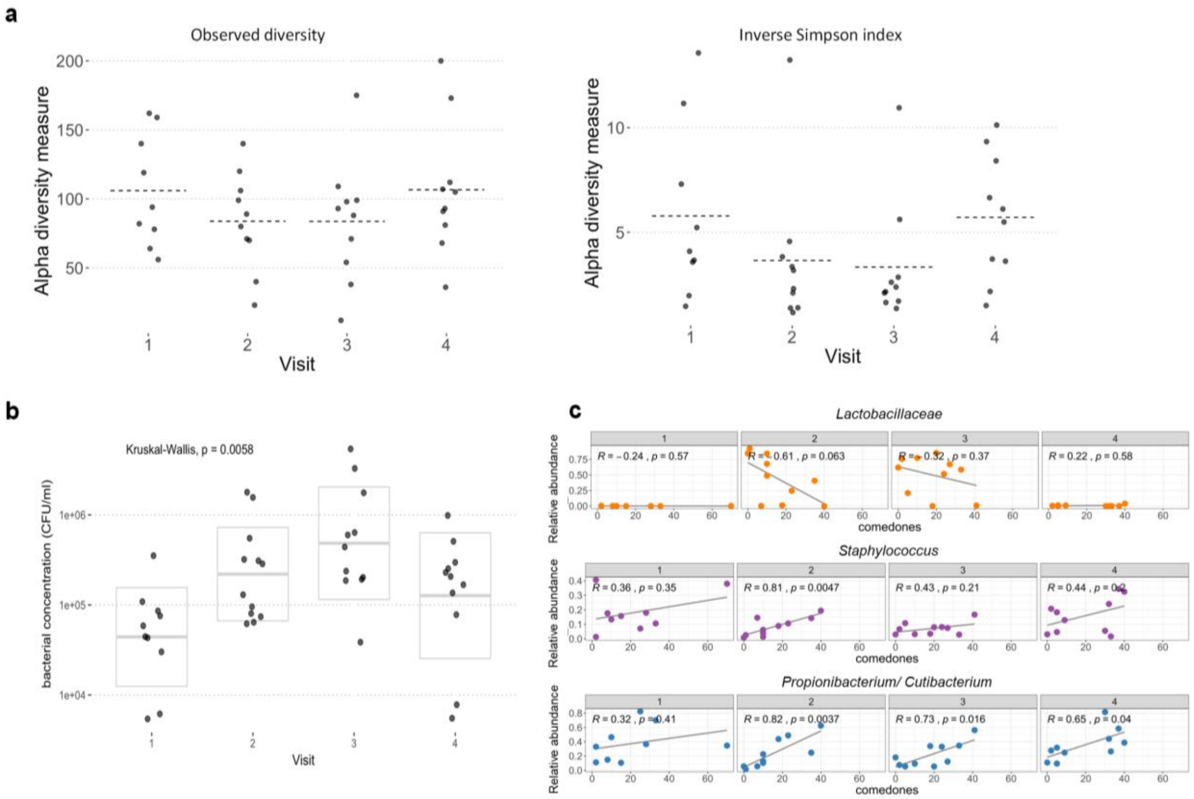

Figure S4: Impact of the use of the probiotic cream on the skin microbiome.

(a) Alpha-diversity as indicated by observed diversity (richness) and Inverse Simpson index (richness and evenness) of the samples throughout the study period, grouped by visit. (b) Estimated total bacterial concentration in the samples. Bacterial concentrations were estimated by quantitative PCR and a standard curve. (c) Correlation between bacterial taxa *Lactobacillaceae* (top), *Staphylococcus* (middle) and *Cutibacterium/ Propionibacterium* (bottom) and comedone count for each visit (x-axis)., Pearson's correlation coefficient is indicated. Related to Figure 4.

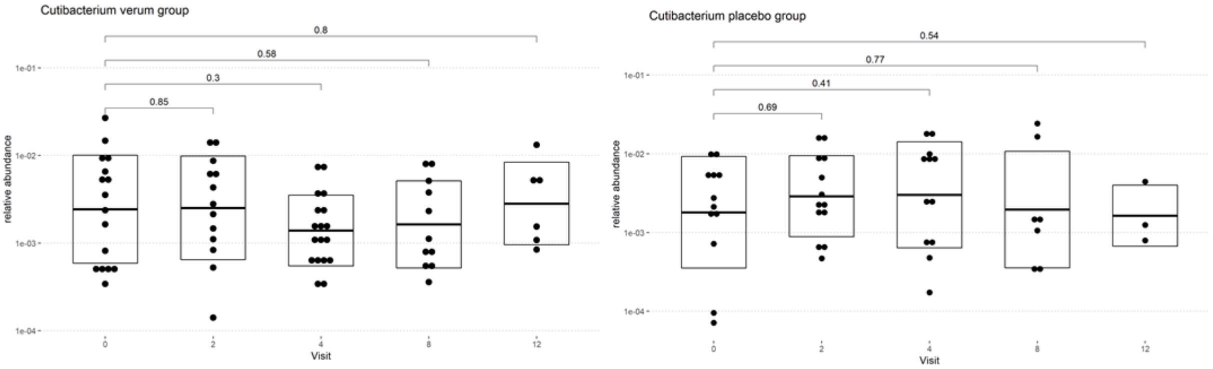

Figure S5: Impact of the lactobacilli cream on the relative abundance of *Cutibacterium sp.* – DBPC study

Relative abundances of *Cutibacterium sp.* in verum and placebo group during the study period, as found through 16S sequencing of the V4 hypervariable region. Related to Figure 5.

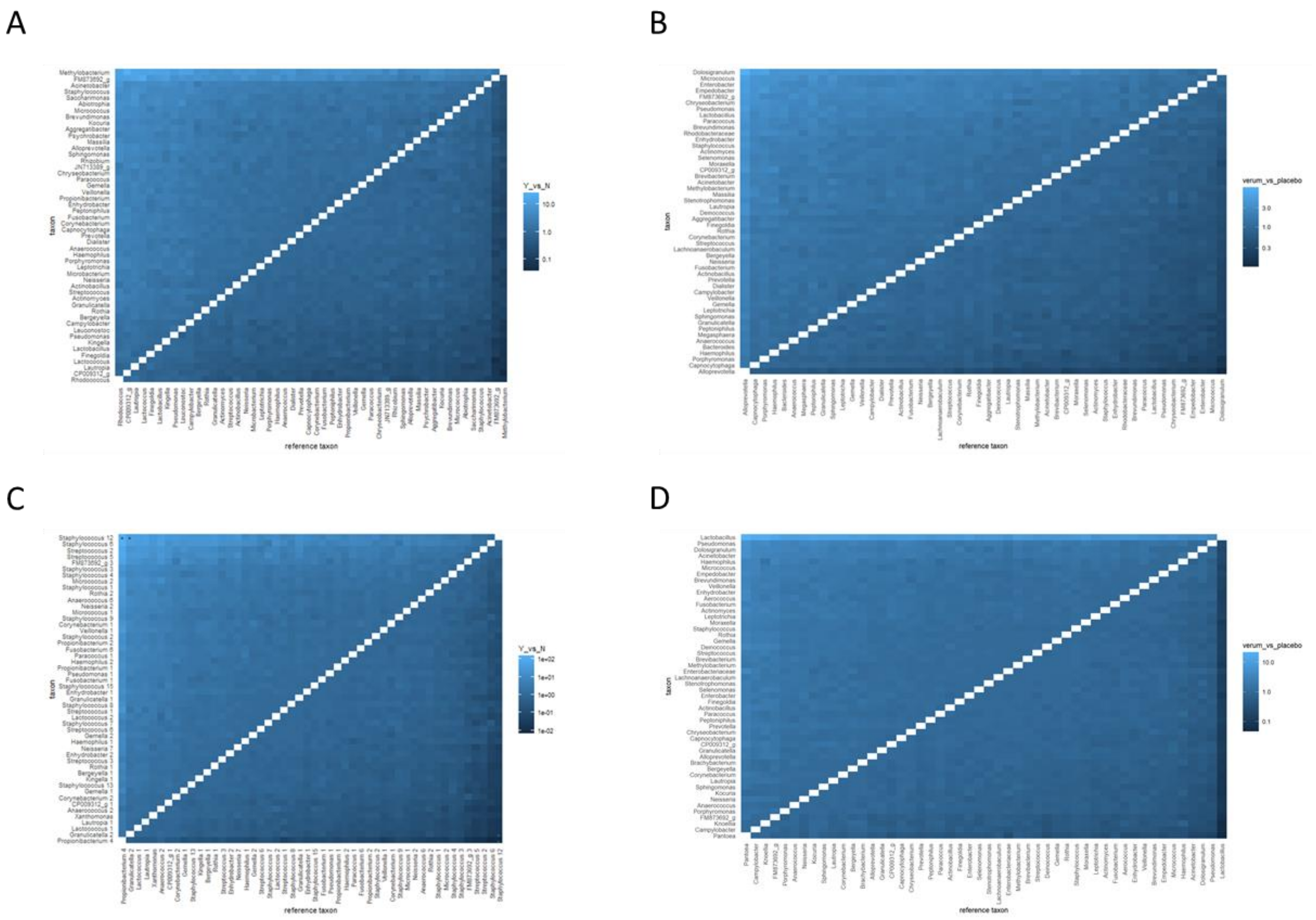

Figure S6: Codifab plots showing differential relative abundance at genus level between two groups of participants for both POC and DBPC study.

(a) Baseline of POC study: differential relative abundance for the most abundant genera between the healthy control group and acne patients at the time of inclusion. (b) Baseline of DBPC study: differential relative abundance for the most abundant genera between the verum group and placebo group at the time of inclusion. (c) POC study at visit 1: differential relative abundance for the most abundant genera between the healthy control group and acne patients after 4 weeks of treatment with a probiotic cream containing live lactobacilli. (d) DBPC study at visit 1: differential relative abundance for the most abundant genera between the verum and placebo group after 2 weeks of treatment with respectively the probiotic cream and placebo cream. + or – indicates significance in differential relative abundance for that specific genus. Related to Figure 6.

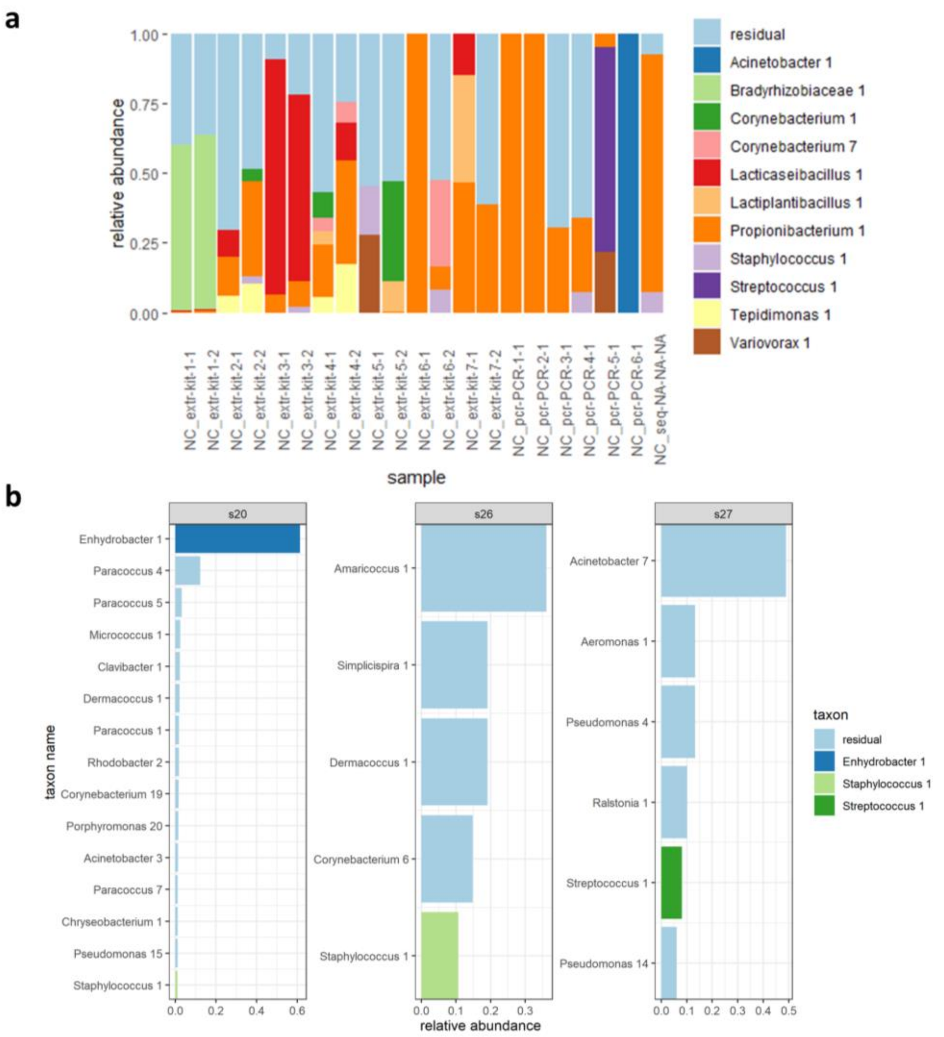

Figure S7: Taxonomic classification of the negative controls for the DNA extraction kits and PCR for the pilot study (a) and the placebo-controlled study (b)

(a) Negative controls for the pilot study included both kit controls and PCR negative controls. Read counts were generally relatively low in comparison to the samples (median controls: 431 reads; median samples: 44773.5 reads). (b) Negative controls for the placebo-study included only controls for PCR. 5 of 8 controls did not yield any reads after quality control. Related to STAR methods.

Supplementary Tables

Table S1: Bacterial strains used in this study. Related to Figure 2.

Species	Strain	Relevant genotype or description	Reference and/or source	Final selection for formulation in cream & POC study *
Lactocaseibacillus rhamnosus	GG	Single colony isolate of wild-type strain, isolated from human faeces	[19]	yes
Lactoplantibacillus plantarum	WCFS1	Single colony isolate of *L. plantarum* WCFS1	[20]	yes
Lactiplantibacillus pentosus	KCA1	Single colony isolate from KCA1 (vaginal origin)	[21]	yes
Lacticaseibacillus casei	ATCC334	Single colony isolate obtained from a stock culture of ATCC334	ATCC	No, less active than WCFS1 & KCA1 against C. acnes & *S. aureus*

Lacticaseibacillus casei* ***(LCS)***	Shirota	Single colony isolate obtained from a commercially available fermented drink containing *L. casei* Shirota (Yakult®), confirmed by sequencing	Commercial probiotic product	No, less active than WCFS1 & KCA1 against *C. acnes* and & *S. aureus*
Lacticaseibacillus casei*	DN-114001	Single colony isolate obtained in our lab from a commercially available fermented drink (Actimel®) containing *L. casei* DN-114001, confirmed by sequencing	Commercial probiotic product	No, less active than WCFS1 & KCA1 against *C. acnes* and & *S. aureus*
Lactobacillus crispatus	LMG12005		BCCM/LMG	No, less robust & less growth capacity than other lactobacilli tested
Lactobacillus helveticus	1807	single colony isolate	Commercial probiotic product	No, less active than WCFS1 & KCA1 against *C. acnes* and & *S. aureus*
Lacticaseibacillus paracasei	LMG12586	Single colony isolate obtained from a stock culture of LMG12586	BCCM/LMG	No, less active than WCFS1 & KCA1 against *C. acnes* and & *S. aureus*
Lactiplantibacillus pentosus	ATCC8041		BCCM/LMG	No, less active than WCFS1 & KCA1 against *C. acnes* and & *S. aureus*
Lactiplantibacillus splantarum	ATCC8014	Single colony isolate from *L. plantarum* ATCC8014 or LMG1284	BCCM/LMG	No, less active than WCFS1 & KCA1 against *C. acnes* and & *S. aureus*
Lactiplantibacillu splantarum	5057	Single colony isolate of *L. plantarum* 5057	[49]	No, presence of antibiotic resistance genes (Tetracycline)
Limosilactobacillus reuteri	RC14	Single colony isolate from a commercially available probiotic supplement containing *L. reuteri* RC14, confirmed by sequencing	ATCC	No, because slight induction of inflammation in primary skin cells
Lacticaseibacillus rhamnosus	GR-1	Single colony isolate obtained from a commercially available probiotic supplement containing *L. rhamnosus* GR-1 (urethra origin)	[28]	No, less active than WCFS1 & KCA1 against *C. acnes* and & *S. aureus*
Latilactobacillus sakei	ATCC15521		BCCM/LMG	Not active against *C. acnes* and & *S. aureus*
Staphylococcus epidermidis	ATCC12228		BCCM/LMG	No GRAS/QPS status

* Main reason for non-selection is indicated, but also other aspects such as availability and freedom-to-operate, growth capacity, and level of scientific documentation in the literature were considered.

Table S2: Clinical data of the skin patch test results for the O/W *Lactobacillus* cream. Related to Figure 3.

Vol ID	Volunteer code	Gender	Age	Erythema	Dryness	Edema	Tot readings 48hrs
1	1176	F	55	0	0	0	0
2	1182	F	25	0	0	0	0
3	1191	F	24	0	0	0	0
4	1202	F	54	0	0	0	0
5	1211	M	59	0	0	0	0
6	1258	M	29	0	0	0	0
7	1263	F	64	0	0	0	0

8	1335	M	47	0	0	0	0
9	1364	F	50	0	0	0	0
10	1458	F	22	0	0	0	0
11	1509	F	30	0	0	0	0
12	1549	F	23	0	0	0	0
13	1623	F	24	0	0	0	0
14	1700	F	44	0	0	0	0
15	1701	M	41	0	0	0	0
16	1702	M	24	0	0	0	0
17	1703	M	25	0	0	0	0
18	1704	M	24	0	0	0	0
19	1705	F	31	0	0	0	0
20	1706	F	20	0	0	0	0

Table S3: Pilot trial's patients characteristics and clinical evaluation after 4 and 8 weeks of treatment compared to baseline. Related to Figure 5.

Patient ID	Adverse effects	Inflammatory lesion count (% decrease)		Comedonal count (% decrease)		Remarks
		4 weeks	8 weeks	4 weeks	8 weeks	
1	no	12.0	40.0	50.0	71.4	Patient saw first improvements after 1st week. Global improvement at V2. Further improvement at V3 but with fluctuation. Symptoms reappearing 3 days after stop of treatment
2	no	26.9	65.4	0.0	-2.5	Slight reduction in comedones and papules at V2 Improvement continued at V3 but with fluctuation. At visit 4 still improvement compared to baseline.
3	no	80.0	100.0	35.7	35.7	Mostly comedonal acne. Patient was very positive with improvement observed after 3 days. Reduction in comedones at V2, V3 and V4.
4	no	53.8	15.4	30.3	27.3	At V2 still multiple comedones on forehead but improvement on cheeks (no more pustules) and chin (reduction in pustules). At V3 continued improvement. Only some inflammatory lesions on forehead but only comedones on chin, nose and cheeks. At V4, after stop of treatment, quick relapse of symptoms.
5	no	66.7	48.1	60.0	-32.0	Patient noticed an increase in comedones in first 2 weeks but global improvement at V2. Patient follow-up less consistent between V2 and V3, which resulted in reappearing of symptoms. At V4, after stop of treatment, relapse of symptoms.
6	no	80.0	95.0	50.0	0.0	Only slight improvement at V2. At V3 patient responded that the improvement was very significant after 6 weeks of treatment. At V3: almost perfect. At V4: Slight relapse after stop of treatment.

7	no	70.8	45.8	-25.0	-237.5	Slight improvement depending on skin location at V2 and V3. At V4, after stop of treatment quick relapse of symptoms.
8	no	14.3	23.8	100.0	100.0	Improvement at V2 (inflammatory lesions from 22 -> 18). At V3 even more improvement, with reduction in inflammatory lesion (18 -> 11). First days after stop of the treatment, relapse but quickly stabilized.
9	no	77.8	38.9	0.0	0.0	At V2, global improvement of symptoms. Inflammatory lesions drop from 18 -> 4. At V3, some relapse compared to V2. Inflammatory lesions – 4 -> 11. At V4, only 7 inflammatory lesions
10	no	20.0	20.0	53.3	66.7	At V2, global improvement on acne symptoms, both inflammatory and comedones. (infl: 5 -> 4). At V3, improvement continued but still 4 papels but no more comedones on forehead. At V4, clear improvement of acne symptoms compared to start. No inflammatory lesions.

Table S4: Dermatological Assessment of Tolerance based on clinical signs, performed by a dermatologist at all visits. Related to Figure 7.

**Verum (lactobacilli-supplemented cream)**						
**Attribute**	**Time-point**	**0-None**	**1-Very mild**	**2-Mild**	**3-Moderate**	**4-Severe**
Erythema	Tbaseline	100% (36)	0% (0)	0% (0)	0% (0)	0% (0)
	T2w	100% (36)	0% (0)	0% (0)	0% (0)	0% (0)
	T4w	97.2% (35)	0% (0)	2.8% (1)	0% (0)	0% (0)
	T8w	100% (36)	0% (0)	0% (0)	0% (0)	0% (0)
	T12w	100% (36)	0% (0)	0% (0)	0% (0)	0% (0)
Edema	Tbaseline	100% (36)	0% (0)	0% (0)	0% (0)	0% (0)
	T2w	100% (36)	0% (0)	0% (0)	0% (0)	0% (0)
	T4w	100% (36)	0% (0)	0% (0)	0% (0)	0% (0)
	T8w	100% (36)	0% (0)	0% (0)	0% (0)	0% (0)
	T12w	100% (36)	0% (0)	0% (0)	0% (0)	0% (0)
Scaling	Tbaseline	100% (36)	0% (0)	0% (0)	0% (0)	0% (0)
	T2w	100% (36)	0% (0)	0% (0)	0% (0)	0% (0)
	T4w	97.2% (35)	2.8% (1)	0% (0)	0% (0)	0% (0)
	T8w	97.2% (35)	2.8% (1)	0% (0)	0% (0)	0% (0)
	T12w	100% (36)	0% (0)	0% (0)	0% (0)	0% (0)
Dryness	Tbaseline	100% (36)	0% (0)	0% (0)	0% (0)	0% (0)
	T2w	100% (36)	0% (0)	0% (0)	0% (0)	0% (0)
	T4w	100% (36)	0% (0)	0% (0)	0% (0)	0% (0)
	T8w	100% (36)	0% (0)	0% (0)	0% (0)	0% (0)

	T12w	100% (36)	0% (0)	0% (0)	0% (0)	0% (0)
Others	Tbaseline	100% (36)	0% (0)	0% (0)	0% (0)	0% (0)
	T2w	100% (36)	0% (0)	0% (0)	0% (0)	0% (0)
	T4w	100% (36)	0% (0)	0% (0)	0% (0)	0% (0)
	T8w	100% (36)	0% (0)	0% (0)	0% (0)	0% (0)
	T12w	100% (36)	0% (0)	0% (0)	0% (0)	0% (0)
**Placebo cream**						
**Attribute**	**Time-point**	**0-None**	**1-Very mild**	**2-Mild**	**3-Moderate**	**4-Severe**
Erythema	Tbaseline	100% (31)	0% (0)	0% (0)	0% (0)	0% (0)
	T2w	100% (31)	0% (0)	0% (0)	0% (0)	0% (0)
	T4w	100% (31)	0% (0)	0% (0)	0% (0)	0% (0)
	T8w	100% (31)	0% (0)	0% (0)	0% (0)	0% (0)
	T12w	100% (31)	0% (0)	0% (0)	0% (0)	0% (0)
Edema	Tbaseline	100% (31)	0% (0)	0% (0)	0% (0)	0% (0)
	T2w	100% (31)	0% (0)	0% (0)	0% (0)	0% (0)
	T4w	100% (31)	0% (0)	0% (0)	0% (0)	0% (0)
	T8w	100% (31)	0% (0)	0% (0)	0% (0)	0% (0)
	T12w	100% (31)	0% (0)	0% (0)	0% (0)	0% (0)
Scaling	Tbaseline	100% (31)	0% (0)	0% (0)	0% (0)	0% (0)
	T2w	100% (31)	0% (0)	0% (0)	0% (0)	0% (0)
	T4w	100% (31)	0% (0)	0% (0)	0% (0)	0% (0)
	T8w	96.8% (30)	3.2% (1)	0% (0)	0% (0)	0% (0)
	T12w	100% (31)	0% (0)	0% (0)	0% (0)	0% (0)
Dryness	Tbaseline	100% (31)	0% (0)	0% (0)	0% (0)	0% (0)
	T2w	100% (31)	0% (0)	0% (0)	0% (0)	0% (0)
	T4w	100% (31)	0% (0)	0% (0)	0% (0)	0% (0)
	T8w	100% (31)	0% (0)	0% (0)	0% (0)	0% (0)
	T12w	100% (31)	0% (0)	0% (0)	0% (0)	0% (0)
Others	Tbaseline	100% (31)	0% (0)	0% (0)	0% (0)	0% (0)
	T2w	100% (31)	0% (0)	0% (0)	0% (0)	0% (0)
	T4w	100% (31)	0% (0)	0% (0)	0% (0)	0% (0)
	T8w	100% (31)	0% (0)	0% (0)	0% (0)	0% (0)
	T12w	100% (31)	0% (0)	0% (0)	0% (0)	0% (0)

Table S5: Dermatological Assessment of Tolerance based on subjective signs. Related to Figure 7.

Verum (lactobacilli-supplemented cream)						
**Attribute**	**Time-point**	**0-None**	**1-Very mild**	**2-Mild**	**3-Moderate**	**4-Severe**
Itching	Tbaseline	100% (36)	0% (0)	0% (0)	0% (0)	0% (0)
	T2w	100% (36)	0% (0)	0% (0)	0% (0)	0% (0)
	T4w	100% (36)	0% (0)	0% (0)	0% (0)	0% (0)
	T8w	100% (36)	0% (0)	0% (0)	0% (0)	0% (0)
	T12w	100% (36)	0% (0)	0% (0)	0% (0)	0% (0)
Burning	Tbaseline	100% (36)	0% (0)	0% (0)	0% (0)	0% (0)
	T2w	100% (36)	0% (0)	0% (0)	0% (0)	0% (0)
	T4w	100% (36)	0% (0)	0% (0)	0% (0)	0% (0)
	T8w	100% (36)	0% (0)	0% (0)	0% (0)	0% (0)
	T12w	100% (36)	0% (0)	0% (0)	0% (0)	0% (0)
Heating sensation	Tbaseline	100% (36)	0% (0)	0% (0)	0% (0)	0% (0)
	T2w	100% (36)	0% (0)	0% (0)	0% (0)	0% (0)
	T4w	100% (36)	0% (0)	0% (0)	0% (0)	0% (0)
	T8w	100% (36)	0% (0)	0% (0)	0% (0)	0% (0)
	T12w	100% (36)	0% (0)	0% (0)	0% (0)	0% (0)
Stinging	Tbaseline	100% (36)	0% (0)	0% (0)	0% (0)	0% (0)
	T2w	100% (36)	0% (0)	0% (0)	0% (0)	0% (0)
	T4w	100% (36)	0% (0)	0% (0)	0% (0)	0% (0)
	T8w	100% (36)	0% (0)	0% (0)	0% (0)	0% (0)
	T12w	100% (36)	0% (0)	0% (0)	0% (0)	0% (0)
Tingling	Tbaseline	100% (36)	0% (0)	0% (0)	0% (0)	0% (0)
	T2w	100% (36)	0% (0)	0% (0)	0% (0)	0% (0)
	T4w	100% (36)	0% (0)	0% (0)	0% (0)	0% (0)
	T8w	100% (36)	0% (0)	0% (0)	0% (0)	0% (0)
	T12w	100% (36)	0% (0)	0% (0)	0% (0)	0% (0)
Tightening	Tbaseline	100% (36)	0% (0)	0% (0)	0% (0)	0% (0)
	T2w	100% (36)	0% (0)	0% (0)	0% (0)	0% (0)
	T4w	100% (36)	0% (0)	0% (0)	0% (0)	0% (0)
	T8w	100% (36)	0% (0)	0% (0)	0% (0)	0% (0)
	T12w	100% (36)	0% (0)	0% (0)	0% (0)	0% (0)
Others	Tbaseline	100% (36)	0% (0)	0% (0)	0% (0)	0% (0)
	T2w	100% (36)	0% (0)	0% (0)	0% (0)	0% (0)
	T4w	100% (36)	0% (0)	0% (0)	0% (0)	0% (0)
	T8w	100% (36)	0% (0)	0% (0)	0% (0)	0% (0)
	T12w	100% (36)	0% (0)	0% (0)	0% (0)	0% (0)
**Placebo cream**						
**Attribute**	**Time-point**	**0-None**	**1-Very mild**	**2-Mild**	**3-Moderate**	**4-Severe**

Itching	Tbaseline	100% (31)	0% (0)	0% (0)	0% (0)	0% (0)
	T2w	100% (31)	0% (0)	0% (0)	0% (0)	0% (0)
	T4w	100% (31)	0% (0)	0% (0)	0% (0)	0% (0)
	T8w	100% (31)	0% (0)	0% (0)	0% (0)	0% (0)
	T12w	96.8% (30)	3.2% (1)	0% (0)	0% (0)	0% (0)
Burning	Tbaseline	100% (31)	0% (0)	0% (0)	0% (0)	0% (0)
	T2w	100% (31)	0% (0)	0% (0)	0% (0)	0% (0)
	T4w	100% (31)	0% (0)	0% (0)	0% (0)	0% (0)
	T8w	100% (31)	0% (0)	0% (0)	0% (0)	0% (0)
	T12w	100% (31)	0% (0)	0% (0)	0% (0)	0% (0)
Heating sensation	Tbaseline	100% (31)	0% (0)	0% (0)	0% (0)	0% (0)
	T2w	100% (31)	0% (0)	0% (0)	0% (0)	0% (0)
	T4w	100% (31)	0% (0)	0% (0)	0% (0)	0% (0)
	T8w	100% (31)	0% (0)	0% (0)	0% (0)	0% (0)
	T12w	100% (31)	0% (0)	0% (0)	0% (0)	0% (0)
Stinging	Tbaseline	100% (31)	0% (0)	0% (0)	0% (0)	0% (0)
	T2w	100% (31)	0% (0)	0% (0)	0% (0)	0% (0)
	T4w	100% (31)	0% (0)	0% (0)	0% (0)	0% (0)
	T8w	100% (31)	0% (0)	0% (0)	0% (0)	0% (0)
	T12w	100% (31)	0% (0)	0% (0)	0% (0)	0% (0)
Tingling	Tbaseline	100% (31)	0% (0)	0% (0)	0% (0)	0% (0)
	T2w	100% (31)	0% (0)	0% (0)	0% (0)	0% (0)
	T4w	100% (31)	0% (0)	0% (0)	0% (0)	0% (0)
	T8w	100% (31)	0% (0)	0% (0)	0% (0)	0% (0)
	T12w	100% (31)	0% (0)	0% (0)	0% (0)	0% (0)
Tightening	Tbaseline	100% (31)	0% (0)	0% (0)	0% (0)	0% (0)
	T2w	100% (31)	0% (0)	0% (0)	0% (0)	0% (0)
	T4w	100% (31)	0% (0)	0% (0)	0% (0)	0% (0)
	T8w	100% (31)	0% (0)	0% (0)	0% (0)	0% (0)
	T12w	100% (31)	0% (0)	0% (0)	0% (0)	0% (0)
Others	Tbaseline	100% (31)	0% (0)	0% (0)	0% (0)	0% (0)
	T2w	100% (31)	0% (0)	0% (0)	0% (0)	0% (0)
	T4w	100% (31)	0% (0)	0% (0)	0% (0)	0% (0)
	T8w	100% (31)	0% (0)	0% (0)	0% (0)	0% (0)
	T12w	100% (31)	0% (0)	0% (0)	0% (0)	0% (0)

Supplementary Methods

Methods S1: Registration of placebo-controlled study at ClinicalTrails.gov with additional details of the study. Related to STAR methods. (see next page)

COVID-19 is an emerging, rapidly evolving situation.
Get the latest public health information from CDC: https://www.coronavirus.gov.
Get the latest research information from NIH: https://www.nih.gov/coronavirus.

Skin Acceptance and Efficacy Assessment of a Topical Product in Acne Treatment When Compared to a Placebo.

The safety and scientific validity of this study is the responsibility of the study sponsor and investigators. Listing a study does not mean it has been evaluated by the U.S. Federal Government. Read our disclaimer for details.

ClinicalTrials.gov Identifier: NCT04216160

Recruitment Status : Active, not recruiting
First Posted : January 2, 2020
Last Update Posted : January 2, 2020

Sponsor:
YUN NV

Collaborator:
Allergisa Pesquisa Dermato-Cosmetica LTDA

Information provided by (Responsible Party):
YUN NV

Study Description

Brief Summary:
In this study the topical use of cream with live probiotic bacteria was evaluated for its efficacy in reducing acne symptoms and its effect on the skin microbiota on patients with acne vulgaris. Patients with mild to moderate acne

used the probiotic cream for 8 weeks and clinical evaluation and microbiological sampling was done at start, 2, 4, 8 and 12 weeks (after 4 weeks without use of the product). Next-Generation Sequencing is used to analyze the skin microbiota of the patients.

Condition or disease	Intervention/treatment	Phase
**Acne Vulgaris**	Other: ACN cream (**YUN**)<br>Other: Placebo cream (**YUN**)	Not Applicable

Detailed Description:
Probiotics are live micro-organisms which when administered in adequate amounts can exert a health benefit on the host. This health-promoting effects have been extensively studied in the gastrointestinal niche but it becomes more and more clear that other niches are also interesting for the potential of probiotics. Recent breakthroughs in 'next generation sequencing' (NGS) technologies are making it now possible to map the microbiota after DNA extraction, which is very interesting for bacteria that are not or difficult to cultivate. The research into the microbiota of the skin with such new NGS technologies shows that there is also an equilibrium in the skin composition of the microbiota and that there is a disturbance of the skin microbiota in acne. Acne vulgaris is known as a multifactorial condition, both hormonal triggers and environmental factors play a role. However, it is also known that Cutibacterium acnes and Staphylococcus spp. play an important role in the inflammation of the sebaceous gland follicles. Therefore, probiotic strains with antipathogenic activity against these bacteria and suitable for application to the skin are potentially able to restore the balance of the skin microbiota and reduce acne symptoms. The main objective of this study was to verify the skin acceptance and efficacy of the cream with live probiotic bacteria for acne treatment in comparison with a placebo. More specifically to evaluate the effect of the 'live' Lactobacillus species as 'active ingredient' in relation to acne symptoms and skin microbiome modulation.

Study Design

Go to

Study Type :
Interventional (Clinical Trial)

Actual Enrollment :
80 participants

Allocation:
Randomized

Intervention Model:
Parallel Assignment

Masking:
Quadruple (Participant, Care Provider, Investigator, Outcomes Assessor)

Primary Purpose:
Treatment

Official Title:
Skin Acceptance and Efficacy Assessment of a Topical Product in **Acne** Treatment When Compared to a Placebo, Under Normal Use Conditions.

Actual Study Start Date :
May 5, 2019

Actual Primary Completion Date :
November 28, 2019

Estimated Study Completion Date :
December 31, 2020

Resource links provided by the National Library of Medicine

NIH NLM

MedlinePlus related topics: Acne

U.S. FDA Resources

Arms and Interventions

Go to

Arm	Intervention/treatment
Experimental: Verum<br>Patients with mild to moderate acne using ACN Cream	Other: ACN cream (**YUN**)<br>Application of the facial cream ACN (**YUN**) twice a day, for 8 weeks +/- 2 days. Assessment before product use (T0), after 2, 4 and 8 weeks +/- 2 days of product use (T2w, T4w and T8w, respectively) and after 4 weeks +/- 2 days without product use (T12w).<br>Other Name: Topical cream with live probiotic bacteria
Experimental: Placebo<br>Patients with mild to moderate acne using the placebo cream	Other: Placebo cream (**YUN**)<br>Application of the facial placebo cream twice a day, for 8 weeks +/- 2 days. Assessment before product use (T0), after 2, 4 and 8 weeks +/- 2 days of product use (T2w, T4w and T8w, respectively) and after 4 weeks +/- 2 days without product use (T12w).

Outcome Measures

Go to

Primary Outcome Measures :

1. Change of inflammatory lesions compared to placebo. [Time Frame: baseline, 2, 4 and 8 weeks of product use and 4 weeks without product use (Total 12 weeks).]

   The subjects were assessed by a trained technician in order to perform the acne lesions counting.

2. Change of inflammatory lesions compared to baseline. [Time Frame: baseline, 2, 4 and 8 weeks of product use and 4 weeks without product use (Total 12 weeks).]

   The subjects were assessed by a trained technician in order to perform the acne lesions counting.

3. Overall tolerance of the treatment [Time Frame: Baseline to week 12]

   Dermatological Assessment of Tolerance (DAT). On all visits, the dermatologist performed an assessment of the study subjects' faces according to a 5-point scale. The physician recorded in the subject's case report form possible discomforts sensation informed.

Eligibility Criteria

Go to

Information from the National Library of Medicine

NIH NLM

Choosing to participate in a study is an important personal decision. Talk with your doctor and family members or friends about deciding to join a study. To learn more about this study, you or your doctor may contact the study research staff using the contacts provided below. For general information, Learn About Clinical Studies.

Ages Eligible for Study:
12 Years to 35 Years (Child, Adult)

Sexes Eligible for Study:
All

Accepts Healthy Volunteers:
No

Criteria

Inclusion Criteria:

- Healthy skin in the test areas;
- Subjects willing and capable to follow the study rules and a fixed schedule;
- Ability of giving consent for participation in the study;
- Subjects with good health in general and good mental condition;

- Subjects who present at least 10 inflammatory lesions;
- Oily skin on the face (minimum sebumetry value 100µg/cm² on frontal area (mean: 3 measurements)).

Exclusion Criteria:

- Pregnancy or breastfeeding;
- Subjects who present severe acne;
- Subjects who present more than two nodular lesions;
- Subjects who changed their oral contraception method up to three months before the study beginning;
- Subjects who did acne hormonal treatment less than 6 months before the study;
- Subjects who did oral isotretinoïne treatment less than 1 month before the study;
- Subjects who did topical acne treatment less than 90 months before the study;
- Subjects who did aesthetic treatment less than 6 months before the study (like laser and peeling);
- Subjects who did treatment with antibiotics within the last 4 months;
- Simultaneous participation in different studies from external research institutes on the same test sites;
- Inadequate language proficiency (spoken and written);
- Participate in the study under the influence of alcohol and/or drugs as well as addiction;
- Severe psychological disease or intellectual disability of understanding the study;
- Severe disease (heart/circulatory/liver, kidney and lungs disease, severe diabetes mellitus) or chronic infections (hepatitis, HIV);
- Immune insufficiency;
- Current use of the following topical or systemic medications: corticosteroids, immunosuppressive and anti-histaminic drugs;
- Skin diseases: vitiligo, psoriasis, atopic dermatitis;
- Confirmed allergies to cosmetic components or previous responses of intolerance after the application of cosmetic products of the same category of the investigational products;
- Other diseases or medications that might directly interfere in the study or put the subject's health under risk.

Contacts and Locations

Go to

Information from the National Library of Medicine

NIH NLM

To learn more about this study, you or your doctor may contact the study research staff using the contact information provided by the sponsor.

Please refer to this study by its ClinicalTrials.gov identifier (NCT number): ***NCT04216160***

Locations

Brazil

Allergisa Pesquisa Dermato-Cosmética Ltda
Campinas, SP, **Brazil**, 13084-791

Sponsors and Collaborators

YUN NV

Allergisa Pesquisa Dermato-Cosmetica LTDA

Investigators

Principal Investigator: Mariane Mosca **Allergisa** Pesquisa Dermato-Cosmética Ltda

More Information

Go to

Responsible Party:
YUN NV

ClinicalTrials.gov Identifier:
NCT04216160 History of Changes

Other Study ID Numbers:
074785-01/02-04-19-PRV03

First Posted:
January 2, 2020 Key Record Dates

Last Update Posted:
January 2, 2020

Last Verified:
December 2019

Individual Participant Data (IPD) Sharing Statement:

Plan to Share IPD:
No

Studies a U.S. FDA-regulated Drug Product:
No

Studies a U.S. FDA-regulated Device Product:
No

Additional relevant MeSH terms:

Acne Vulgaris

Acneiform Eruptions

Skin Diseases

Sebaceous Gland Diseases

Methods S2: Final report of placebo-controlled study from Allergisa, with additional details on the study design and population. Related to STAR methods. (see next page)

SKIN ACCEPTANCE AND EFFICACY ASSESSMENT OF A TOPICAL PRODUCT IN ACNE TREATMENT WHEN COMPARED TO A PLACEBO, UNDER NORMAL USE CONDITIONS

FINAL REPORT

TYPE OF PRODUCT: Face Creams

PRODUCTS NAMES: Study Cream Batch A / Study Cream Batch B

PRODUCTS CODE: 074785-01 / 074785-02

STUDY CODE: All-E-ES-074785-01/02-04-19

REPORT CODE: All-E-ES-074785-01/02-04-19-RFV01-Rev01

REPORT DATE: 02/11/2020

SPONSOR: YUN NV

Enterprise number: 0838.163.142

Berkenlaan 4

2630 – Aartselaar – Belgium

Telephone: +32 473 81 31 26

Study Responsibility: Ingmar Claes - Chief Scientific Officer

STUDY SITE: ALLERGISA PESQUISA DERMATO-COSMÉTICA LTDA

452, Dr. Romeu Tórtima Avenue – Barão Geraldo

Postal code: 13084-791 – Campinas – SP – Brazil

Telephone: +55 (19) 3789 -8600

Investigator in charge: Mariane Martins Mosca

SKIN ACCEPTANCE AND EFFICACY ASSESSMENT OF A TOPICAL PRODUCT IN ACNE TREATMENT WHEN COMPARED TO A PLACEBO, UNDER NORMAL USE CONDITIONS (074785-01/02)

SUMMARY

STUDY OBJECTIVES

The objective of this study was to verify the skin acceptance and efficacy of the product on the acne treatment in comparison with a placebo under normal use conditions. More specifically to evaluate the effect of the 'live' *Lactobacillus* species as 'active ingredient' in relation to acne symptoms."

- *Primary outcomes of the clinical trial:*
  1. reduction of inflammatory lesions is significantly different between study cream A and study cream B
  2. reduction of inflammatory lesions is significantly different between study cream A and study cream B
  3. overall tolerance of the treatment

- *Other parameters evaluated*
  • Clinical Efficacy Assessments and Investigator Global Assessment (IGA) of acne severity, conducted by a dermatologist;
  • Self-Assessment Questionnaires (SAS) and Skin Self-Grading (SFG) by the study subjects; • Assessment of the sebum regulatory action of a product through instrumental analysis of sebumetry;
  • Analysis of images captured by the device Visia CR® (Canfield Scientific, Inc.) – Pores, Porphyrins, Redness of the inflammatory lesions (RBX), Recovery of the Skin barrier (RBX);
  • Microbiological collection for metagenomic analysis.

METHODOLOGY

Study subjects remained in a room with controlled temperature and air relative humidity for at least 30 minutes before the initial measurements and in the interval between them. The initial clinical assessment was performed by a dermatologist to confirm the inclusion and non-inclusion criteria and to assess the initial state of the skin (Dermatological Clinical Assessment – IGA and DAT–- T0). Then, a skin sebumetry assessment was performed to confirm the eligibility of the subjects. The subjects approved underwent to an acne lesions counting by a trained technician and facial images of the subjects were obtained to assess the pores, porphyrins, redness of the inflammatory lesions and recover of the skin barrier through the device Visia CR® (Canfield Scientific, Inc.). The collection of the facial material of the subject was performed to extract the microbioma of the DNA present in human skin with the purpose of studying the microbiota metagenomics. The self-assessment (SAS) and skin self-grading (SFG) were performed by the study subjects through questionnaires. The subjects used the product for 08 weeks ± 2 days and then they suspended its use for 04 weeks ± 2 days. They were assessed before product use (T0), after 2, 4 and 8 weeks ± 2 days of product use (T2w, T4w and T8w, respectively) and after 04 weeks ± 2 days without product use (T12w). On all visits, the same assessments performed on the initial visit (T0) were repeated: dermatological assessment, instrumental measurements, images capture, questionnaires and skin material collection for metagenomics. The study subjects should record in their daily-log all the applications performed and add possible comments about the product. They were also be instructed to perform the last product application on the previous day of the assessments at the Institute.

INVESTIGATOR IN CHARGE Mariane Martins Mosca

STUDY LENGTH 12 weeks.

ALLERGISA

**FREQUENCY OF APPLICATION**	Twice daily.
**APPLICATION SITE**	Face
**POPULATION DESCRIPTION**	Female and Male subjects, aged from 12 to 33 years old (mean age: 18 years old, presenting at least 10 inflammatory lesions and oily skin on the face (minimum sebumetry value 100µg/cm² on frontal area).
**NUMBER OF SUBJECTS**	67 subjects completed the study (36 for Study Cream Batch A and 31 for Study Cream Batch B)
**ETHICS**	This study was conducted in conformance with the Declaration of Helsinki principles, the applicable regulatory requirements, including Resolution CNS nº. 466/12, and in spirit of the Good Clinical Practices (Documento de las Américas and ICH E6: Good Clinical Practice). This study was approved by the Local Ethics Committee (LEC) of Investiga - Instituto de Pesquisas, registered by the National Research Ethics Commission (CONEP).
**RESULTS / CONCLUSION**	

Primary outcomes of the clinical trial:

Acne

Comparing both products, the product Study Cream Batch A presented higher reduction of inflammatory lesions in comparison with the Study Cream Batch B. The same result was observed when the skin redness was evaluated by image analysis, relating the redness to the presence of the inflammatory lesions, in which the skin redness was lower in users of the product Study Cream Batch A than in user of the Study Cream Batch B.

Usually, higher reduction is observed in more intensive treatments, such as those generate by peelings or ingestible medication. The observed reduction is consistent with a cosmetic acne treatment of good quality presenting an average response.

Dermatological Assessment of Tolerance (DAT)

Both products can be considered safe. However, the complaints of increase of acne and oiliness from the panel of Study Cream Batch B must be evaluated with caution since the aim of the product is reduction of acne.

Study Cream Batch A

During the study 2 cases of adverse events were registered with likely nexus, totalizing 5% of the evaluated population. One subject presented subjective signals after the product use, it was itching. One subject presented objective signal after the product use, it was redness skin.

Study Cream Batch B

During the study were registered 5 cases of adverse events with likely nexus. Two subjects presented objective signs after the product use. They presented an increase of acne.Three subjects presented subjective signals after the product use, being ichting and burning and one complaint of increase of skin oiliness. One subject had sensitive skin, however since the study was not focused on population with sensitive skin, this case did not affect the product safety assessment.

Thus, only 4 adverse events were related to the product use, totalizing 10% of the evaluated population.

Skin Redness – Inflammatory Lesions – Image analysis

The product Study Cream Batch A promoted a reduction of inflammatory lesions skin redness after 4 weeks of use in relation the product Study Cream Batch B.

Recovery of the Skin Barrier - Image analysis

The product Study Cream Batch A promoted a recovery of the skin barrier after 2 and 4 weeks of use in relation the product Study Cream Batch B.

ALLERGISA

QUALITY ASSURANCE

The study was conducted according to the Resolution CNS nº. 466/2012, and in the spirit of Good Clinical Practices and in conformity with the Standard Operating Procedures of Allergisa.

Data quality is assured, considering that our personnel is trained according to the study to be carried out, our equipment is always duly calibrated, and the methods used are recognized and/or validated.

The Quality Assurance Department is in charge of auditing the Management System; and is fully available for any specific study monitoring carried out by the sponsor.

The signature below means that the study was conducted as described above.

Heliara Lopes do Nascimento
Quality Assurance Manager
02/11/2020

CONTENTS

1 ABBREVIATION LIST 8
2 INTRODUCTION 10
2.1. Acne 11
2.2. Porphyrins 11
3 OBJECTIVE 12
4 INVESTIGATIONAL PRODUCTS 12
4.1. Identification 13
4.2. Use Directions 13
4.3. Product Use Compliance Check 13
4.4. Storage 13
5 APPLICABLE ETHICAL REMARKS 13
5.1. Confidentiality of obtained data 14
5.2. Study Subjects 14
5.3. Informed Consent Form (ICF), Acceptance Form (AF) and Informed Consent Image Release (ICIR) 14
5.4. Independent Ethics Committee 14
6 STUDY PERIOD 14
7 STUDY SUBJECTS 15
7.1. Study subjects Recruitment 15
7.2. Selection and Admission of Study Subjects 15
7.3. Description of included population 15
7.4. Inclusion Criteria 16
7.5. Non-Inclusion Criteria 16
7.6. Injunction and Constraint 16
8 METHODOLOGY 17
8.1. Study Design 17
8.2. Materials and Equipment 17
8.3. Test Site 17

8.4. General Procedures 17

1.1. Procedure Schedule 26

8.5. Criteria and Procedures for Study Subjects Withdrawal 26

9 ADVERSE EVENTS 27

10 STATISTICAL ANALYSIS 28

10.1. Objective parameter (Instrumental analysis – Sebumeter and Visia CR® and Counting of Acne Lesions) 29

10.2. Ordinal and interval scaled data (Dermatological Clinical Assessment, Investigator Global Assessment (IGA) of acne severity and Self-Grading (SFG)) 29

10.3. Nominal data (Self-Assessment (SAS)) 30

11 RESULTS 31

11.1. Protocol Deviation 31

11.2. Adherence to the Study 31

11.3. Occurrences during the Study 32

11.4. *Primary outcomes of the clinical trial:* 35

11.5. Acne 35

11.6. Dermatological Assessment of Tolerance (DAT) 48

11.7. Assessments of Acne Severity (IGA) 52

11.8. Self-Assessment Questionnaire (SAS) 56

11.9. Self-Grading Assessments (SFG) 68

11.10. Sebumeter (Skin Oiliness) 119

11.11. Pores 123

11.12. Porphyrins Percentage 130

11.13. Total Surface of Redness – Inflammatory Lesions 133

11.14. Total Surface of Redness – Recovery of the Skin Barrier 136

11.15. Best Cases 141

12 CONCLUSION 145

12.1. Acne 145

12.2. Dermatological Assessment of Tolerance (DAT) 146

12.3. Skin Redness – Inflammatory Lesions 146

12.4. Recovery of the Skin Barrier 147
12.5. Assessments of Acne Severity (IGA) 147
12.6. Self-Assessment Questionnaire (SAS) 148
12.7. Self-Grading Assessments (SFG) 151
12.8. Skin Oiliness 152
12.9. Pores 152
12.10. Porphyrins 153
13 REFERENCES 154
APPENDIX 1. INFORMED CONSENT FORM 156
APPENDIX 2. ACCEPTANCE FORM - TEENAGER 164
APPENDIX 3. INFORMED CONSENT FORM AUTHORIZATION LEGAL GUARDIAN(S) 170
APPENDIX 4. INFORMED CONSENT FOR IMAGE RELEASE (ICIR) 179
APPENDIX 5. STUDY GROUP 180
APPENDIX 6. RANDOMIZATION 183
APPENDIX 7. QUESTIONNAIRES 185
APPENDIX 8. RAW DATA AND STATISTICAL ANALYSIS 201
APPENDIX 9. DNA EXTRACTION AND QUANTIFICATION 303
APPENDIX 10. PRODUCT INFORMATION 305

1 ABBREVIATION LIST

°C	Celsius Degree
μg/cm^2	Microgram per centimeter square
AF	Acceptance Form
ANOVA	Analysis of variance
ANVISA	Agência Nacional de Vigilância Sanitária (National Health Surveillance Agency).
cm	Centimeter
cm^2	Centimeter square
CNS	Conselho Nacional de Saúde (National Health Council)
CONEP	National Research Ethics Commission
CRM	Regional Council of Medicine
DAT	Dermatological Assessment of Tolerance
DNA	Deoxyribonucleic Acid
Dr.	Doctor
e.g.	For example
EDTA	Ethylenediamine Tetraacetic Acid
etc	*Et cetera*
FDA	Food and Drug Administration
GCP	Good Clinical Practices
HIV	Human Immunodeficiency Virus
ICF	Informed Consent Form
ICH	Good Clinical Practice
ICIR	Informed Consent Image Release
IEC	Independent Ethics Committee
IGA	Investigator Global Assessment
LEC	Local Ethics Committee
LSD	Least significant difference
Ltda	Limited
mL	Mililiter
mM	Milimolar
nº	Number
pH	Hydrogen Potential
RBX	Red Brown X color space
RGB	Red Green Blue color space
RH	Relative Humidity
ROI	Region of Interest
SAS	Self-Assessment of Product Performance questionnaire
SFG	Self-Grading Assessments

SP	São Paulo
Txx	Time-points os the study
UV	Ultraviolet
UV	Ultra-violet
UVA	Ultraviolet A

2 INTRODUCTION

Industry awareness and consumer's and regulatory agencies requirements caused cosmetic manufacturers to adopt procedures that lead them to know better their products: to conduct clinical tests on safety and efficacy, which are coordinated by expert physicians, before marketing a product. These procedures provide cosmetic companies with greater safety, credibility and reliability among their consumers.

Once the cosmetic product becomes freely available for the consumer, it must be safe when applied under normal or reasonably foreseeable conditions of use - ANVISA Guide for the Safety Evaluation of Cosmetic Products (Guia para Avaliação de Segurança de Produtos Cosméticos da ANVISA). For this, the raw materials used in the product formulation should be raw materials with proved safety and with established use in the cosmetic industry. In addition, the safety of the final formula must be tested before it is marketed.

The acceptance studies assess the safety of the products under real-use conditions, which allows knowing the product under the same marketed conditions. Therefore, in-use studies are performed with the finished product, before it is introduced into the market. (BARAN & MAIBACH, 1994). The objective of cosmetics safety assessment studies is to confirm the absence of risks associated with using the cosmetic product. In order to evaluate the irritation and sensitization potential of a product, a series of variables should be taken into account: components used in the formulation, ingredient concentration, absorption, amount applied, skin condition, application directions and frequency, as well as the cumulative effect (DOOMS-GOOSSENS, 1993).

According to the Good Clinical Practices, an adverse event is any untoward medical occurrence in a study subject or clinical investigation subject using a product, which does not necessarily have a causal relationship with the treatment (International Conference on Harmonisation of Technical Requirements for Registration of Pharmaceuticals for Human Use – ICH).

The contact of the skin with topical products, such as cosmetic products, may trigger different types of reactions. Among these adverse reactions, we can point out eczematous contact dermatitis, urticaria, acne and spots (SAMPAIO & RIVITTI, 2000). In general, the contact dermatitis results from two mechanisms: the primary irritation, through the action of irritant substances; or the sensitization, in the presence of an allergenic ingredient.

Tests conducted with humans are governed by very strict laws in order to protect and safeguard people. These laws vary from country to country. In Brazil, these studies are allowed, provided that they comply with the precepts of the Declaration of Helsinki and the CNS 466/12 Resolution (NATIONAL HEALTH COUNCIL, 2013).

In addition to safety, this study can also assess sensory characteristics of the product, and detect additional complaints and comments as to its "performance". Concerning the support of claims for cosmetic products, the following directives were published by COLIPA (2001):

The benefits provided by a cosmetic product must be consistent with the consumers' expectations generated by the claim; In order to evaluate if a claim is appropriate, it is necessary to take into account

the general consumers' impression concerning the presentation or the product advertisement. The *claims* must be supported by solid, clear and relevant evidences. Such evidences may result from experimental studies (biochemical/instrumental methods, sensory evaluations, technical evaluations and evaluations without the participation of study subjects: in vitro testing in cell cultures, use of hair locks, etc), and consumers' evaluations. (ASTM E 1958-06).

By performing clinical studies, the company has the opportunity to know in advance the possible considerations and complaints that may arise when the product is marketed, being able to develop strategies, such as specific training for its Consumer Service Staff before launching the product (BARAN & MAIBACH, 1994).

2.1. Acne

Acne is a very common dermatological problem, which affects a significant portion of the world population, and mainly individuals in the 11-25 age range. It is characterized by comedones, papules, pustules, cysts and/or scars, located mostly on the “seborrheic areas”, which are body regions with a higher amount of sebaceous glands – center-facial area, dorsum and thorax (VIGLIOGLIA & RUBIN, 1991).

In physiopathology, one can observe as important elements on this subject: the increase of sebaceous secretion by the gland; follicular hyperkeratosis with subsequent obstruction, causing keratin and sebum accumulation in the follicle; and bacteria colonization and inflammation (SAMPAIO & RIVITTI, 2000).

Its causes are not fully known; however, it is known that genetic aspects and emotional / immunological / hormonal / environmental factors are involved in the genesis or aggravation of the picture, as well as the alteration of the local flora, with an expansion of the bacterial population, especially the *Propioniumbacterium acnes*.

The efficacy evaluation of a product with antiacne properties may be performed through in vitro and experimental studies in laboratory animals or in human beings. Studies in animals are meant to toxicity assessment, when there are unknown drugs in the product. The in vitro studies determine the antimicrobial capacity of the product over the microorganism which are usually present in the process (*P. acne*) (VIGLIOGLIA & RUBIN, 1991). In humans, it is possible to assess the action of the products under real use conditions, considering the different types of skin.

2.2. Porphyrins

In the pilosebaceous follicles, the porphyrins are produced by *Propionibacterium acnes* (*P. acnes*), which are normal inhabitants of the human skin and the pilosebaceous ducts. Porphyrins are high fluorescent pores containing P. acnes and the porphyrins associated appear red-orange when the face skin is illuminated with UVA radiation of long wavelength. It is known that the intensity of the follicular fluorescence and its extension of the facial involvement are proportional to the density of P. acnes. Porphyrin fluorescence appears first on the nose and on the chin, and later increases the incidence on the adult age and decreases after 50 years old, possibly reflecting the sebum secretion rate.

The porphyrins may also have cytotoxic and comedogenic potential. With sun exposure, the porphyrins may liberate singlet oxygen that, in its turn, may oxidate lipids (lipidic peroxidation) on the skin,

producing irritating lipidic peroxides and cytotoxics that may cause skin damage. Thus, the presence of porphyrins on the follicular pores may accentuate or accelerate the hyperpigmentation, wrinkles and other signs of photoaging by sun exposure.

3 OBJECTIVE

The objective of this study was to verify the skin acceptance and efficacy of the product on the acne treatment in comparison with a placebo under normal use conditions. More specifically to evaluate the effect of the 'live' *Lactobacillus* species as 'active ingredient' in relation to acne symptoms."

- *Primary outcomes of the clinical trial:*
  - 1. reduction of inflammatory lesions is significantly different between study cream A and study cream B;
  - 2. reduction of inflammatory lesions is significantly different between study cream A and study cream B;
  - 3. overall tolerance of the treatment - Dermatological Assessment of Tolerance (DAT)

- *Other parameters evaluated*
  - Clinical Efficacy Assessments and Investigator Global Assessment (IGA) of acne severity, conducted by a dermatologist;
  - Self-Assessment Questionnaires (SAS) and Skin Self-Grading (SFG) by the study subjects; • Assessment of the sebum regulatory action of a product through instrumental analysis of sebumetry;
  - Analysis of images captured by the device Visia CR® (Canfield Scientific, Inc.) – Pores, Porphyrins, Redness of the inflammatory lesions (RBX), Recovery of the Skin barrier (RBX);
  - Microbiological collection for metagenomic analysis.

4 INVESTIGATIONAL PRODUCTS

Products information, as declared by the Sponsor, are described in Appendix 10. A sample of each product was cataloged and it can be found in Allergisa's files until the study final report is confirmed.

4.1. Identification

Table 1. Investigational products identification

Product Name	Type of Product	Product Code
Study Cream Batch A	Face Cream	074785-01
Study Cream Batch B	Face Cream	074785-02
YUN's SKN Wash	Shower Gel	Auxiliary Product

4.2. Use Directions

FACIAL CREAM (Study Cream Batch A / Study Cream Batch B): Apply the product twice a day, once in the morning and once at night (for example, before going to bed).
AUXILIARY PRODUCT: May be used daily for washing, in replacement of the usual soap.

4.3. Product Use Compliance Check

The compliance of product use by the subjects was checked through the daily log completed by the subjects and through the weighing of the product before and after 8 weeks of use.

4.4. Storage

All products sent by the sponsor were initially stored in the samples room at the study center, with controlled temperature and restricted access. Products release was controlled by the principal investigator or by a previously designated technical staff.

At the moment of being given the product, the subjects will be instructed on how to correctly store it and to keep it out of reach of children and/or animals.

5 APPLICABLE ETHICAL REMARKS

This study was conducted in conformance with the Declaration of Helsinki principles, the applicable regulatory requirements, including Resolution CNS nº. 466/12, and in the spirit of the Good Clinical Practice principles (Documento de las Américas and ICH E6: Good Clinical Practice).

Before the study starts, the subjects were informed about its objective, methodology and duration, and about the possibly expected benefits and the constraints related to the study. An Informed Consent Form (Appendix 1) and an Informed Consent Image Release Form (Appendix 4), written in conformity with the Declaration of Helsinki and Resolution CNS No 466/2012 and approved by the Independent Ethics Committee (IEC) of Investiga - Instituto de Pesquisas, registered by the National Ethics Commission [Comissão de Ética em Pesquisa (CONEP)] were signed by the study subjects.

In the case of subjects under 18 years old, an Acceptance Form should have been signed by the minor for their participation (Appendix 2), and the legal guardian must have been also signed a form authorizing the minor's participation (Appendix 3).

5.1. Confidentiality of obtained data

All data to be found or proved by the study results were considered as being confidential information and sponsor's property. No information - as well as all documents generated during the study - should be copied or disclosed without a previous written consent of the sponsor. All information are kept confidential until the results are published.

5.2. Study Subjects

In order to maintain confidentiality of subjects' data, all data collected were identified by a number they was given at the beginning of the study. No personal information was be disclosed together with study data. If required, the Investigator in charge could allow the study monitor to access all study-related subjects' data. This must include all documents containing the subject's clinical history for checking suitability for the study, diagnoses and any other document concerning the subject in the study.

5.3. Informed Consent Form (ICF), Acceptance Form (AF) and Informed Consent Image Release (ICIR)

The process of obtaining the ICF must confirm the voluntary nature of subjects participation in the study. All study-related aspects were explained to the subject, before they sign the ICF, AF and the ICIR. The investigator in charge is completely responsible for obtaining the ICF and AF in compliance with the specification of the GCP, Resolution CNS no. 466/2012 and the international regulatory requirements (ICH).

5.4. Independent Ethics Committee

The study will be conducted in compliance with the ICH directives for Good Clinical Practices and carried out based on the ethical principles established in the CNS Resolution no. 466/2012.

Before the beginning of the study, the protocol, the Informed Consent form (ICF), the Acceptance Form (AF) and the Informed Consent For Image Release (ICIR) will be sent for review by the Investigator Instituto de Pesquisas Independent Ethics Committee (IEC) for written approval. Any written information given to the subject and all notifications and amendments to the study will also be sent to this IEC. The unified statement issued by the IEC will be archived with the study documents kept by the study site.

The study technical documentation is in Allergisa files, where it will be archived for a 15-year period.

6 STUDY PERIOD

The total duration of the study was 17 weeks. The total duration of the study for each subject was 12 weeks ± 02 days.

- **Start Date of the first group:** 05/14/2019;
- **End Date of the last group:** 09/09/2019.

7 STUDY SUBJECTS

7.1. Study subjects Recruitment

The study subjects were recruited by the recruitment department of the Study Center that has a computerized and updated register system. The subjects registered into this system are interested in participating in clinical trials. They were contacted and asked to take part in the selection process and if they met all required criteria, they would be included in the study.

7.2. Selection and Admission of Study Subjects

During the subjects' selection for the study, the physician in charge certified that the subjects had no pathologies that could interfere with the study results. The physician is also responsible for all information contained in the subject's assessment form, by checking all inclusion and non-inclusion criteria for admission of the subject in the study.

7.3. Description of included population

The description of the population included in the study is available on the following table. The detailed description of the population per subject is available in the Appendix 5.

The list of subjects randomized by investigational product is available at Appendix 6.

Table 2. Study Population Description

Total of subjects						Age (years old)		
**Recruited[1]**	**Included[2]**	**Excluded[3]**	**Withdrawal[4]**	**Gender F**	**Gender M**	**Mínimum**	**Maximum**	**Mean**
100	79	21	00	49	30	12	33	18

[1]subjects who attended the Institute and signed the ICF.

[2]subjects that were approved in the study.

[3]Subjects that did not meet the inclusion criteria or presented any of the non-inclusion criteria.

[4]subjects that withdrew from the study after the study consent for personal reasons and were not included.

Caption: F=Female; M=Male

The description of the population randomization included in the study is available on the following table. The list of subjects randomized by investigational product is available at Appendix 6.

Table 3. Study Population Randomization

Treatment	Total of subjects			Age (years old)		
	**Included**	**Gender F**	**Gender M**	**Mínimum**	**Maximum**	**Mean**
Study Cream A	40	24	16	12	29	17
Study Cream B	39	25	14	13	33	18

7.4. Inclusion Criteria

- Healthy skin in the test areas;
- Subjects willing and capable to follow the study rules and a fixed schedule;
- Ability of giving consent for participation in the study;
- Subjects with good health in general and good mental condition;
- Any gender;
- 12 to 35 years old;
- Subjects who present at least 10 inflammatory lesions;
- Oily skin on the face (minimum sebumetry value 100μg/cm² on frontal area (mean: 3 measurements)).

7.5. Non-Inclusion Criteria

- Pregnancy or breastfeeding;
- Subjects who present severe acne;
- Subjects who present more than two nodular lesions;
- Subjects who changed their oral contraception method up to three months before the study beginning;
- Subjects who did acne hormonal treatment less than 6 months before the study;
- Subjects who did oral isotretinoïne treatment less than 1 month before the study;
- Subjects who did topical acne treatment less than 90 months before the study;
- Subjects who did aesthetic treatment less than 6 months before the study (like laser and peeling);
- Subjects who did treatment with antibiotics within the last 4 months;
- Simultaneous participation in different studies from external research institutes on the same test sites;
- Inadequate language proficiency (spoken and written);
- Participate in the study under the influence of alcohol and/or drugs as well as addiction;
- Severe psychological disease or intellectual disability of understanding the study;
- Severe disease (heart/circulatory/liver, kidney and lungs disease, severe diabetes mellitus) or chronic infections (hepatitis, HIV);
- Immune insufficiency;
- Current use of the following topical or systemic medications: corticosteroids, immunosuppressive and anti-histaminic drugs;
- Skin diseases: vitiligo, psoriasis, atopic dermatitis;
- Confirmed allergies to cosmetic components or previous responses of intolerance after the application of cosmetic products of the same category of the investigational products;
- Other diseases or medications that might directly interfere in the study or put the subject's health under risk.

7.6. Injunction and Constraint

- Do not apply any other product to the test site;
- Do not change any cosmetic habits, including personal hygiene;

- Do not perform and aesthetic or dermatological treatments on the test region during the study period;
- Do not expose to sunlight excessively.

8 METHODOLOGY

8.1. Study Design

Comparative double-blind placebo-controlled randomized clinical study. The blinding comprised: studied subjects, dermatologists, trained techinicians, investigators, statisticians and the writer of the report.

8.2. Materials and Equipment

- Visia CR® (Canfield Scientific, Inc.);
- Sebumeter SM 815 (Courage & Khazaka electronic GmbH);
- Template of 6.0cm x 5.0cm;
- Template of 4.0cm x 4.0cm;
- Template of 2.5cm x 2.5cm;
- Black cover;
- Black hair band;
- Thermohygrometer;
- Acclimated Room;
- Magnifying glass with light;
- Gloves, masks and caps;
- kit QIAamp PowerFecal DNA kit (Qiagen);
- Equipment Qubit 4 Fluorometer – ThermoFischer Scientific.

- 1 plastic bag containing:
  - 1 sterile cotton swab (Bac-Swab brand DME sterile),
  - 1 tube of 15 mL with collection saline solution (Tris, 50mM pH 7.6; EDTA 1mM pH 8.0, Twen 20 0.5%)
  - 1 tube of 2mL with DNA conserving liquid;
  - A sterile paper mask, disposable with an area of 12.5 cm² for collection;

Disposable gloves, a pair of scissors and a timer to mark the non-provided collection time.

8.3. Test Site

The products were applied to the study subjects' face.

8.4. General Procedures

The subjects were informed about the study objective, methodology and duration, and about the possible expected benefits and the constraints related to the study. Those who agreed in participated in the study signed an Informed Consent Form (Appendix 1) and the Informed Consent for Image Release (Appendix 2).

Study subjects remained in a room with controlled temperature and air relative humidity (20°C ± 2°C and 50% ± 5 RH) for at least 30 minutes before the initial measurements and in the interval between them. The initial clinical assessment was performed by a dermatologist to confirm the inclusion and non-inclusion criteria and to assess the initial state of the skin (Dermatological Clinical Assessment – IGA and DAT - T0). Then, a skin sebumetry assessment was performed to confirm the eligibility of the subjects. The subjects approved underwent to an acne lesions counting by a trained technician and facial images of the subjects were obtained to assess the pores, porphyrins, redness of the inflammatory lesions and recover of the skin barrier through the device Visia CR® (Canfield Scientific, Inc.). The collection of the facial material of the subject was performed to extract the microbioma of the DNA present in human skin with the purpose of studying the microbiota metagenomics. The self-assessment (SAS) and skin self-grading (SFG) were performed by the study subjects through questionnaires.

The subjects used the product for 08 weeks ± 2 days and then they suspended its use for 04 weeks ± 2 days. They were assessed before product use (T0), after 2, 4 and 8 weeks ± 2 days of product use (T2w, T4w and T8w, respectively) and after 04 weeks ± 2 days without product use (T12w). On all visits, the same assessments performed on the initial visit (T0) were repeated: dermatological assessment, instrumental measurements, images capture, questionnaires and skin material collection for metagenomics.

The study subjects should have recorded in the daily-log of the product use all the applications performed and possible comments about the product. They were also be instructed to perform the last product application on the previous day of the assessments at the Institute.

8.4.1. Dermatological Clinical Assessment (DAT and IGA)

The subjects were assessed by a dermatologist on all the visits. On the initial visit (T0) to verify the inclusion and non-inclusion criteria of the study and assessment of the clinical efficacy parameters and on the other visits (T2w / T4w / T8w / T12w), to assess the safety (DAT) and clinical efficacy (IGA) (Appendix 7).

8.4.1.1. Dermatological Assessment of Tolerance (DAT)

On all visits, the dermatologist performed an assessment of the study subjects' faces according to a 5-point scale (Appendix 7). The physician recorded in the subject's case report form possible discomforts sensation informed.

Subjects were also supervised by the physician throughout the study and were also assessed in case there was any symptom or sign, in order to confirm the correct and accurate use of the product and to detect possible adverse events.

Subjects were instructed to contact the study coordinator at any time, in case they presented any complaints. In these cases, they would be sent for assessment and guidance by the dermatologist in charge, who would conduct a dermatological examination, then, they would rate the reaction and follow the appropriate procedure (guidance and/ or medication and photographic documentation, when necessary).

8.4.1.2. Assessment of the Acne Severity (IGA)

This methodology of global assessment consists in the use of a scale pre-defined by the FDA (Food and Drug Administration) in 2005, for the scoring of acne severity grade (Appendix 7).

This scale has five degrees of severity (0-4), in which each class is defined by a clinically relevant and morphologically different description, with the objective of minimizing the variability among those who evaluate or diagnose (COSTA, 2005). The five characteristics are described below:

- Grade 0: Clean - no non-inflammatory and inflammatory lesions
- Grade 1: Almost clean - Almost clean; rare non-inflammatory lesions, with more than one small inflammatory lesion.
- Grade 2: Mild – Mild severity; superior to Grade 1; a few non-inflammatory lesions with no more than a few inflammatory lesions (papules / pustules only, no nodular lesions).
- Grade 3: Moderate - Moderate severity: higher than Grade 2: up to a lot of non-inflammatory lesions and there can be a few inflammatory lesions, no more than one small nodular lesion
- Grade 4: Severe - Severe; higher than Grade 3: up to a lot of inflammatory and non-inflammatory lesions, no more than a few nodular lesions.

The global assessment of the degree of acne severity was performed by a dermatologist on all the study time-points.

8.4.2. Counting of Acne Lesions

The subjects were assessed by a trained technician in order to perform the acne lesions counting and scoring on 5 face areas. The counting of the acneic lesions was performed according to the description on Table 4.

Table 4. Types of lesions assessed

Open comedones	Corneo-sebaceous masses with a visible surface (dark spots) without signs of inflammation.
Closed comedones	Slightly papulous corneo-sebaceous masses, with whitish or normal surface coloring.
Papules	Skin bumps with pinkish or reddened coloration, which might be painful.
Pustules	Skin bumps with visible pus secretion in the center.

The counting was performed with the aid of a surface magnifying glass and templates with fixed area.

The areas assessed were the malar, the mentum and the frontal area. The inflammatory lesions were counted on the whole face, while the non-inflammatory lesions were counted in specific templates, according to what is described below:

- Right and left malar (a 6.0cm x 5.0cm template placed according to the nasal wing fold in each half-face);
- Mentum (a 2.5cm x 2.5cm template placed on the mentum central portion);
- Right and left frontal area (a 4.0 x 4.0cm mold was laterally placed to the glabella).

The assessments were performed on all the study time-points.

8.4.3. Self-Assessment (SAS) and Self-Grading of the Skin (SFG) by the Study Subjects

The study subjects were instructed to assess the skin of their face through self-assessment questionnaires (SAS) and skin self-grading (SFG).

So subjects knew the meaning of each tested attribute, they were instructed by a trained technician.

While the SAS questionnaires were completed only on T2w, T4w and T8w, the SFG questionnaires were completed on all the study time-points.

8.4.4. Skin Oiliness Instrumental Evaluation with the equipment Sebumeter SM 815 (Courage & Khazaka electronic GmbH)

The skin oiliness measurements were performed by duly trained technicians, by using the equipment Sebumeter SM 815, Courage & Khazaka electronic GmbH. Measurements are based on photometry of a special translucent plastic tape, which becomes transparent in presence of lipids. This tape is applied to the skin for thirty seconds and the transparency of the tape is then measured by the device. A $1cm^2$ measurement area is used. The results provided by the device are expressed in $\mu g/cm^2$.

To confirm the inclusion criteria of oily skin, three measurements of the frontal area were performed on the subjects. The subjects who presented at least 100 $\mu g/cm^2$ of sebumetry, as mean of the three measurements, were considered included in the study.

The measurements were performed on all the study time-points.

8.4.5. Assessment with the Equipment Visia CR® (Canfield Scientific, Inc.);

Three images were obtained of the subjects' face, being 1 frontal and 2 lateral through the device Visia CR® (Canfield Scientific, Inc.). In this image, the subject's identity was preserved, and, by signing the

informed consent for image release, the subject gave his or her written consented for obtaining and releasing the image.

This device captures face digital photographs and it has been especially designed to allow the light emitted to deliver the ideal lighting for achieving good results. The system allows capturing and storing images, using visible light (Standard), polarized light flash and UV rays flash.

Subjects were instructed to keep eyes gently closed while images were being taken.

The images were taken on all study time-points.

8.4.6. Image Analysis through the Software FrameScan®

The images obtained through the equipment Visia CR® (Canfield Scientific, Inc.) were analyzed through the software FrameScan®, which is used for analysis of colorimetric images and/or quantitative morphological of digital photographic images. The software allows the extraction of images characteristics such as colorimetry measurements, luminosity, vascularization, pigmentation or homogeneity, in addition to morphology analysis of elements such as pigment spots, eyelashes, wrinkles, among others. Due to those characteristics, this software is commonly used to measure the effect of several products targeted to skincare.

In this study, parameters were used to analyze pores, porphyrins, redness of inflammatory lesions and facial skin barrier recovering of the study subjects. These parameters were calculated from a mask designed in an interest area, on the image of each subject, obtained in time-point T0 and replicated in the image obtained in time-points T2w, T4w, T8w and T12w, assuring the same area in all time-points of the study.

8.4.6.1. Pores Assessment

The Standard 2 images obtained through the equipment Visia CR® (Canfield Scientific, Inc.) were analyzed through the software FrameScan®; which is used for quantitative morphological analysis of digital photographic images. This light consists of a photography with diffuse flashes of light, leading to a slight brightness reduction. An assessment will be performed by the Software FrameScan®.

A small ROI is marked on the cheek region, where there is a high concentration of pores and the size and number of pores a recognized by the software in pixels. The image binarization was performed to delimit the affected surface by the enlarged pores.

The parameters used for the pores analysis was *number of pores* and *total surface*. The *number of pores* is the amount of pores recognized by the software in the evaluated area. The recognition occurs through the discrimination of continuous blocks of dark pixels, in which each block is recognized as one pore. The *total surface* is the sum of the pixels present in the pores on the selected area on the face. The numerical reduction of both parameters shows the pores visibility reduction on subjects.

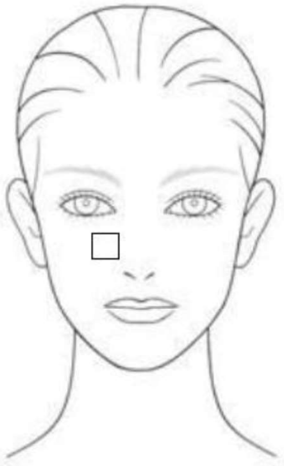

Figure 1. Example of the evaluated area for the pores analysis

8.4.6.2. Porphyrins Assessment

The images obtained through the device Visia CR® (Canfield Scientific, Inc.) were analyzed by the software ImageJ®. For this assessment, right and left lateral images and frontal UV images were analyzed. The images UV and UV2 are ideal for this type of analysis, because porphyrins present an orange-red color in this type of light (Figure 2). The image was thresholded, highlighting the porphyrins.

On areas where the porphyrins are more numerous or intense, the region of interest (ROI) is delimited and all the porphyrins on this region are recognized by this software (Figure 3). This area can be the cheek, nose or forehead, according to the characteristics of each subject, since these regions have a high incidence of porphyrins. Then, the percentage of the area occupied by the porphyrins inside the ROI is calculated (Figure 4). The decrease of the percentage of the area occupied indicated the decrease of the amount of porphyrins on the subjects' skin, which indicates, in turn, the reduction of the population of *P. acnes.*

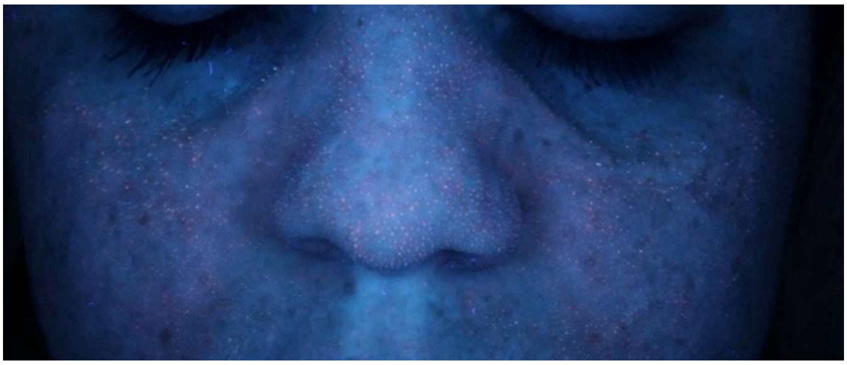

Figure 2. Example of the UV image and view of the porphyrins.

ALLERGISA

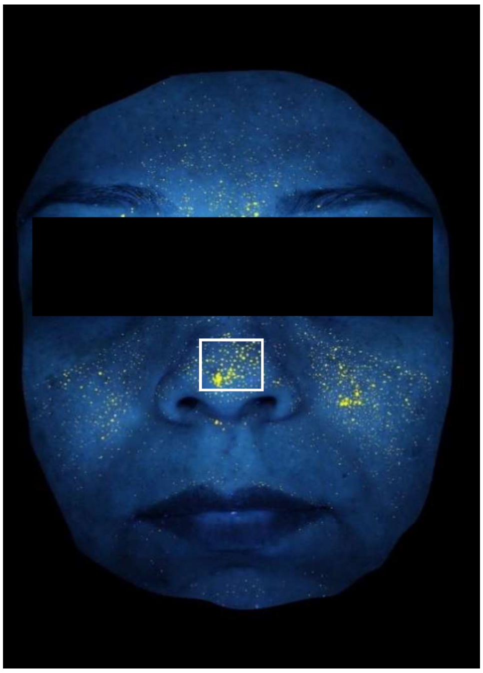

Figure 3. UV image with the mask (yellow points) after tabulation and area selected for analysis

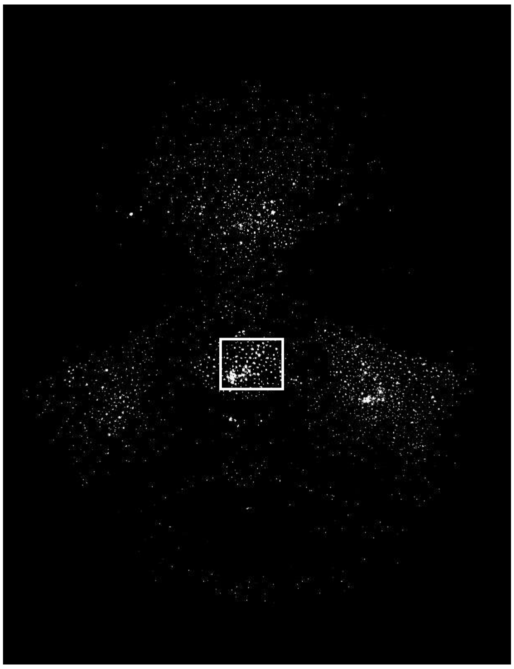

Figure 4. Image of the porphyrins mask after tabulation and area selected for analysis.

8.4.6.3. Assessment of the skin redness and recovery of Skin Barrier

The image analysis for the skin redness assessment was performed through the software FrameScan® in the module analysis Morphology of Spots, using the lateral images (left and right) with red filter RBX facial (it enhances the face vascularization). The image of the skin captured with the digital camera is composed of Red (R), Green (G) and Blue (B) and is presented on the native space RGB of the camera. The RBX transforms this RGB image into the RBX color-space where the Red and Brown channels represent hemoglobin and melanin, respectively. The red filter, used for this analysis, offers the view of the vascular conditions of each subject.

Two analysis were done through the RBX images. In one approach, the skin redness caused by skin acne was assessed, and on another approach, the integrity of the barrier function, both analyses differed from each other, due to the assessment area, where the redness is generated by different skin dysfunctions.

On the analysis of the redness caused by acne, the analyst selected a large area in one of the lateral sides of the face (in the cheek area), taken by acne lesions. The threshold was assigned to highlight acne lesions and thus, the inflammatory lesions present in the area were considered in the analysis, as shown in Figure 5. Thus, this analysis was focused on the reduction of inflammatory lesions redness.

On the analysis of integrity of the barrier function, the area selected for this analysis is positioned near the nose, chosing areas without inflammatory lesions. At the T zone the oiliness is high, which might affect the skin barrier leading to subclinic inflammatory process. Since disruption of the skin barrier present

on the stratum corneum has been correlated with some dermatosis, the reduction of the skin irritation may be related to a higher integrity of the barrier function (Elias *et al* 1999).

For both analysis, the software replicates the ROI automatically on the other images obtained in all the experimental time-points for each subject. The total area of redness was the parameter assessed and it is given in pixels. The binarization of the image (that is, the thresholding between red and white) was done to delimit through the redness (black areas after the binarization) and the portion of the skin to be assessed (Figure 5).

The parameter used for the skin irritation analysis was:

- Total Surface: This parameter is the total of the redness area, given by the sum of the number of pixels recognized as red (microvascularization). With the treatment, it was expected a reduction of this parameter, indicating that the skin irritation was reduced.

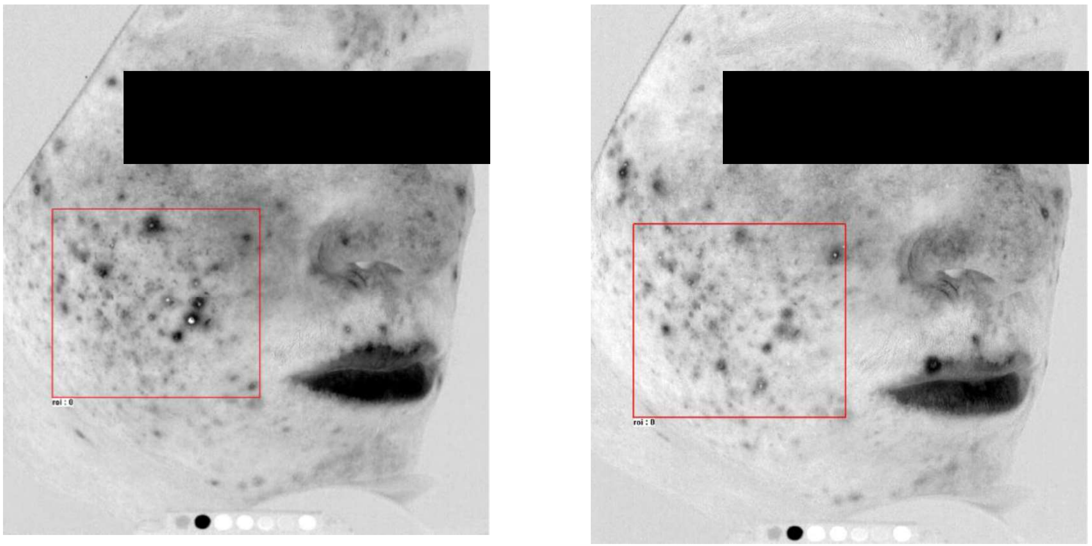

Figure 5. Example of area selected for redness analysis, comprising the inflammatory lesions on the same area, before (left) and after (right) the treatment.

8.4.7. Microbiota Collection by non-invasive Method

A human skin microbioma collection was performed as well as an extraction of the DNA of this microbioma with the purpose of studying the metagenomic of the microbiota. The collection was be performed on the subjects' faces.

The microorganisms studied are part of the natural skin microbiota. All the human material, such as the skin surface cells, were ignored and not used for analysis.

The collection of the study subjects microbiota was be done by a technician of Allergisa trained by Gentros. The collection was performed in a non-invasive way, with the help of a sterile swab (Bac-Swab brand DME sterile) soaked in saline solution (Tris, 50mM pH 7.6; EDTA 1mM pH 8.0; Twen 20 0.5%). The area of collection was standardized with the help of a sterile, flexible, plastic and individual mold, which limits the area of the collection to 12.5cm^2. The swab was gently rubbed over this restricted area by the template for 15 seconds. Right after the collection of the swab's stem, it was immersed in a tube of 2mL

with conserving liquid to avoid the degradation of the DNA and the stem cutted in a way that only the tip containing the material collect was stored in the tube. Tubes containing the swabs was identified with the subjects number. Once the collection was finished, the tubes were stored at 4°C until the microbioma DNA extraction for a maximum of one week.

The collection was performed on all study time-points.

8.4.7.1.1 Collection procedure

- 1. The date and time of the start of the procedure and the indicate area of collection on the human body scheme were recorded on the subject's file;
- 2. Surgical gloves were used during the procedure;
- 3. When opening the cotton swab, the stem did not touch where the rubbing was done;
- 4. The paper mask was brought closer to the collection region always on the position A-B in relation to who was collecting;
- 5. The cotton swab was wetted on the saline solution and rubbed during 15 seconds inside the mask-free area;
- 6. The cotton swab was inserted in the 2mL tube with conserving liquid, and cutted, with the help of scissors, the remnant stem; the tube was closed well. Attention was paid to do not let the conserving liquid spill.
- 7. The paper with the area of collection and the 2mL tube with the collection were gave back.

8.4.7.2. Extraction and Quantifying of the extracted DNA

A DNA extraction of microorganisms of the microbiota samples collected from the study subjects' faces was performed using the kit QIAamp PowerFecal DNA kit (Qiagen) according to recommendations of the manufacturer.

The microorganisms studied are part of the natural skin microbiota. All the human material, such as the skin surface cells, were ignored and not used for analysis, under the responsibility of Gentros, a partner company, specialist in metagenomic analysis.

After the extraction, the DNA was quantified by fluorimetry with the Equipment Qubit 4 Fluorometer – ThermoFischer Scientific.

The quantification result can be seen in appendix 9, table 171 with the extraction samples (microorganisms DNA). These samples were stored by Gentros until the moment of submission to the sponsor under controlled conditions (at 4ºC). The samples was submitted to the sponsor, in Belgium for analysis of skin microbioma. No type of human material was sent for the sponsor.

The samples were discarded right after analysis according to Resolution 441 of the National Health Council (Conselho Nacional de Saúde).

There was no analysis of the subjects genetic material (DNA).

1.1. Procedure Schedule

Table 5: Study schedule

	T0	T2w	T4w	T8w	T12w
Signature of the Informed Consent Form and Informed Consent For Image Release	X	-	-	-	-
Dermatological Clinical Assessment for confirmation of the inclusion and non-inclusion criteria (T0) and safety assessment (T2w / T4w / T8w / T12w)	X	X	X	X	X
Assessment of the acne degree (IGA) and assessment of the clinical efficacy by a dermatologist	X	X	X	X	X
Acne lesions count by a trained technician	X	X	X	X	X
Skin oiliness measurements through the equipment Sebumeter	X	X	X	X	X
Facial images capture with the device Visia CR®, for the record and analyses of:<br>- Pores<br>- Porphyrins<br>- RBX (redness of inflammatory lesions)<br>- RBX (recover of the skin barrier)	X	X	X	X	X
Skin self-grading (SFG) by the subjects	X	X	X	X	X
Self-Assessment questionnaire (SAS) by the subjects	-	X	X	X	X
Microbiota Collection by non-invasive Method	X	X	X	X	X
Distribution (D) / Return (R):<br>• Investigational Products<br>• Diary of Product Use	D	-	-	R	-
Assessment of product acceptance and compliance by checking Daily Log	-	X	X	X	-
Investigational products weighing	X	-	-	X	-
Assessment of Adverse Events (if applicable)	-	X	X	X	X

8.5. Criteria and Procedures for Study Subjects Withdrawal

The exclusion of a study subject by the investigator could have occurred due the following reasons:

- Study subjects not included: subjects who signed the ICF, but who did not meet the inclusion and exclusion criteria of the study;
- Subjects who present - at the Investigator's discretion - any problem that would prevent the product application from continuing, at any time during the study;
- Consent withdrawal by the study subject, regardless of the reason;
- Lack of adhesion of the study subject to the study. A significant lack of adhesion will be recorded if the subject does not visit the study center for assessments;
- Serious Adverse Event;

- Concurrent disorder or treatment: any pathological process or treatment that occurs during the study period and that might interfere with the study product, such as a medication interaction or masking of results.

Those subjects removed from the study by the investigator would be assessed in case they present any event possibly related to the study, even after their removal. Those subjects removed due to occurrence of an adverse event would be continually assessed until the case is completely resolved.

Those subjects who are removed from study after the inclusion stage were not replaced.

9 ADVERSE EVENTS

An adverse event is any untoward medical occurrence in a patient or clinical investigation subject administered a product and that does not necessarily have a causal relationship with this treatment. An adverse event can therefore be any unfavorable and unintended sign (including an abnormal laboratory finding), symptom, or disease temporally associated with the use of the investigational product (adapted from ICH, 1996).

According to the Good Clinical Practices (ICH, 1996), a Serious Adverse Event is any untoward medical occurrence that at any dose:

- Results in death;
- Is life-threatening;
- Requires inpatient hospitalization or prolongation of existing hospitalization;
- Results in persistent or significant disability/incapacity;
- Is a congenital anomaly/birth defect.

Thus any new sign, symptom or disease, or clinically significant worsening compared to the condition at the first visit, should be considered an Adverse Event. Lack of clinical or self-assessment of a cosmetic product or drug is not considered an Adverse Event.

Clinical signs and dermatological or systemic diseases observed during the selection process of the study subjects are not considered as Adverse Events. This information is recorded on the medical assessment form as a reason for non-inclusion and the subjects are then not included in the study.

The adverse events occurred as a result of incorrect product use (either cosmetics or drugs products) - such as inappropriate frequency or incorrect application - will be considered as adverse events that do not interfere with the product evaluation, since the subject- in this situation - does not follow the correct use directions stated on the product label.

An Adverse Event Form is completed for all events occurred. The study sponsor is notified of an adverse event through a Notification of Occurrence form sent by electronic mail or in the Final Study Report.

In case there is an adverse event with doubtful causal nexus, an investigation process is initiated in order to determine if such event is or is not related to the study or investigational product.

The procedures adopted during the event investigation are defined by the physician in charge, based on the nature of the reaction, the subject's medical history and on factors that may interfere with the occurrence of the event, such as medication or other concomitant disorders.

When closing a final diagnosis, the relationship of an Adverse Event to the study or study products can be defined by using one of the following expressions:

For the conclusion of the final diagnosis, the relation of an Adverse Event can be defined using the decision tree Colipa (2016), according to the following description:

- Very likely: Only cases in which the clinical condition is considered to be evocative will be classified as a very likely nexus, the following conditions occurring together: (i) the temporality of the facts is compatible with an adverse reaction to cosmetics and (ii) there is a laboratory test that confirm the relationship with the test product (e.g. positive patch test for the test product).
- Likely: The cases in which the clinical condition is considered to be evocative will be classified as likely causal nexus, occurring with the following conditions together: (i) the temporality of the facts is compatible with an adverse reaction to cosmetics and (ii) there is no laboratory test to confirm the relationship with the test product (e.g. diagnosis of contact dermatitis, without patch test, cosmetic acne - there are no laboratory tests to confirm the relationship with the product).
- Not clearly attributable: Cases in which the clinical scenario is not considered to be evocative or the chronology is not clearly compatible or unknown, will be classified as nexus not clearly attributable.
- Improbable: The following two cases are associated with an improbable nexus: the clinical scenario is not considered to be evocative, the chronology is not clearly compatible or unknown, and the result of the investigation with the test product is negative (patch test or re-exposure).

Excluded: The cases in which the diagnosis corresponds to a dermatosis of well-known cause and / or known to be caused by the use of cosmetics will be classified as excluded nexus (e.g. vitiligo, tineas, pityriasis rosea, pityriasis versicolor, psoriasis, folliculitis, solar melanose, ephelides, among others), when there is no correlation between the subject's complaint and the use of a cosmetic product (for example: muscle pain, lack of appetite, stomach pain, diarrhea, insect bites, among others) or the chronology is clearly incompatible with an adverse reaction to the cosmetic product (for example: there is no improvement in the scenario, even with the interruption of the product; there is relapse of the scenario, without the reintroduction of the product; the signs and symptoms started before the start of the product use).

10 STATISTICAL ANALYSIS

Exploratory data analysis was performed (descriptive statistics), according to Guideline for the Statistical Analysis of Efficacy Studies Version 1.4 – October 2018.

The normality in the study was verified with the Shapiro Wilks test with significance level of 1%.

Within one parameter, parametric and non-parametric analysis methods were not be mixed.

The number of subjects in the study was 36 for Study Cream Batch A and 31 for Study Cream Batch B.

Confidence level: 95%

Softwares: XLSTAT 2019 and Minitab 14.

The raw data and statistical analysis were available at Appendix 8.

The detailed statistical approach for each type of data are described below.

10.1. Objective parameter (Instrumental analysis – Sebumeter and Visia CR® and Counting of Acne Lesions)

Comparison among treatments, if a baseline measurement at T0 was performed:

Analysis of the baseline situation (pre-test):

Comparisons between baseline between treatments were performed through pairwise Student t tests using original data. When normality was rejected, the Wilcoxon Signed rank test was performed. The bilateral hypothesis was used.

Analysis after application (post-test)

For each point in time, Student t test for independent samples using the differences to baseline T0 was performed.

Comparison between points in time:

For each treated test site, a repeated measure ANOVA with the factor point in time as qualitative variable was performed using original data. The post hoc pairwise comparisons used was the Fisher's least significant difference (LSD) test.

10.2. Ordinal and interval scaled data (Dermatological Clinical Assessment, Investigator Global Assessment (IGA) of acne severity and Self-Grading (SFG))

Comparison among treatments, if a baseline measurement at T0 was performed:

Analysis of the baseline situation (pre-test):

Comparisons among treatments at baseline T0 were be performed via Wilcoxon rank sum (Mann-Whitney) tests using original data.

Analysis after application (post-test)

For each point in time, comparisons among treatments were performed via Wilcoxon rank sum (Mann-Whitney) tests using differences to baseline T0.

Comparison between points in time:

For each treatment, comparison among points in time were performed via pairwise Wilcoxon's signed rank tests using original data.

10.3. Nominal data (Self-Assessment (SAS))

In case of the yes/no scale with “do not know” only the agreement level is of particular interest. Therefore, it is sufficient to represent only the agreement level in the results section. More detailed results like the absolute and relative frequencies for all categories ”yes”, “no” and “do not know” were presented in the appendix 8.
